# Fish in the sea: Number, characteristics, and partner preferences of unmarried Japanese adults - analysis of a national survey

**DOI:** 10.1371/journal.pone.0262528

**Published:** 2022-02-02

**Authors:** Cyrus Ghaznavi, Haruka Sakamoto, Shuhei Nomura, Anna Kubota, Daisuke Yoneoka, Kenji Shibuya, Peter Ueda

**Affiliations:** 1 Department of Global Health Policy, Graduate School of Medicine, The University of Tokyo, Tokyo, Japan; 2 Medical Education Program, Washington University School of Medicine, St. Louis, Missouri, United States of America; 3 Department of Tropical Medicine and International Affairs, Tokyo Women’s Medical University, Tokyo, Japan; 4 Department of Health Policy and Management, School of Medicine, Keio University, Tokyo, Japan; 5 Tokyo Foundation for Policy Research, Tokyo, Japan; 6 Division of Biostatistics and Bioinformatics, Graduate School of Public Health, St. Luke’s International University, Tokyo, Japan; 7 Clinical Epidemiology Division, Department of Medicine, Solna, Karolinska Institutet, Stockholm, Sweden; Hitosubashi University, JAPAN

## Abstract

**Background:**

A large proportion of adults in Japan remain unmarried even though they intend to marry during their lifetime. To provide data for policy makers and those searching for partners in the Japanese marriage market, we estimated the number and characteristics of unmarried women and men with marriage intention and assessed their partner preferences. Based on the findings, we hypothesized regarding potential mismatches between the individuals available in the marriage market and the type of partners they are looking for.

**Methods:**

We used data from the National Fertility Survey (2015), a nationally representative survey in Japan, and included 20,344 participants aged 18–49 years, of which 6,568 were unmarried with marriage intention. We estimated the total number of unmarried women and men who intend to marry, extrapolated their characteristics to the Japanese population, and assessed their partner preferences, as well as their ideal age of marriage and the ideal age of their partner.

**Results:**

In 2015, there were 8.48 million unmarried women and 9.83 million unmarried men aged 18–49 years with marriage intention in Japan. Surpluses of around 600,000 men were observed in non-densely inhabited areas (men-to-women ratio: 1.31) and in the Kanto region (1.23). Most of the women and men in the marriage market had annual incomes lower than 3,000,000 JPY (28,000 USD) and only 263,000 women (3%) and 883,000 men (9%) had an income of 5,000,000 JPY (47,000 USD) or more; 167,000 men (2%) had an income of 7,000,000 JPY (66,000 USD) or more, with roughly three-quarters of them having a university degree. When asked about eight items that one may consider in a potential partner, the proportion of women listing an item as “important” tended to be larger than those of men across all items (education, occupation, finances, personality, mutual hobbies, cooperation/understanding regarding one’s work, and attitude towards/skills in housework and childrearing) except appearance. The largest differences were observed for finances (proportion of women vs. men listing the item as “important” or “would consider:” 94.0% vs. 40.5%, p<0.001), occupation (84.9% vs. 43.9%, p<0.001), and education (53.9% vs. 28.7%, p<0.001). While women, on average, preferred men who were around 1–3 years older than themselves, men preferred women around their own age until the age of 26 years, at which point men preferred women who were younger than themselves, with the preferred age difference increasing substantially with age. As such, the number of men preferring a younger partner was larger than the number of women who preferred an older partner.

**Conclusions:**

By providing data on the number, characteristics and partner preferences of individuals in the marriage market, our study could inform decisions for those searching for marriage partners in Japan. Moreover, we hypothesize that mismatches in geographical location, the supply-demand disparity for partners with higher income, and age preferences could partly explain the large number of Japanese women and men who remain unmarried despite intending to get married. Further studies are needed to assess if, and to what extent, the identified mismatches may affect marriage rates.

## Introduction

Japan is facing low birth rates and a rapidly ageing population. Given that only around 2% of children born in Japan have unmarried parents [1], the large and increasing proportion of adults that remains unmarried has received significant attention. In 2015, 24% of women and 35% of men aged 35–39 years had never been married [2], even though the National Fertility Survey reports that close to 90% of unmarried women and men aged 18–34 years responded that they intend to get married in their lifetime [3].

The process of searching for a partner is double-sided: individuals must choose a partner and then be chosen in return. Thus, those who are unmarried despite their wish to get married may not be interested in forming unions with the partners (if any) available to them at the time. Indeed, in the National Fertility Survey, around half of unmarried women and men aged 25–34 years with an intention to marry in their lifetime listed “have not met a suitable partner” as a reason for staying single [3]. As such, one could hypothesize that potential “mismatches” in what is available in the marriage market (supply) and what individuals look for (demand) may help explain why marriage does not occur [4].

When choosing a partner, it is widely accepted that individuals use simple heuristics aiming to “satisfy” some criteria in potential partners, such as age range, financial resources, personality, and appearance, in order to predict the utility of a potential union [3–6]. In the Japanese marriage market, income and education are considered to be important spouse-selection criteria [4, 7]. Higher income is robustly associated with marriage among Japanese men [8], and while popular notion suggests that women with high income and education are less likely to marry [9, 10], higher income (before marriage) and education have been associated with an increased likelihood of marriage among Japanese women [11]. Historically, women have tended to marry partners with equal (homogamy) or higher (hypergamy) income and education than themselves [12–14], and this preference seems to be widespread among many Japanese women [7, 15]. For example, an analysis of survey data (2017) from 1,516 unmarried 25-34-year-old Japanese women found that around 9 in 10 women prefer that their potential partner’s income be no lower than their own, with this pattern persisting in higher income groups. Among women with an annual income of 4,000,000 JPY (38,000 USD) or more, 83% wanted their husband to have an income that was at least as high as their own [7].

Many studies have examined factors associated with marriage in Japan [4, 8, 11, 16]. However, to assess potential mismatches between supply and demand in the Japanese marriage market, estimates of the absolute number of women and men who satisfy certain criteria, such as income, education, and age, are needed. Moreover, partner preferences among men and women and how well they correspond with whom is available in the market merit investigation. Such information is also crucial for individuals active in the marriage market, as the odds of finding what they are looking for are determined by the absolute number of potential partners that satisfy their spouse-selection criteria and the competition for such partners.

In this study, we used the 2015 National Fertility Survey to describe the characteristics and preferences of individuals available in the Japanese marriage market, with a focus on income, education, and age. First, we assessed how the likelihood of being married and having an intention to marry varied across sociodemographic characteristics. We then estimated the absolute number and described the characteristics of unmarried women and men with an intention to marry. Finally, we analysed factors considered to be important in a potential partner, assessed age preferences, and explored potential mismatches between supply and demand in the Japanese marriage market.

## Methods

### Data sources

The 2015 National Fertility Survey of Japan has been described in detail elsewhere [3, 17]. In brief, the survey is carried out by The National Institute of Population and Social Security Research (IPSS), under the Japanese Ministry of Health, Labour and Welfare, to collect nationally representative data on topics related to marriage and childbirth. Stratified cluster sampling with districts in the Population Census of Japan as primary sampling units was used to conduct two national sub-surveys: one for married couples in which the wife was under 50 years of age (with the wife providing information about the husband) and one for unmarried women and men aged 18–49 years. Participants were provided with a self-administered questionnaire during a home visit; the questionnaire was returned upon completion in a sealed envelope during a follow-up visit. The response rate for the 2015 survey was 76.5% among unmarried respondents and 87.8% among married couples [3]. Although the response rate was high, the generalizability of the survey should be considered in the context of potential bias due to non-response that is associated with the investigated variables.

Information on the number of individuals in the Japanese population by age, sex, and marital status was obtained from the Population Census of Japan (2015) [2]. We used these data for calculation of sample weights and extrapolation of the survey findings to the population of Japanese women and men, aged 18–49 years, as described below.

### Study population

We included all survey participants, aged between 18 and 49 years (n = 20,334). Unmarried participants were asked whether they had the intention to marry in their lifetime, as described in the S1 File. We excluded unmarried participants who had not answered this question. The proportion of excluded participants was 2.9% (7.3% of unmarried participants) among women and 3.3% (7.4% of unmarried participants) among men; in total, 10,539 (3,941 unmarried) women and 9,805 (4,168 unmarried) men were included in the analyses. The excluded participants had lower education and income and were less likely to reside in the Kanto region, as compared with those included in the analyses (S1 Table of S1 File).

Sample weights based on age, sex, and marital status (married; unmarried) were used to adjust for differential probabilities of non-response and for missing data on the question regarding marriage intention among those unmarried, as described in the S1 File. Sample weights were used when conducting analyses of both married and unmarried respondents, but were not used when analysing unmarried respondents alone as each sub-survey is nationally representative without weighting. In a sensitivity analysis described below, we used sample weights which accounted for history of marriage among the unmarried.

### Statistical methods

All analyses were stratified by sex and performed in Stata version 15.0 (StataCorp LP, College Town, TX) and R version 3.3.2. We used the Chi-squared test to assess differences in proportions and the t-test for comparisons of continuous variables. We present income in JPY and provide rough estimates of the corresponding values in USD, rounded to 1,000s, based on the conversion rate 1,000 JPY = 9.47 USD, as of October 2020. Groups with less than 5 observations were shown as <5.

First, we determined the proportion of Japanese women and men, aged 18–49 years, who were married, unmarried with marriage intention, and unmarried with no such intention. In each of the age groups 18–24, 25–39, and 40–49 years, we assessed these proportions across participant characteristics, using variables selected *a priori*, including education, occupational status, annual income, region of residence, and population size/density of residence (definitions and categorization in S2 Table of S1 File). Using data on the number of women and men in Japan from the Population Census of Japan [2], we then estimated the number of unmarried women and men with marriage intention in each age group. We also performed these analyses using five categories [(1) married; never-married (2) with and (3) without marriage intention, and previously married (4) with and (5) without marriage intention].

Next, we described the characteristics of unmarried survey participants with the intention to get married in their lifetime, using variables selected *a priori* (S3 Table of S1 File), which in addition to the variables listed above, also included previous marriage and preferred life course of the wife after marriage (working or homemaker). We extrapolated these characteristics to the Japanese population with the intention to marry (total age range and by age group) and calculated the difference between the number of men vs. number of women, as well as the men-to-women ratio, in each sociodemographic category. Never-married and previously married individuals with marriage intention were analysed as one group as both groups were available in the marriage market and the aim of the study was to describe the individuals available in the market regardless of their marriage history. We complemented these analyses with analyses stratified by previous marriage history in order to describe potential differences in the characteristics of the two groups.

The combination of high income and education are considered desirable qualities in men in the Japanese marriage market, and it has been suggested that many unmarried women have high income and education [9, 10]. Thus, to provide data on the number of individuals by income level, we estimated the number of individuals with an income equal to or higher than income cut-offs with 1,000,000 JPY (9,000 USD) increments (0 to <1,000,000; 1,000,000 to <2,000,000; 2,000,000 to <3,000,000; 3,000,000 to <4,000,000; 5,000,000 to <6,000,000; 6,000,000 to <7,000,000; and ≥7,000,000 JPY). Prior work suggests that some individuals, predominantly women, prefer partners with equal (homogamy) or higher (hypergamy) income and education than themselves [7, 9, 18]. For each income and education cut-off, we therefore calculated the number of individuals with an income equal to or lower than the cut-off divided by the number of opposite sex individuals with an income equal to or higher than the cut-off. These numbers present the women-to-men and men-to-women ratios, respectively, for each income level in a situation where all individuals would prefer an opposite-sex partner with an income equal to or higher than themselves, i.e., the number of individuals with marriage intention per available potential partner for hypergamy or homogamy by income level. For example, this number for single women making between JPY 2–3 million is equal to the number of women who make JPY 2–3 million or lower divided by the number of men who make JPY 2–3 million or higher. Accordingly, a higher number corresponds to more competition for the partners available for hypergamy or homogamy. In addition, we calculated these numbers for a scenario in which all individuals would limit their partner search to individuals with a university education; in this scenario, we calculated the number of individuals with an income equal to or lower than the cut-off divided by the number of opposite sex individuals with university education and an income equal to or higher than the cut-off. We performed these analyses for those aged 25–49 years as the income differences are more pronounced in this age group; we also performed the analyses using the full age range.

Unmarried survey participants with an intention to get married were provided with a list of eight items (education, occupation, finances, personality, appearance, mutual hobbies, cooperation/understanding regarding one’s work, and skills in and attitude towards housework and childrearing) that one could consider when choosing a partner. Participants were asked to select how important they consider each item to be when choosing a partner: “important,” “would consider,” or “does not matter.” (S1 File) For each item separately, we assessed the proportion of individuals that answered “important” or “would consider” across participant characteristics (S1 File). We also assessed the mean (standard deviation [SD]) number of items listed as “important” across participant characteristics and compared the responses of women vs. men. These analyses were performed in the total population (18–49 years old) and by age group (18–24 years and 25–49 years). In addition, we also conducted these analyses for never- and previously-married respondents in the total population (18–49 years old). Because those who have been previously married might be underrepresented in the National Fertility Survey, we assessed whether the use of sample weights which accounted for age and marital status (categorized into married, never-married and previously married (including divorced and bereaved)) based on data from the Population Census would affect the findings materially. The characteristics of unmarried women and men with marriage intention and the findings regarding items considered as “important” or “would consider” when choosing a partner were almost identical in analyses with and without the use of such sample weights (data available upon request).

Unmarried survey participants were also asked at which age they would ideally get married, and the ideal age of their partner at the time of marriage, as described in S1 File. For each combination of ideal age at marriage and ideal age of the partner, we estimated the number of Japanese individuals with the intention to marry who had such an age preference. This analysis was also separately conducted for those who were never- and previously-married. We also performed these analyses using categories of preferred age differences (husband ≥7 years older; husband 3 to <7 years older; husband 1 to <3 years older; same age; wife 1 to <3 years older; wife ≥3 years older). We calculated the mean ideal age of the partner by the respondent’s ideal age at marriage (1-year increments) and assessed the linear correlation by calculating the Pearson correlation coefficient. In post-hoc analyses using data from married couples, we assessed the age at which the husband and wife got married; these analyses were performed to compare the age at marriage among married couples with the preferred age of marriage among unmarried individuals. The Regional Ethics Committee at The University of Tokyo, Japan approved the study.

## Results

### Marriage status and marriage intention

**Fig 1:**
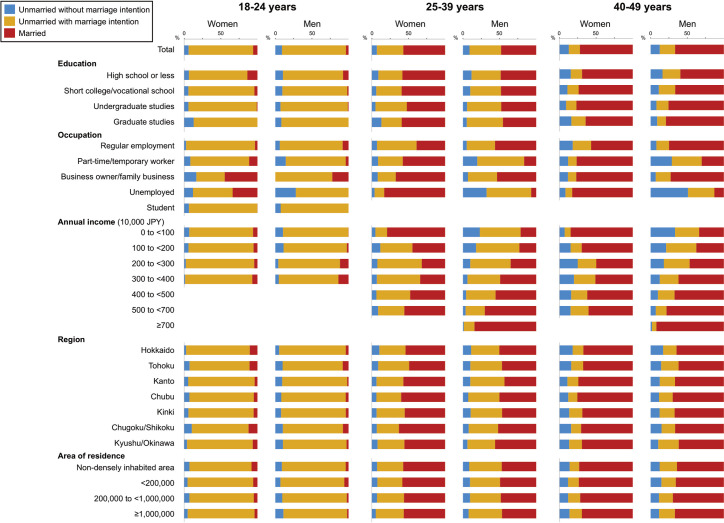
Proportion of women and men who were married, unmarried with marriage intention, and unmarried without marriage intention by sociodemographic characteristics.

The proportions married, unmarried with marriage intention, and unmarried without marriage intention across participant characteristics and age group are shown in Fig 1 and in S1 File (S4 and S5 Tables). Among women aged 18–49 years old, the relationship between income and marriage was U-shaped, with those having the lowest and highest incomes being most likely to be married. In the age group of 40–49 years, the lowest proportion of married women were observed among those who had an income of 2,000,000 to <3,000,000 JPY ([19,000 to <28,000 USD], 50%) and 3,000,000 to <4,000,000 JPY ([28,000 to <38,000 USD], 51%); in the highest income group (≥5,000,000 JPY [≥47,000 USD]) this proportion was 60%. In the same age group, women with an undergraduate degree were the most likely to be married (77%) while those with a high school degree or less (69%) and a graduate degree (64%) were the least likely to be married. The proportion of married men increased while the proportion with no marriage intention decreased with higher annual income. Of men with an annual income of ≥7,000,000 JPY (≥66,000 USD), 84% (25–39 years) and 92% (40–49 years) were married. Men who were unemployed and those with part-time/temporary employment were more likely to have no marriage intention than those with regular employment. Among men aged 40–49 years, the proportion married increased with level of education. The proportion of adults categorized according to marriage status, previous marriage and marriage intention are shown in S1 File (S6–S13 Tables). While the proportion of never-married women (85%) and men (81%) with marriage intention was similar, a larger proportion of previously married men (73%) than women (59%) had such an intention (S6 and S7 Tables of S1 File).

### Unmarried women and men with an intention to marry

**Fig 2:**
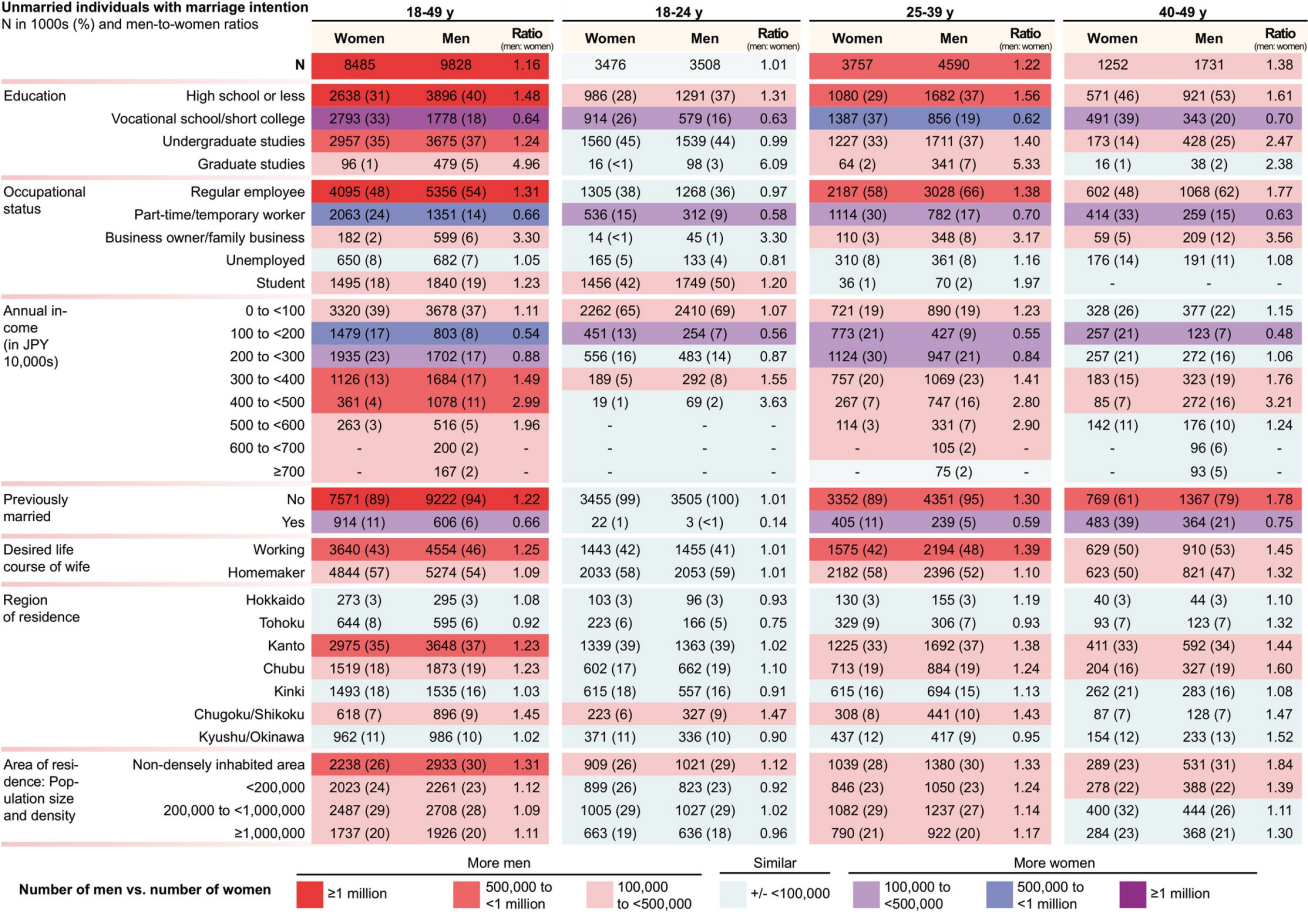
Number and characteristics of unmarried Japanese women and men with marriage intention, aged 18–49 years. Numbers are shown in N thousands (%).

Of the 22.81 million women aged 18–49 years in Japan in 2015, 12.36 million (54%) were married, 8.48 million (37%) were unmarried with marriage intention, and 1.96 million (9%) were unmarried without marriage intention. Of the 23.12 million men, 10.95 million (47%) were married, 9.83 million (43%) were unmarried with marriage intention, and 2.33 million (10%) were unmarried without marriage intention. The number of unmarried women and men with marriage intention, across age groups and sociodemographic characteristics, are shown in Fig 2. Overall, there were 1.34 million more unmarried men than women who intended to get married. The disparity was largely driven by those aged 25–39 years (4.59 million men vs. 3.76 million women) and 40–49 years (1.73 million vs. 1.25 million). While a larger proportion of men than women had no marriage intention, the surplus of men was a result of the larger number of men than women in the population, and the smaller proportion of men who were married (in part a result of more women than men being married to a partner older than 49 years).

When analysed by sociodemographic characteristics, there was greater than one million more men than women who had an educational level of high school or less and a similar difference in the number of women vs. men was observed for regular employees. In addition, there were large surpluses of around 600,000 men in non-densely inhabited areas (men-to-women ratio 1.31) and in the Kanto region (of which Tokyo prefecture is a part) (men-to-women ratio 1.23). While the number of men tended to be similar or higher across most sociodemographic characteristics, a larger number of women than men had gone to vocational school or short college, were part-time/temporary workers, had an income of between 1,000,000 (9,000 USD) and <3,000,000 JPY (<28,000 USD), and had previously been married (Fig 2). Both men and women that had never been married were more likely to have higher educational levels, be a student, and have low income compared to their previously-married counterparts. (S6 and S7 Tables of S1 File) However, the previously married constituted only 11% of the unmarried women with marriage intention (39% in the 40–49 y age group) and 7% of the unmarried men with marriage intention (21% in the 40–49 y age group). Therefore, characteristics of the total population of unmarried individuals were largely similar to those never-married.

Table 1 shows the number of unmarried women and men aged 25–49 with an intention to marry who had an income lower than cut-offs with 1,000,000 JPY increments, the number of opposite sex individuals with an income equal to or higher than the cut-off, and the number of individuals per opposite sex individuals available for hypergamy or homogamy. Of the women aged 25–49 years with marriage intention, only 616,000 (12%) had an annual income of ≥4,000,000 JPY (≥38,000 USD), and 260,000 (5%) had an income of ≥5,000,000 JPY (≥47,000 USD), with around half of these women having a university degree (Fig 2 and Table 1). Of the men aged 25–49 years, 879,000 (14%) had an annual income of ≥5,000,000 JPY (≥47,000 USD) and 168,000 (3%) had an annual income of ≥7,000,000 JPY (≥66,000 USD). From the women’s perspective, the number of women per man available for homogamy or hypergamy was 2.53 for women earning <5,000,000 JPY and 13.68 for women earning <7,000,000 JPY. The corresponding numbers for these cut-offs if only considering men with a university education as potential partners were 4.86 and 21.69. The number of women and men with the intention to marry by income cut-offs and the corresponding number of available partners for homogamy or hypergamy in the age group 18–49 years are shown in S1 File (S14 Table).

**Table 1:** Number of unmarried women and men aged 25–49 years per partner available for hypergamy or homogamy.

Women’s perspective	Women		Men				
	Income level	(A) N total (in 1000s)	Income level	(B) N total (in 1000s)	(C) N with university education (in 1000s)	(A)/(B) N women per man available for homogamy/hypergamy	(A)/(C) N women per man available for homogamy/hypergamy, including education
	0 to <100	1064	≥0	6347	2550	0.17	0.42
	100 to <200 or lower	2108	≥100	5075	2118	0.42	1.00
	200 to <300 or lower	3508	≥200	4523	1961	0.78	1.79
	300 to <400 or lower	4461	≥300	3299	1574	1.35	2.83
	400 to <500 or lower	4817	≥400	1902	991	2.53	4.86
	500 to <600 or lower	4986	≥500	879	502	5.67	9.93
	600 to <700 or lower	5052	≥600	369	233	13.68	21.69
	≥700 or lower	5077	≥700	168	130	30.19	39.03
Men’s perspective	Men		Women				
	Income level	(A) N total (in 1000s)	Income level	(B) N total (in 1000s)	(C) N with university education (in 1000s)	(A)/(B) N men per woman available for homogamy/hypergamy	(A)/(C) N men per woman available for homogamy/hypergamy, including education
	0 to <100	1272	≥0	5077	1517	0.25	0.84
	100 to <200 or lower	1824	≥100	4014	1306	0.45	1.40
	200 to <300 or lower	3048	≥200	2969	1145	1.03	2.66
	300 to <400 or lower	4445	≥300	1569	739	2.83	6.01
	400 to <500 or lower	5468	≥400	616	311	8.87	17.57
	500 to <600 or lower	5978	≥500	260	130	23.00	45.81
	600 to <700 or lower	6179	≥600	91	53	67.62	117.01
	≥700 or lower	6347	≥700	25	17	255.15	378.79

### Factors considered in a potential partner

**Fig 3:**
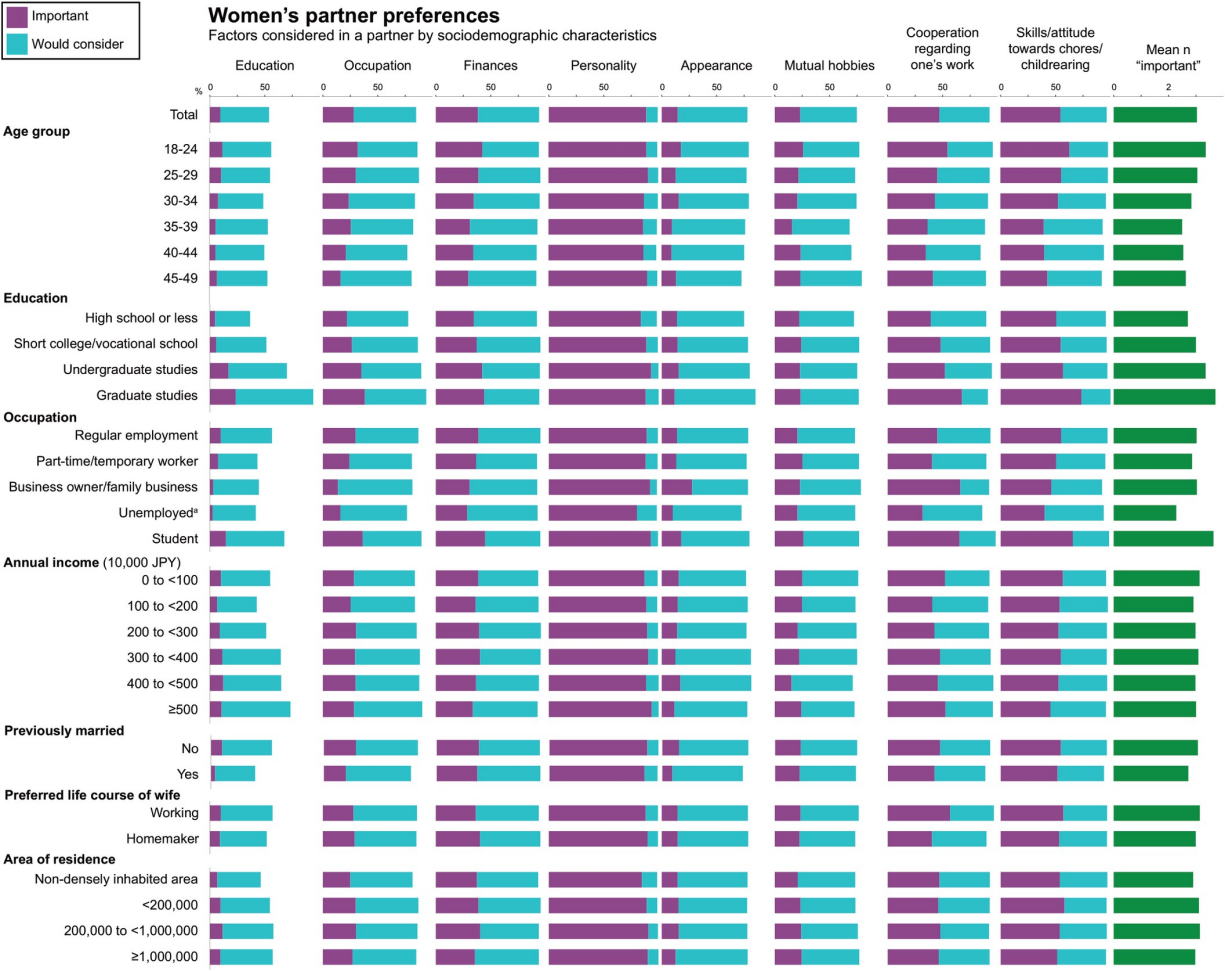
Factors listed as “important” or “would consider” in a partner among unmarried Japanese women with marriage intention, by sociodemographic characteristics.

**Fig 4:**
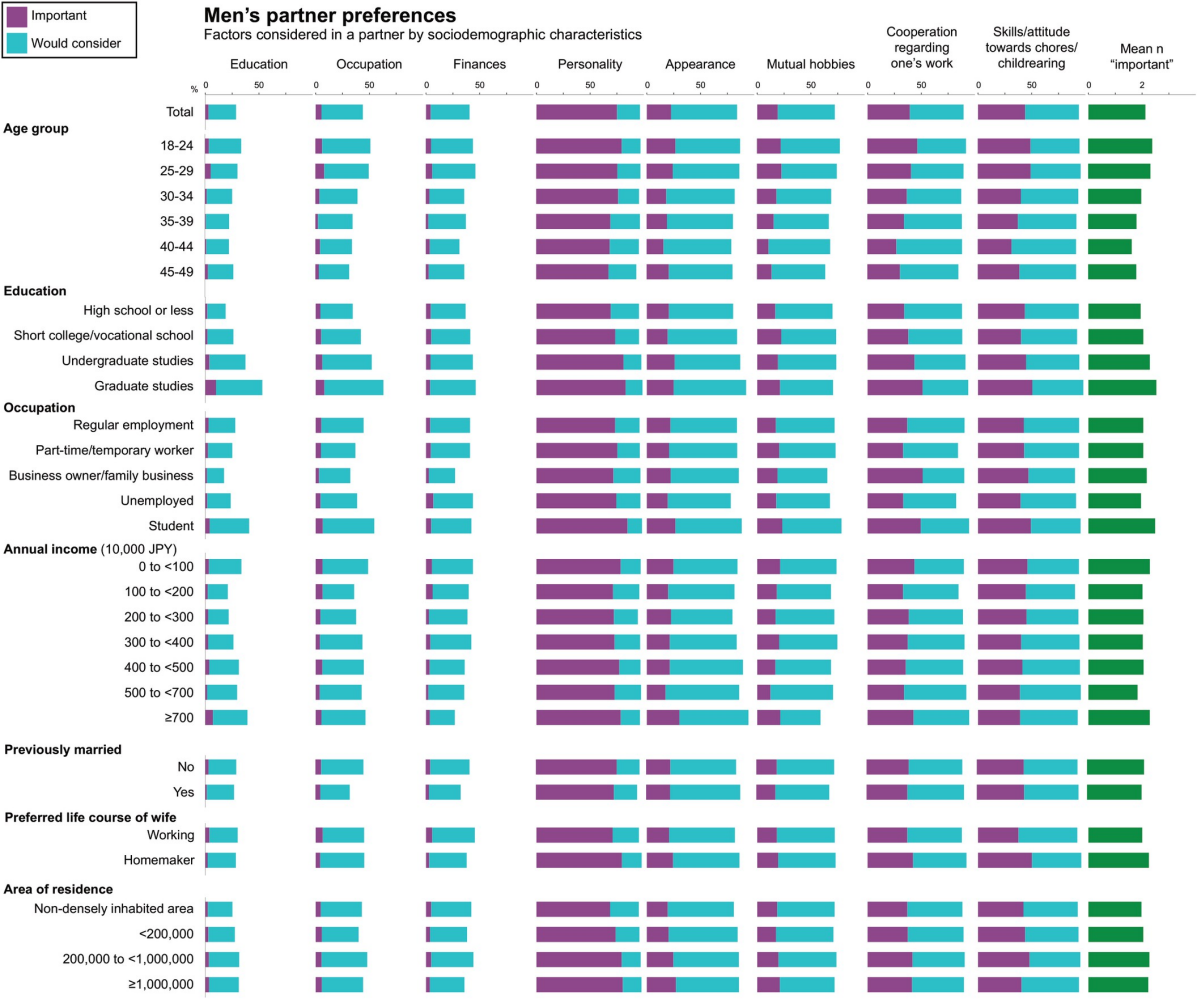
Factors listed as “important” or “would consider” in a partner among unmarried Japanese men with marriage intention, by sociodemographic characteristics.

Fig 3 (women) and Fig 4 (men) show the proportion of individuals listing an item as “important” and “would consider,” as well as the average number of items listed as “important” across sociodemographic characteristics (data for region in S15 and S16 Tables of S1 File). Items most commonly listed as “important” among both women and men were personality (women: 89%, men: 75%) and attitude towards and skills in housework and childrearing (54%, 44%). Women listed more items as “important” than men (average n of items listed as “important:” 3.0 vs. 2.1, p<0.0001), and a larger proportion of women vs. men responded “important” on all items except “appearance” (15% vs. 23%) (p-value for differences between women and men, p<0.0001 for all items). The largest differences between the genders in the proportion listing items as “important” were observed for finances (38% vs. 4%), occupation (28% vs. 5%), and personality (89% vs. 75%). The largest differences between the proportion of women and men listing items as “important” or “would consider” were observed for finances (94% vs. 41%, p<0.0001), occupation (85% vs. 44%, p<0.0001), and education (54% vs. 29%, p<0.0001).

For both women and men, the number of “important” items tended to be higher among those aged 18–34 years than those who were older. Moreover, among both women and men, the number of “important” items increased with higher education, a pattern largely driven by preferences regarding education, occupation, finances (women), and personality (men). Similarly, the proportion listing education as “important” or “would consider” increased with increasing income among women. Among women, those who were unemployed and those who were previously married listed fewer items as “important” while this did not vary considerably across income categories or by size and population density of area of residence. Among men, the number of items listed as “important” did not vary considerably by occupational status, income category, or history of marriage while those living in rural areas or small cities tended to list fewer items as “important.” These findings were largely consistent in analyses performed separately for those aged 18–24 years and 25–49 years (S17-S20 Tables of S1 File). Furthermore, analyses stratified by history of previous marriage showed relatively similar preferences between never- and previously-married men and women, with the following exceptions: previously-married women placed a decreased emphasis on the education and appearance of potential partners while previously-married men placed a decreased emphasis on the occupation of potential partners (S21-S24 Tables of S1 File).

### Ideal age of marriage and ideal age of the partner

**Fig 5:**
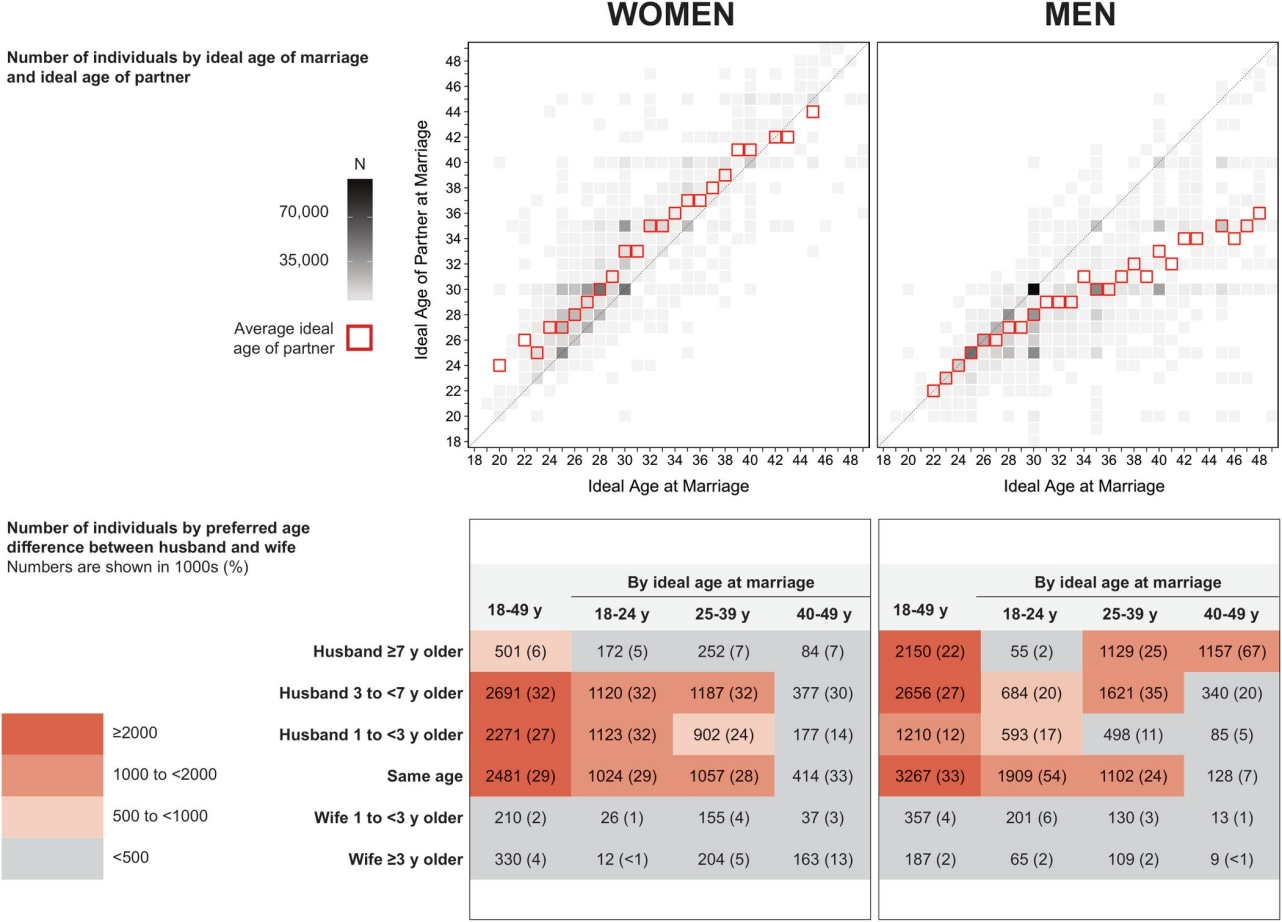
Ideal age of marriage and ideal age of partner among unmarried Japanese women and men, aged 18–49 years, with marriage intention.

The average ideal age at marriage and the ideal age of partners for unmarried women and men with marriage intention is shown in Fig 5. While most men and women indicated that they preferred that the husband would be the same age or older than the wife, men tended to prefer larger age differences between husband and wife. For example, while 2.15 million (22%) men indicated an ideal age difference of ≥7 years, the corresponding number for women was 501,000 (6%). Among men who wanted to marry at age 40–49 years, 1.16 million men indicated this preferred age difference; the corresponding number among the women who wanted to marry at age 25–39 years was only 252,000 (7%). Accordingly, among women, the average ideal age of the partner tended to be around 1–3 years older than their ideal age of marriage (Pearson correlation coefficient, 0.85, p<0.0001). However, among men, the average ideal age of the partner equalled their ideal age of marriage up until 26 years, at which point men tended to prefer women younger than themselves, with the preferred age difference increasing substantially with age: by the ideal age of marriage of 48 years, the average preferred partner age was 36 years among men (Pearson correlation coefficient, 0.70, p<0.0001). Age preferences were similar among never- and previously-married respondents and are shown in S1 Fig. Actual age at marriage and age of partner at marriage for married survey respondents in 2015 are shown in Tables 2 and 3 and S2 Fig. In 12% of the couples the husband was ≥7 years older than the wife, while in 66% of the couples the husband was the same age or less than 7 years older than the wife. Nonetheless, the age composition of married couples should not be used to assess the effect of gender differences in age preferences on the likelihood of marriage among unmarried individuals. In fact, age preferences of women and men largely overlap during the mid-20s to early 30s when most marriages occur, while the disparities among unmarried individuals emerge in older age groups. Moreover, it is likely that partner preferences are associated with the likelihood of being married, especially if such preferences conflict with those of potential partners. The age preferences of married individuals are also uncertain: it is possible that they did not marry at their preferred age with a partner of their preferred age.

**Table 2:** Age difference between wife and husband at the time of marriage among married couples in the National Fertility Survey 2015. Numbers are shown as n (%).

**Age of wife at marriage**	**18–49**	**18–24**	**25–39**	**40–49**
Husband ≥7 y older	783 (12.4)	292 (15.7)	473 (10.9)	18 (17.8)
Husband 3 to <7 y older	1538 (24.4)	547 (29.4)	980 (22.6)	11 (10.9)
Husband 1 to <3 y older	1459 (23.2)	463 (24.9)	979 (22.6)	17 (16.8)
Same age	1139 (18.1)	353 (19.0)	772 (17.8)	14 (13.9)
Wife 1 to <3 y older	875 (13.9)	174 (9.4)	683 (15.8)	18 (17.8)
Wife ≥3 y older	502 (8.0)	29 (1.6)	450 (10.4)	23 (22.8)
Total	6296 (100.0)	1858 (100.0)	4337 (100.0)	101 (100.0)
**Age of husband at marriage**	**18–49**	**18–24**	**25–39**	**40–49**
Husband ≥7 y older	739 (11.8)	0 (0)	559 (11.7)	180 (60.8)
Husband 3 to <7 y older	1538 (24.6)	70 (6.0)	1412 (29.5)	56 (18.9)
Husband 1 to <3 y older	1459 (23.3)	268 (23.0)	1159 (24.2)	32 (10.8)
Same age	1139 (18.2)	353 (30.3)	772 (16.1)	14 (4.7)
Wife 1 to <3 y older	875 (14.0)	294 (25.2)	569 (11.9)	12 (4.1)
Wife ≥3 y older	502 (8.0)	181 (15.5)	319 (6.7)	<5
Total	6252 (100.0)	1166 (100.0)	4790 (100.0)	296 (100.0)

**Table 3:** Age of wife and husband at the time of marriage among married couples in the National Fertility Survey 2015. Numbers are shown as n (% of the total number of married couples).

Age at marriage	Husband 18–24 years	Husband 25–39 years	Husband ≥40 years
**Wife 18–24 years**	894 (14.2)	945 (15.0)	19 (0.3)
**Wife 25–39 years**	272 (4.3)	3818 (60.6)	247 (3.9)
**Wife 40–49 years**	0 (0)	27 (0.4)	74 (1.2)

## Discussion

A large and increasing proportion of adults in Japan remains unmarried even though most of them report that they intend to get married. In this study, we used nationally representative survey data to examine factors associated with marital status and intention to marry, estimate the number and describe the characteristics of unmarried Japanese women and men in the marriage market, and assess their partner preferences.

We estimated that there were around 8.48 million unmarried women and 9.83 million unmarried men, aged 18–49 years, with marriage intention. While the number of women and men was similar among those aged 18–24 years, the surplus of men was around 800,000 (men-to-women ratio 1.22) among those aged 25–39 years and 500,000 (men-to-women ratio 1.38) among those aged 40–49 years. This was a result of the larger number of men in the population and the larger proportion of men who were unmarried (in part a result of more women than men being married to a partner older than 49 years). Moreover, although more women than men had been previously married, the surplus of men was exacerbated by the larger proportion of previously-married men than women who desire remarriage (S6 Table of S1 File). When analysed by area of residence, the surplus of men was particularly pronounced (around 600,000) in non-densely inhabited areas (men-to-women ratio 1.31) and in the Kanto region (men-to-women ratio 1.23), indicating that the geographical distribution of women and men may potentially constitute a barrier to union formation in the marriage market.

The purported preference for men with high income and education is captured by the term “*sankou*” (“three highs” [high income, high education, height]). *Sankou* is popularly used to describe desirable characteristics in a potential husband and was especially widespread during the economic bubble in the late 1980s and early 1990s; this preference may partly be due to difficulties in combining full-time work with childrearing and the notion that women are primarily responsible for the household. Our analyses, which did not account for height, show how few men are available in the marriage market that satisfy just two-thirds of these criteria. As previously reported [8, 16], the likelihood of being married was higher for men with higher income, regular employment, and higher education (for those aged 40–49 years). In fact, among men with an annual income of ≥7,000,000 JPY (≥66,000 USD), 84% (25–39 years) and 92% (40–49 years) were already married while the corresponding numbers for those with an annual income of 0 to <1,000,000 JPY (0 to <9,000 USD) was 23% (25–39 years) and 33.4% (40–49 years). Due to their larger number and the lower likelihood of being married, men with lower income constituted most of the men in the Japanese marriage market, even though the proportion with no intention to marry was also higher among these men. For example, there were 6.18 million men who had an annual income of 0 to <3,000,000 JPY (0 to <28,000 USD) (62%), as compared with 1.96 million (20%) men with an annual income of ≥4,000,000 JPY (≥38,000 USD), and only 167,000 (2%) men had an income of ≥7,000,000 JPY (≥66,000 USD), with roughly three-quarters of them possessing a university degree.

While popular notion suggests that Japanese women with high income and education may have difficulties finding a partner due to high expectations for potential husbands or due to men being intimidated by women with high socioeconomic status [9, 10], our analyses showed that the women who were the least likely to be married were those with annual incomes of 2,000,000 to <4,000,000 JPY (19,000 to <38,000 USD). Moreover, among unmarried women with marriage intention, women in lower income groups were far more numerous than those with higher incomes (e.g., there were only 256,000 such women with an annual income of ≥5,000,000 JPY [≥47,000USD]). Similarly, women without a university education were less likely to be married and outnumbered those with a university degree or more. Importantly, a previous study has shown that higher income (as measured before marriage) and education were associated with a higher likelihood of marriage among women [11]; therefore, higher income seems to be an advantage for both women and men in the marriage market. These data are also in accordance with those from other high-income countries showing that women with higher income and education are more likely to get married than those with lower income and education [13].

We observed gender differences in partner preferences in line with previous reports from the National Fertility Survey [3]. When asked about factors that one may consider in a potential partner, the proportion of women listing an item as “important” tended to be larger than those of men across all items (education, occupation, finances, personality, mutual hobbies, cooperation/understanding regarding one’s work, and attitude towards/skills in housework and childrearing) except appearance. Notably, a high proportion of the women listed “attitude towards/skills in housework and childrearing as “important” (54%) or “would consider (42%), reflecting an increasing expectation for men to contribute to domestic work. This preference may partly be captured in another term commonly used to describe desirable characteristics of a husband, *santei* (“three lows”), which refers to low risk (stable employment), low maintenance (is capable of taking care of himself and does not need help with chores), and humility (not believing that he has authority because he is a man).

The largest differences in preferences between the genders were observed for finances (proportion of women vs. men listing the item as “important” or “would consider:” 94% vs. 41%), occupation (85% vs. 44%), and education (54% vs. 29%). A large body of literature shows that women, when asked about their ideal partner preferences, tend to put more emphasis on a potential partner’s income and social status than men, and men tend to value physical attractiveness more than women [19]. In Japan, two surveys of 25-34-year old unmarried women from across the country found that 9 in 10 women preferred a partner with an income equal to or higher than their own, such that around 60% preferred the partner’s income to be at least 4,000,000 JPY (38,000 USD) [7, 15].

Accordingly, in the discourse on the Japanese marriage market and “*kon-katsu*” (spouse hunting), much attention is devoted to income, education, and occupational status–the so-called “specs” of potential partners. For example, profile pages in online partner search services typically highlight these attributes, and prominent matchmaking services frequently use these items as criteria for joining recreational events or as a requirement for membership (for men). Importantly, while the use of such heuristics may provide some information regarding the utility of the potential marriages, research has shown that what individuals list as ideal partner characteristics poorly predicts partner selection when potential partners are introduced in real-life [19]. Thus, the emphasis on partner “specs” could result in the premature dismissal of potentially compatible unions as spouse-selection criteria may preclude such encounters. This is important because, as shown in our study, the supply of unmarried men with higher income and education is scarce. Our estimates show that competition for high-income men would be fierce, especially for high-income women, if women in Japan were to prefer men with equal or higher income than themselves (homogamy/hypergamy). For example, the number of unmarried women with marriage intention who have an income of ≤5,000,000 JPY (≤47,000 USD) per man with marriage intention earning ≥5,000,000 JPY (≥47,000 USD) would be 9.49; this number would be 16.52 if limiting the men of interest to those with a university degree or higher. The corresponding numbers if considering men who earn ≥7,000,000 JPY (66,000 USD) would be 50.78 and 65.41, respectively. That deficits of men with employment, higher income and a college degree may constitute a reason for declines in marriage rates has been indicated in a US study which used socioeconomic characteristics of married men as a measure of unmarried women’s partner preferences [20]. While the increase in women with higher education across high-income countries has led to changes in partner preferences and union formation with respect to the educational level of husbands compared to wives [13], research suggests that women’s preference for men with higher income (regardless of their educational level) remains pronounced [12, 13].

We also note that among both women and men, the number of items considered as “important” in a potential partner tended to increase with level of education, with this being primarily driven by considerations of the partner’s education, occupation and finances. Interestingly, the number of “important” items did not vary considerably across occupational status (men), income category, size and population density of area of residence (women).

Our analyses of the ideal age of marriage and the ideal age of the partner identified a potential mismatch between supply and demand in the Japanese marriage market. On average, women preferred men who were 1–3 years older than themselves at the time of marriage. In contrast, men tended to prefer women who were around the same age until the age of 26 years (at the time of marriage); after this age, men tended to prefer women who were younger than themselves, with the preferred age difference increasing substantially with age. Accordingly, the number of men preferring a younger partner was larger than the number of women who preferred an older partner. For example, 1.16 million (67%) men who wanted to marry at age 40–49 years preferred a partner who is ≥7 years younger than themselves whereas only 252,000 (7%) women who wanted to marry at age 25–39 years preferred a man ≥7 years older than themselves. That many men tend to prefer women younger than themselves for romantic or sexual relationships is well-established [3, 21, 22], and in the context of the Japanese marriage market, this preference may also reflect a desire to increase the likelihood of having children. However, it has been shown that men consider a wider range of age groups as they grow older [22], and it should be noted that the age preferences assessed in our study represent an “ideal age” of the partner and not an age range that the respondent would consider.

The potential mismatches between the supply and demand in the Japanese marriage market that were identified in our study should be regarded as hypothesis-generating as we could not assess the impact of such mismatches on marriage rates. Individuals may calibrate their partner search criteria depending on what is available to them in the market and the identified mismatches may therefore not affect marriage behaviour. As such, individuals searching for a partner may find the data provided in our study useful for outlining partner search criteria. Our study has other limitations. First, due to the cross-sectional nature of our data, it was not possible to assess factors such as income before marriage. Longitudinal studies are needed for assessing the association between income and likelihood of marriage, especially for women [11], as many become homemakers or work shorter hours after marriage. Second, as the survey data used was self-reported, findings may have been affected by social desirability bias. Third, non-response might have introduced bias in our results (the response rate was 76.5% among unmarried individuals and 87.8% among married couples). Fourth, as the survey of married individuals was designed to provide a nationally representative sample of married women, the sample of married men does not include those aged 49 years or younger whose wives were ≥50 years. The influence of this limitation is likely small as such couples constitutes only a low proportion of the married Japanese population [23]. Fifth, individuals could only choose from pre-specified items when indicating factors considered as important in a potential partner. Although the list included most items used for evaluating partner preferences in previous literature [13, 19, 24, 25], all factors of importance may not have been captured in the survey [26]. Moreover, participants were not asked to rank the different items, nor could they specify their partner preferences, for example, with respect to level of income or education. Finally, it should be noted that the data regarding factors considered when choosing a partner as well as age preferences are those reported by currently unmarried individuals and do not represent the overall Japanese population (including those who are married), in particular as spouse-selection criteria are likely to affect the likelihood of being married. However, the data for unmarried individuals is the most relevant for the study’s aim: namely, to describe the marriage market for those who are currently unmarried.

## Conclusions

In the Japanese marriage market, there were 8.48 million unmarried women and 9.83 million unmarried men, aged 18–49 years, with an intention to marry during their lifetime. Surpluses of men were most pronounced in non-densely inhabited areas and in the Kanto region. Most women, but not men, reported that they would consider the finances, occupation, and education of a potential partner. Few men with high income and education were available in the marriage market. A larger number of men preferred women who were younger than themselves than there were women who preferred older men.

## Supporting information

S1 File Additional methods and data. (DOCX)

## References

[1] Ministry of Health, Labour and Welfare. Statistics and white papers. https://www.mhlw.go.jp/toukei_hakusho/toukei/ (accessed 13 Aug 2019).

[2] Ministry of Internal Affairs and Communications. Statistics Japan. Statistics Bureau. Population Census. 2015.http://www.stat.go.jp/english/data/kokusei/index.html (accessed 25 Jun 2018).

[3] National Institute of Population and Social Security Research. The Fifteenth Japanese National Fertility Survey in 2015. Marriage Process and Fertility of Married Couples Attitudes toward Marriage and Family among Japanese Singles. Summary of the Survey Results on Married Couples/Singles. 2017. http://www.ipss.go.jp/ps-doukou/j/doukou15/doukou15_gaiyo.asp (accessed 22 Jun 2018).

[4] Raymo JM, Iwasawa M. Marriage Market Mismatches in Japan: An Alternative View of the Relationship between Women’s Education and Marriage. Am Sociol Rev 2005;70:801–22. doi: 10.1177/000312240507000504

[5] Blossfeld H-P, Timm A. Who Marries Whom? Educational Systems as Marriage Markets in Modern Societies: a Conceptual Framework. Dordrecht: Springer Netherlands 2003. doi: 10.1007/978-94-007-1065-8

[6] Zentner M, Eagly AH. A sociocultural framework for understanding partner preferences of women and men: Integration of concepts and evidence. Eur Rev Soc Psychol 2015;26:328–73. doi: 10.1080/10463283.2015.1111599

[7] Meiji Yasuda Daily Life and Welfare Research Institution. Attitudes and circumstances regarding marriage among those aged 25–34 years. From the survey on marriage and relationships between men and women. 2017. https://www.myilw.co.jp/research/report/pdf/myilw_report_2017_02.pdf (accessed 13 Aug 2019).

[8] The Japan Institute for Labour Policy and Training. Current state of professional development, career and employment status among young adults. From the 2017 Employment Status Survey. 2019. https://www.jil.go.jp/institute/siryo/2019/documents/217_00.pdf (accessed 13 Aug 2019).

[9] Sakai J. Make-inu no toboe (defeated dog’s cry). Kodansha bunko 2006.

[10] Arakawa Kazuhisa. Josei ga chokumen suru kasegu hodo kekkon dekinai genjitu [The reality women face—the more they earn the lower the likelihood of getting married]. Toyo Keizai Online. 2017.https://toyokeizai.net/articles/-/175446 (accessed 13 Aug 2019).

[11] Fukuda S. Shifting Economic Foundation of Marriage in Japan: The Erosion of Traditional Marriage. Max Planck Inst Demogr Res Published Online First: 2009.https://pdfs.semanticscholar.org/45aa/42603e829d6e15b3ffd9f0853e7a94c92b18.pdf (accessed 13 Aug 2019).

[12] Chudnovskaya M, Kashyap R. Is the end of educational hypergamy the end of hypergamy? Evidence from Sweden. Stockholm Reports in Demography. Stockholm University. 2017. https://figshare.com/articles/Is_the_end_of_educational_hypergamy_the_end_of_hypergamy_Evidence_from_Sweden_/5531824/1 (accessed 23 Jan 2019).

[13] Van Bavel J, Schwartz CR, Esteve A. The Reversal of the Gender Gap in Education and Its Consequences for Family Life. Annu Rev Sociol 2018;44:341–60. doi: 10.1146/annurev-soc-073117-041215

[14] Fukuda S, Yoda S, Mogi R. Three Decades of Educational Assortative Mating in Japan: A Micro-Data Analysis of Population Census 1980–2010. IPSS Work Pap Ser 2017;:1–9.

[15] Digital A shimbun. Unmarried men and women. Influence of the income barrier on desire to marry. Online survey. 2019.https://www.asahi.com/articles/photo/AS20190110002302.html (accessed 23 Aug 2019).

[16] Ministry of Health Labour and Welfare; Japan. The 6th Longitudinal Survey of Adults in the 21st Century. Published Online First: 2009.http://www.mhlw.go.jp/toukei/index.html (accessed 28 Jun 2018).

[17] Ministry of Health Labour and Welfare; Japan. 出生動向基本調査 [National Fertility Survey]. https://www.mhlw.go.jp/toukei/list/118-1.html (accessed 3 Jan 2019).

[18] Esteve A, Schwartz CR, van Bavel J, et al. The End of Hypergamy: Global Trends and Implications. Popul Dev Rev 2016;42:615–25. doi: 10.1111/padr.12012 28490820 PMC5421994

[19] Eastwick PW, Luchies LB, Finkel EJ, et al. The predictive validity of ideal partner preferences: A review and meta-analysis. Psychol Bull 2014;140:623–65. doi: 10.1037/a0032432 23586697

[20] Lichter DT, Price JP, Swigert JM. Mismatches in the Marriage Market. J Marriage Fam 2020;82:796–809. doi: 10.1111/jomf.12603

[21] Kenrick DT, Keefe RC. Age preferences in mates reflect sex differences in human reproductive strategies. Behav Brain Sci 1992;15:75–91. doi: 10.1017/S0140525X00067595

[22] Antfolk J. Age limits: Men’s and women’s youngest and oldest considered and actual sex partners. Evol Psychol 2017;15:147470491769040. doi: 10.1177/1474704917690401 28127998 PMC10367477

[23] The Ministry of Health, Labour and Welfare, Japan. Comprehensive Survey of Living Conditions. 2017. https://www.mhlw.go.jp/english/database/db-hss/cslc.html (accessed 7 Dec 2019).

[24] Buss DM, Shackelford TK, Kirkpatrick LA, et al. A Half Century of Mate Preferences: The Cultural Evolution of Values. J Marriage Fam 2001;63:491–503. doi: 10.1111/j.1741-3737.2001.00491.x

[25] Press JE. Cute butts and housework: A gynocentric theory of assortative mating. J Marriage Fam 2004;66:1029–33. doi: 10.1111/j.0022-2445.2004.00074.x

[26] Fletcher GJO, Thomas G, Giles L, et al. Ideals in intimate relationships. J Pers Soc Psychol 1999;76:72–89. doi: 10.1037//0022-3514.76.1.72 9972554

