# Supplementary material for: Fish in the sea: Number, characteristics, and partner preferences of unmarried Japanese adults - analysis of a national survey
Source: PLoS One. 2022 Feb 2;17(2):e0262528. doi: 10.1371/journal.pone.0262528 (PMC8809582; doi:10.1371/journal.pone.0262528)
Supplement: S1 File — (DOCX) [file pone.0262528.s001.docx]

**File S1: Supplementary Material**

Ghaznavi C, Sakamoto H, Nomura S, Yoneoka D, Kubota A, Shibuya K, Ueda P*. Fish in the sea: number, characteristics, and partner preferences of unmarried Japanese adults - analysis of a national survey.*

|  | **Page** |
| --- | --- |
| Survey questions used in analyses | 4 |
| Calculation of sample weights and extrapolation to the Japanese population | 6 |
| **STable 1** Definitions and categorization of socioeconomic and regional variables used for assessing marital status and marriage intention across population characteristics of participants in the National Fertility Survey 2015. | |
| **STable 2** Definitions and categorization of socioeconomic and regional variables used for describing characteristics of unmarried individuals with marriage intention and their partner preferences across population characteristics of participants in the National Fertility Survey 2015. | |
| **STable 3.** Definitions and categorization of socioeconomic and regional variables used for describing characteristics of unmarried individuals with marriage intention and their partner preferences across population characteristics of participants in the National Fertility Survey 2015. | |
| **STable 4.** Proportion of women who are unmarried with no marriage intention, unmarried with marriage intention, and married by age group and sociodemographic characteristics. | |
| **STable 5.** Proportion of men who are unmarried with no marriage intention, unmarried with marriage intention and married by age group and sociodemographic characteristics. | |
| **STable 6.** Proportion of women who are unmarried with marriage intention by sociodemographic characteristics, stratified by presence or absence of previous marriage. | |
| **STable 7.** Proportion of men who are unmarried with marriage intention by sociodemographic characteristics, stratified by presence or absence of previous marriage. | |
| **STable 8.** Proportion of women aged 18-24 years who are married, never-married with marriage intention, never-married without marriage intention, previously married with marriage intention and previously married without marriage intention by sociodemographic characteristics. | |
| **STable 9.** Proportion of women aged 25-39 years who are married, never-married with marriage intention, never-married without marriage intention, previously married with marriage intention and previously married without marriage intention by sociodemographic characteristics. | |
| **Stable 10.** Proportion of women aged 40-49 years who are married, never-married with marriage intention, never-married without marriage intention, previously married with marriage intention and previously married without marriage intention by sociodemographic characteristics. | |
| **STable 11.** Proportion of men aged 18-24 years who are married, never-married with marriage intention, never-married without marriage intention, previously married with marriage intention and previously married without marriage intention by sociodemographic characteristics. | |
| **STable 12.** Proportion of men aged 25-39 years who are married, never-married with marriage intention, never-married without marriage intention, previously married with marriage intention and previously married without marriage intention by sociodemographic characteristics. | |
| **STable 13.** Proportion of men aged 40-49 years who are married, never-married with marriage intention, never-married without marriage intention, previously married with marriage intention and previously married without marriage intention by sociodemographic characteristics. | |
| **STable 14.** Number of unmarried women and men aged 25-49 years, with marriage intention by income cut-off and number of potential partners available for hypergamy or homogamy. | |
| **STable 15.** Factors listed as “important” or “would consider” when choosing a partner among unmarried women with marriage intention in the National Fertility Survey 2015 by sociodemographic variables. | |
| **STable 16.** Factors listed as “important” or “would consider” when choosing a partner among unmarried men with marriage intention in the National Fertility Survey 2015 by sociodemographic variables. | |
| **STable 17.** Factors listed as “important” or “would consider” when choosing a partner among unmarried women with marriage intention, aged 18-24 years, in the National Fertility Survey 2015 by sociodemographic variables. | |
| **STable 18.** Factors listed as “important” or “would consider” when choosing a partner among unmarried men with marriage intention, aged 18-24 years, in the National Fertility Survey 2015 by sociodemographic variables. | |
| **STable 19.** Factors listed as “important” or “would consider” when choosing a partner among unmarried women with marriage intention, aged 25-49 years, in the National Fertility Survey 2015 by sociodemographic variables. | |
| **STable 20.** Factors listed as “important” or “would consider” when choosing a partner among unmarried men with marriage intention, aged 25-49 years, in the National Fertility Survey 2015 by sociodemographic variables. | |
| **STable 21.** Factors listed as “important” or “would consider” when choosing a partner among never-married women with marriage intention, aged 18-49 years, in the National Fertility Survey 2015 by sociodemographic variables. | |
| **STable 22.** Factors listed as “important” or “would consider” when choosing a partner among never-married men with marriage intention, aged 18-49 years, in the National Fertility Survey 2015 by sociodemographic variables. | |
| **STable 23.** Factors listed as “important” or “would consider” when choosing a partner among previously-married women with marriage intention, aged 18-49 years, in the National Fertility Survey 2015 by sociodemographic variables. Numbers are shown in percent. | |
| **STable 24.** Factors listed as “important” or “would consider” when choosing a partner among previously-married men with marriage intention, aged 18-49 years, in the National Fertility Survey 2015 by sociodemographic variables. | |
| **SFigure 1.** Ideal age of marriage and ideal age of partner among unmarried Japanese women and men, aged 18-49 years, with marriage intention and stratified by presence and absence of previous marriage. | |
| **SFigure 2.** Actual age of marriage and actual age of partner at marriage among married Japanese women and men, aged 18-49 years at time of marriage. The color of each box indicates the number of couples in the National Fertility Survey. | |
| **References** | |

**Survey questions used in analyses**

*Intention to marry*

“When thinking about your entire lifetime, which of the following represents your thoughts regarding marriage”

1. I intend to get married at some point

2. I intend to never get married during my lifetime

*Ideal age of marriage and ideal age of partner*

“At around what age do you wish to get married and around what age should your partner be. Please fill in the desired ages in the blanks below.”

- When I am around (___) years, I want to get married to a partner who is around (___) years.

*Factors considered in a potential partner*

“When you choose a partner for marriage, to what extent do you consider the items 1-8 below. Select one response for each item.”

| 1. The partner’s education | 1. Important | 2. Would consider | 3. Doesn’t matter |
| --- | --- | --- | --- |
| 2. The partner’s occupation | 1. Important | 2. Would consider | 3. Doesn’t matter |
| 3. The partner’s income and other financial resources | 1. Important | 2. Would consider | 3. Doesn’t matter |
| 4. The partner’s personality | 1. Important | 2. Would consider | 3. Doesn’t matter |
| 5. The partner’s appearance | 1. Important | 2. Would consider | 3. Doesn’t matter |
| 6. Whether you have mutual interests or not | 1. Important | 2. Would consider | 3. Doesn’t matter |
| 7. The partner’s understanding and cooperation regarding my own work | 1. Important | 2. Would consider | 3. Doesn’t matter |
| 8. Skills in and attitude towards housework and childrearing | 1. Important | 2. Would consider | 3. Doesn’t matter |

*Preferred life course of wife after marriage*

To women: Which type of life course would you consider as ideal?

To men: Which type of life course would you like the woman who will be your partner (or wife) to have?

[Survey participants were asked to choose one of the following options]:

1. Not getting married and continue working.

2. Getting married but not have children and continue working.

3. Getting married, have children, but also continue working.

4. Getting married, have children, quit working during the period around childbirth, and go back to work after childrearing is done.

5. Getting married, have children, quit working during the period around childbirth, without going back to work.

6. Other (______)

**Variable names for variable used in the analyses (as presented in the National Fertility Survey 2015 dataset)**

| Unmarried survey | Married survey |
| --- | --- |
| Q1A, Q1B, Q1C, Q1D, Q3A, Q3B, Q3B1, Q3B2, Q4A1, Q4A2, Q4B, Q4D, Q4E, Q9A, Q9B, Q9C, Q12A, Q22, Q23, Q25A, Q25B, Q26_1, Q26_2  Q26_3, Q26_4, Q26_5, Q26_6, Q26_7, Q26_8, Chiiki7Grp, DID7Grp, CHIKU | Q1A, Q1B, Q1C, Q1D, Q1E, Q1F, Q2A, Q2B, Q2D, Q2E, Q4D1, Q4D2, Q4G1, Q4G2, Q5D1, Q5E1, Q5D2, Q5E2, Chiiki7Grp, DID7Grp, CHIKU |

**Calculation of sample weights and extrapolation of findings to the Japanese population**

We used data from the Population Census to obtain information about the number of individuals by sex, age, and marital status (never-married, married, divorced, and widowed) and calculated sample weights which accounted for non-response and unknown marriage intention with respect to sex, age, and marital status. Sample weights, which were defined as the inverse of the probability of being sampled according to age (1-year increments) and marital status in the Population Census of Japan, were calculated separately for men and women by using population weights and survey weights. Due to low numbers, married individuals between the ages 18 and 22 years were considered as one group in the calculation of sample weights. For each sex, the population weight for any one age and marital status group was calculated as the proportion of the total number of adults (of the same sex), aged 18-49 years, in the census who belonged to that group. Survey weights were calculated as the proportion of the total number of 18-49-year-old survey participants, by sex, within the age and marital status group, after exclusion of participants with unknown marriage intention. Marital status was defined as either married or unmarried (including never-married, divorced and widowed), classified by the sub-survey to which the individual was responding.

Analyses accounted for the stratified cluster sampling in the calculation of standard errors. We used the sample weights to estimate the proportion of Japanese women and men who were married, unmarried with marriage intention, and unmarried with no such intention across sociodemographic variables. The extrapolation to the Japanese population was done by multiplying the estimated proportion with the total number of Japanese women and men, respectively. For analyses limited to those who were unmarried, we did not use sample weights as the sub-survey of unmarried individuals is nationally representative without weighting.^1^

**STable 1.** Characteristics of unmarried participants who were excluded vs. included in the analyses. Numbers are shown in n (%).

|  | **Men** | | | **Women** | | |
| --- | --- | --- | --- | --- | --- | --- |
|  | Excluded (n=331 [7.4%]) | Included (n=4168 [92.6%]) | p | Excluded (n=310 [7.3%]) | Included (n=3941 [92.7%]) | p |
| ***Education*** |  |  | <0.001 |  |  | <0.001 |
| High school or less | 165 (54) | 1749 (43) |  | 137 (47) | 1337 (34) |  |
| Vocational school/short college | 54 (18) | 751 (18) |  | 85 (29) | 1280 (33) |  |
| Undergraduate studies | 83 (27) | 1423 (35) |  | 65 (22) | 1230 (32) |  |
| Graduate studies | <5 | 187 (5) |  | <5 | 53 (1) |  |
| ***Occupational Status*** |  |  | 0.342 |  |  | 0.085 |
| Regular employee | 112 (46) | 2068 (52) |  | 102 (41) | 1750 (46) |  |
| Part-time/temporary worker | 41 (17) | 597 (15) |  | 80 (32) | 1020 (27) |  |
| Business owner/family business | 14 (6) | 238 (6) |  | 6 (2) | 102 (3) |  |
| Unemployed | 28 (11) | 400 (10) |  | 31 (12) | 339 (9) |  |
| Student | 49 (20) | 651 (16) |  | 31 (12) | 581 (15) |  |
| ***Annual Income (in JPY 10,000s)*** |  |  | <0.001 |  |  | <0.001 |
| 0-99 | 183 (64) | 1578 (39) |  | 145 (55) | 1471 (38) |  |
| 100-199 | 17 (6) | 344 (8) |  | 42 (16) | 722 (19) |  |
| 200-299 | 25 (9) | 682 (17) |  | 40 (15) | 859 (22) |  |
| 300-399a | 26 (9) | 672 (16) |  | 23 (9) | 492 (13) |  |
| 400-499 | 18 (6) | 429 (11) |  | 7 (3) | 167 (4) |  |
| 500-699 | 12 (4) | 302 (7) |  | 9 (3) | 137 (4) |  |
| ≥700 | <5 | 67 (2) |  | - | - |  |
| ***Region of Residence*** |  |  | <0.001 |  |  | <0.001 |
| Hokkaido | 12 (4) | 132 (3) |  | 18 (6) | 136 (3) |  |
| Tohoku | 27 (8) | 260 (6) |  | 23 (7) | 309 (8) |  |
| Kanto | 82 (25) | 1546 (37) |  | 91 (29) | 1339 (34) |  |
| Chubu | 75 (23) | 781 (19) |  | 62 (20) | 711 (18) |  |
| Kinki | 57 (17) | 660 (16) |  | 58 (19) | 690 (18) |  |
| Chugoku/Shikoku | 25 (8) | 385 (9) |  | 31 (10) | 309 (8) |  |
| Kyushu/Okinawa | 53 (16) | 404 (10) |  | 27 (9) | 447 (11) |  |
| ***Area of Residence:  Population Size and Density*** |  |  | <0.001 |  |  | <0.001 |
| Non-densely inhabited area | 135 (41) | 1224 (29) |  | 88 (28) | 1063 (27) |  |
| <200,000 | 68 (21) | 982 (24) |  | 72 (23) | 933 (24) |  |
| 200,000 to <1,000,000 | 80 (24) | 1153 (28) |  | 97 (31) | 1151 (29) |  |
| ≥1,000,000 | 48 (15) | 809 (19) |  | 53 (17) | 794 (20) |  |

Missing values were: education (men [n=84]; women [n=61]); occupational status (men [n=301]; women [n=209]); annual income (men [n=140]; women [n=137]).

**STable 2.** Definitions and categorization of socioeconomic and regional variables used for assessing marital status and marriage intention across population characteristics of participants in the National Fertility Survey 2015.

| *Variable* | *Categorization* | *Missing, weighted %* |
| --- | --- | --- |
| Education | High school or less; vocational school or short-term college; undergraduate studies; graduate studies. | 0.9 |
| Occupational status | Regular employee; part-time or temporary worker; business owner or member of family business; unemployed^a^; student^a^ | 3.9 |
| Annual income in 10,000 Japanese Yen (JPY)^b, c^ | *Men 25-39 y and 40-49 y:*  0 to <100; 100 to <200; 200 to <300; 300 <400; 400 to <500; 500 to <700; ≥700  *Women and men 18-24 y:*  0 to <100; 100 to <200; 200 to <300; ≥300  *Women 25-39 y and 40-49 y:*  0 to <100; 100 to <200; 200 to <300; 300 <400; 400 to <500; ≥500 | 3.3 |
| Region of residence^d^ | Hokkaido; Tohoku; Kanto; Chubu; Kinki; Chugoku/Shikoku; Kyushu/Okinawa | 0 |
| Population density and size of residence | Non-densely inhabited district; district with less than 200,000 inhabitants; between 200,000 and 1,000,000 inhabitants; more than 1,000,000 inhabitants^2^ | 0 |

a. The category “unemployed” also included students among those aged 40-49 years.

b. 10,000 JPY was approximately 94.7 USD as of October 2020. Income was categorized according to the individual’s revenue.

c. Different categorizations depending on age and gender in order to increase statistical power.

d. The seven regions constitute geographically clustered prefectures (the highest administrative divisions of Japan) and are often used in discussion of regional economic and policy issues in the country.

**STable 3.** Definitions and categorization of socioeconomic and regional variables used for describing characteristics of unmarried individuals with marriage intention and their partner preferences across population characteristics of participants in the National Fertility Survey 2015.

| *Variable* | *Categorization* | *Missing,*  *%* |
| --- | --- | --- |
| Education | High school or less; vocational school or short-term college; undergraduate studies; graduate studies. | 1.1 |
| Occupational status | Regular employee; part-time or temporary worker; business owner or member of family business; unemployed^a^; student^a^ | 4.1 |
| Annual income in 10,000 Japanese Yen (JPY)^b, c^ | *Men 25-39 y and 40-49 y:*  0 to <100; 100 to <200; 200 to <300; 300 <400; 400 to <500; 500 to <700; ≥700  *Women and men 18-24 y:*  0 to <100; 100 to <200; 200 to <300; ≥300  *Women 25-39 y and 40-49 y:*  0 to <100; 100 to <200; 200 to <300; 300 <400; 400 to <500; ≥500 | 2.3 |
| Previously married | No; yes | 1.3 |
| Desired life course of wife after marriage | Working^d^; Homemaker^e^ | 7.0 |
| Region of residence^f^ | Hokkaido; Tohoku; Kanto; Chubu; Kinki; Chugoku/Shikoku; Kyushu/Okinawa | 0 |
| Population density and size of residence | Non-densely inhabited district; district with less than 200,000 inhabitants; between 200,000 and 1,000,000 inhabitants; more than 1,000,000 inhabitants^2^ | 0 |
| Ideal age at marriage and ideal age of partner; age difference (husband-wife) | ≥7 y older; 3 to <7 y older; 1 to <3 y older: same age; 1 to <3 y younger; ≥3 y younger | 16.9 |

a. The category “unemployed” also included students among those aged 40-49 years.

b. 10,000 JPY was approximately 93.1 USD as of July 2020. Income was categorized according to the individual’s revenue.

c. Different categorizations depending on age and gender in order to increase statistical power.

d. Answers 1-3 to the question regarding the ideal/preferred life course of wife after marriage as described above.

e. Answers 4-5 to the question regarding the ideal/preferred life course of wife after marriage as described above.

f. The seven regions constitute geographically clustered prefectures (the highest administrative divisions of Japan) and are often used in discussion of regional economic and policy issues in the country.

**STable 4.** Proportion of women who are unmarried with no marriage intention, unmarried with marriage intention, and married by age group and sociodemographic characteristics. Numbers are shown in percent.

|  | 18-24 y | | | | 25-39 y | | | | 40-49 y | | | | 18-49 y | | | |
| --- | --- | --- | --- | --- | --- | --- | --- | --- | --- | --- | --- | --- | --- | --- | --- | --- |
|  | No marriage  intention | Marriage  intention | Married | p | No marriage  intention | Marriage  intention | Married | p | No marriage  intention | Marriage  intention | Married | p | No marriage  intention | Marriage  intention | Married | p |
| Total | 6.0 | 88.2 | 5.8 |  | 6.8 | 36.2 | 57.0 |  | 12.7 | 15.2 | 72.1 |  | 9.0 | 36.9 | 54.2 |  |
| ***Education*** |  |  |  | < 0.0001 |  |  |  | < 0.0001 |  |  |  | 0.0022 |  |  |  | < 0.0001 |
| High school or less | 6.4 | 79.9 | 13.7 |  | 8.9 | 32.8 | 58.3 |  | 15.4 | 15.3 | 69.3 |  | 11.6 | 31.4 | 57.0 |  |
| Vocational school/  short college | 5.7 | 90.2 | 4.1 |  | 6.0 | 34.7 | 59.3 |  | 10.8 | 15.3 | 73.9 |  | 7.9 | 33.2 | 58.9 |  |
| Undergraduate studies | 5.8 | 93.3 | 1.0 |  | 4.9 | 42.9 | 52.2 |  | 9.0 | 14.1 | 76.9 |  | 6.1 | 50.5 | 43.4 |  |
| Graduate studies | 12.9 | 87.1 | 0 |  | 13.1 | 27.7 | 59.2 |  | 16.2 | 19.4 | 64.4 |  | 13.9 | 28.6 | 57.6 |  |
| ***Occupational Status*** |  |  |  | < 0.0001 |  |  |  | < 0.0001 |  |  |  | < 0.0001 |  |  |  | < 0.0001 |
| Regular employee | 2.6 | 94.1 | 3.3 |  | 7.0 | 54.0 | 39.0 |  | 18.0 | 25.2 | 56.8 |  | 9.8 | 51.3 | 38.9 |  |
| Part-time/temporary worker | 8.4 | 80.3 | 11.3 |  | 8.4 | 33.8 | 57.8 |  | 11.8 | 11.6 | 76.6 |  | 10.1 | 26.7 | 63.2 |  |
| Business owner/ family business | 16.6 | 38.8 | 44.7 |  | 8.0 | 24.6 | 67.4 |  | 11.6 | 11.3 | 77.1 |  | 10.2 | 17.9 | 71.9 |  |
| Unemployed | 12.0 | 54.1 | 33.9 |  | 3.7 | 13.2 | 83.1 |  | 8.1 | 9.2 | 82.6 |  | 6.0 | 13.4 | 80.6 |  |
| Student | 6.4 | 93.4 | 0.2 |  | 4.8 | 15.9 | 79.3 |  | 6.9 | 8.3 | 84.8 |  | 6.4 | 92.5 | 1.1 |  |
| ***Annual Income  (in JPY 10,000s)*** |  |  |  | 0.1414 |  |  |  | < 0.0001 |  |  |  | < 0.0001 |  |  |  | < 0.0001 |
| 0-99 | 6.9 | 87.1 | 6.0 |  | 4.8 | 15.9 | 79.3 |  | 6.9 | 8.3 | 84.8 |  | 6.1 | 29.8 | 64.1 |  |
| 100-199 | 5.8 | 88.9 | 5.3 |  | 11.5 | 43.8 | 44.6 |  | 15.2 | 15.2 | 69.7 |  | 12.5 | 36.4 | 51.2 |  |
| 200-299 | 2.2 | 93.4 | 4.4 |  | 7.5 | 60.7 | 31.8 |  | 24.8 | 25.3 | 49.8 |  | 12.1 | 54.8 | 33.1 |  |
| 300-399^a^ | 1.3 | 91.7 | 7.0 |  | 6.8 | 59.2 | 34.0 |  | 19.8 | 29.2 | 51.0 |  | 10.5 | 52.4 | 37.1 |  |
| 400-499 | - | - | - |  | 6.2 | 46.2 | 47.7 |  | 15.9 | 22.0 | 62.1 |  | 10.1 | 36.6 | 53.4 |  |
| ≥500 | - | - | - |  | 8.5 | 35.8 | 55.7 |  | 15.1 | 24.6 | 60.4 |  | 12.7 | 29.0 | 58.3 |  |
| ***Region of Residence*** |  |  |  | 0.038 |  |  |  | 0.0398 |  |  |  | 0.055 |  |  |  | < 0.0001 |
| Hokkaido | 2.6 | 86.9 | 10.5 |  | 10.3 | 35.7 | 54.0 |  | 17.9 | 14.7 | 67.4 |  | 12.0 | 35.7 | 52.3 |  |
| Tohoku | 7.4 | 81.9 | 10.8 |  | 8.6 | 42.2 | 49.1 |  | 16.0 | 16.3 | 67.7 |  | 11.1 | 39.3 | 49.6 |  |
| Kanto | 5.4 | 90.7 | 3.8 |  | 6.3 | 36.7 | 57.0 |  | 10.8 | 15.0 | 74.3 |  | 7.9 | 38.5 | 53.7 |  |
| Chubu | 7.1 | 88.0 | 4.9 |  | 6.1 | 33.9 | 60.0 |  | 12.0 | 12.4 | 75.7 |  | 8.6 | 33.5 | 57.9 |  |
| Kinki | 5.8 | 89.0 | 5.2 |  | 6.4 | 38.5 | 55.1 |  | 13.3 | 17.8 | 68.9 |  | 9.1 | 39.0 | 51.9 |  |
| Chugoku/Shikoku | 10.4 | 77.5 | 12.1 |  | 6.7 | 30.3 | 63.0 |  | 15.8 | 13.7 | 70.5 |  | 10.4 | 31.2 | 58.4 |  |
| Kyushu/Okinawa | 3.8 | 89.7 | 6.4 |  | 7.8 | 36.7 | 55.6 |  | 13.2 | 17.4 | 69.4 |  | 9.2 | 38.0 | 52.8 |  |
|  |  |  |  |  |  |  |  |  |  |  |  |  |  |  |  |  |
| ***Area of Residence:  Population*** *Size* and Density |  |  |  | 0.179 |  |  |  | 0.3579 |  |  |  | 0.2145 |  |  |  | 0.5092 |
| Non-densely inhabited area | 7.1 | 84.8 | 8.0 |  | 7.1 | 35.7 | 57.2 |  | 13.8 | 13.0 | 73.2 |  | 9.6 | 35.4 | 54.9 |  |
| <200,000 | 4.5 | 89.7 | 5.8 |  | 7.5 | 34.1 | 58.4 |  | 11.7 | 14.7 | 73.7 |  | 8.5 | 36.7 | 54.7 |  |
| 200,000 to <1,000,000 | 7.1 | 87.9 | 4.9 |  | 7.0 | 36.9 | 56.1 |  | 11.8 | 16.5 | 71.7 |  | 8.9 | 37.5 | 53.6 |  |
| ≥1,000,000 | 4.7 | 91.3 | 4.0 |  | 5.3 | 38.3 | 56.5 |  | 13.7 | 16.8 | 69.5 |  | 8.5 | 38.0 | 53.4 |  |

a. ≥300 for those aged 18-24 years.

**STable 5.** Proportion of men who are unmarried with no marriage intention, unmarried with marriage intention and married by age group and sociodemographic characteristics. Numbers are shown in percent.

|  | 18-24 y | | | | 25-39 y | | | | 40-49 y | | | | 18-49 y | | | |
| --- | --- | --- | --- | --- | --- | --- | --- | --- | --- | --- | --- | --- | --- | --- | --- | --- |
|  | No marriage  intention | Marriage  intention | Married | p | No marriage  intention | Marriage  intention | Married | p | No marriage  intention | Marriage  intention | Married | p | No marriage  intention | Marriage  intention | Married | p |
| Total | 9.0 | 87.4 | 3.5 |  | 9.0 | 43.2 | 47.9 |  | 12.5 | 20.9 | 66.6 |  | 10.3 | 42.3 | 47.4 |  |
| ***Education*** |  |  |  | < 0.0001 |  |  |  | < 0.0001 |  |  |  | < 0.0001 |  |  |  | < 0.0001 |
| High school or less | 10.5 | 82.1 | 7.5 |  | 11.8 | 39.8 | 48.4 |  | 16.2 | 24.3 | 59.4 |  | 13.4 | 40.1 | 46.5 |  |
| Vocational school/  short college | 9.6 | 88.7 | 1.7 |  | 10.0 | 42.0 | 48.1 |  | 11.2 | 22.4 | 66.4 |  | 10.4 | 41.4 | 48.3 |  |
| Undergraduate studies | 7.1 | 92.0 | 0.9 |  | 5.6 | 46.7 | 47.7 |  | 7.9 | 16.5 | 75.6 |  | 6.7 | 45.4 | 47.9 |  |
| Graduate studies | 8.3 | 91.7 | 0 |  | 5.2 | 49.4 | 45.4 |  | 9.0 | 11.9 | 79.1 |  | 6.7 | 41.7 | 51.6 |  |
| ***Occupational Status*** |  |  |  | < 0.0001 |  |  |  | < 0.0001 |  |  |  | < 0.0001 |  |  |  | < 0.0001 |
| Regular employee | 6.1 | 85.9 | 8.0 |  | 5.1 | 38.7 | 56.2 |  | 7.9 | 17.2 | 74.9 |  | 6.4 | 34.3 | 59.4 |  |
| Part-time/temporary worker | 14.2 | 81.9 | 3.8 |  | 19.6 | 64.0 | 16.4 |  | 28.9 | 40.8 | 30.3 |  | 21.4 | 60.2 | 18.4 |  |
| Business owner/ family business | 0 | 77.9 | 22.1 |  | 6.8 | 39.8 | 53.4 |  | 7.0 | 20.4 | 72.7 |  | 6.7 | 30.4 | 63.0 |  |
| Unemployed | 28 | 72.0 | 0 |  | 32.3 | 60.9 | 6.8 |  | 51.1 | 36.0 | 12.9 |  | 41.2 | 50.7 | 8.1 |  |
| Student | 7.3 | 92.7 | 0 |  | - | - | - |  | - | - | - |  | 7.4 | 92.0 | 0.5 |  |
| ***Annual Income  (in JPY 10,000s)*** |  |  |  | < 0.0001 |  |  |  | < 0.0001 |  |  |  | < 0.0001 |  |  |  | < 0.0001 |
| 0-99 | 10.5 | 89.2 | 0.3 |  | 23.0 | 55.7 | 21.3 |  | 33.4 | 32.9 | 33.8 |  | 19.2 | 67.0 | 13.8 |  |
| 100-199 | 11.2 | 86.5 | 2.3 |  | 17.9 | 59.1 | 22.9 |  | 21.2 | 41.2 | 37.6 |  | 17.2 | 60.9 | 21.9 |  |
| 200-299 | 4.1 | 84.2 | 11.6 |  | 10.0 | 55.1 | 34.9 |  | 18.1 | 35.2 | 46.7 |  | 11.1 | 55.2 | 33.7 |  |
| 300-399^a^ | 4.7 | 81.5 | 13.8 |  | 6.2 | 44.6 | 49.2 |  | 12.5 | 25.6 | 61.9 |  | 8.3 | 41.7 | 50.0 |  |
| 400-499 | - | - | - |  | 4.3 | 40.2 | 55.5 |  | 10.0 | 22.6 | 67.4 |  | 6.6 | 33.8 | 59.6 |  |
| 500-699 | - | - | - |  | 3.6 | 26.2 | 70.2 |  | 7.0 | 14.7 | 78.3 |  | 5.5 | 20.0 | 74.6 |  |
| ≥700 | - | - | - |  | 1.2 | 14.5 | 84.3 |  | 1.8 | 6.1 | 92.1 |  | 1.7 | 8.1 | 90.2 |  |
| ***Region of Residence*** |  |  |  | 0.3618 |  |  |  | 0.0014 |  |  |  | 0.1382 |  |  |  | 0.0041 |
| Hokkaido | 5.3 | 90.9 | 3.8 |  | 11.1 | 38.6 | 50.3 |  | 17.1 | 18.3 | 64.6 |  | 12.3 | 39.1 | 48.6 |  |
| Tohoku | 10.0 | 82.2 | 7.9 |  | 10.1 | 43.2 | 46.7 |  | 14.4 | 23.8 | 61.8 |  | 11.7 | 41.2 | 47.1 |  |
| Kanto | 9.4 | 88.8 | 1.8 |  | 10.0 | 46.6 | 43.3 |  | 12.5 | 20.8 | 66.7 |  | 10.8 | 44.9 | 44.3 |  |
| Chubu | 8.2 | 88.0 | 3.8 |  | 7.2 | 42.6 | 50.2 |  | 11.5 | 18.7 | 69.8 |  | 9.1 | 40.2 | 50.7 |  |
| Kinki | 7.9 | 88.4 | 3.7 |  | 10.5 | 42.9 | 46.6 |  | 12.3 | 20.5 | 67.3 |  | 10.8 | 41.5 | 47.7 |  |
| Chugoku/Shikoku | 10.3 | 82.1 | 7.7 |  | 7.9 | 40.1 | 52.0 |  | 14.9 | 18.5 | 66.6 |  | 10.7 | 40.3 | 49.0 |  |
| Kyushu/Okinawa | 10.4 | 86.8 | 2.7 |  | 6.1 | 37.8 | 56.1 |  | 10.4 | 28.0 | 61.6 |  | 8.4 | 42.2 | 49.4 |  |
|  |  |  |  |  |  |  |  |  |  |  |  |  |  |  |  |  |
| ***Area of Residence:  Population*** *Size* and Density |  |  |  | 0.2118 |  |  |  | 0.4173 |  |  |  | 0.0924 |  |  |  | 0.1506 |
| Non-densely inhabited area | 9.0 | 87.2 | 3.9 |  | 8.6 | 44.4 | 47.0 |  | 12.3 | 23.7 | 64.0 |  | 10.0 | 44.4 | 45.6 |  |
| <200,000 | 7.0 | 87.3 | 5.7 |  | 9.6 | 41.0 | 49.3 |  | 14.7 | 19.6 | 65.7 |  | 11.2 | 40.6 | 48.3 |  |
| 200,000 to <1,000,000 | 9.5 | 88.2 | 2.4 |  | 9.7 | 42.0 | 48.3 |  | 11.4 | 19.0 | 69.6 |  | 10.3 | 41.4 | 48.3 |  |
| ≥1,000,000 | 11.1 | 86.9 | 2.0 |  | 7.7 | 45.6 | 46.7 |  | 11.5 | 21.6 | 66.9 |  | 9.8 | 42.6 | 47.6 |  |

a. ≥300 for those aged 18-24 years.

**STable 6.** Proportion of women who are unmarried with marriage intention by sociodemographic characteristics, stratified by presence or absence of previous marriage. Numbers are shown in percent.

|  | **18-49 years** | | **18-24 years** | | **25-39 years** | | **40-49 years** | |
| --- | --- | --- | --- | --- | --- | --- | --- | --- |
|  | **Never-married** | **Previously-married** | **Never-married** | **Previously-married** | **Never-married** | **Previously-married** | **Never-married** | **Previously-married** |
| n (%) with marriage intention^a^ | 3308 (85%) | 581 (59%) | 1366 (94%) | 10 (80%) | 1441 (87%) | 215 (70%) | 501 (57%) | 356 (51%) |
| **Education** |  |  |  |  |  |  |  |  |
| High school or less | 27.9 | 57.3 | 28.1 | - | 25.7 | 53.7 | 36.5 | 60.1 |
| Vocational school/short college | 33.1 | 31.0 | 26.3 | - | 37.4 | 30.9 | 44.1 | 31.5 |
| Undergraduate studies | 37.8 | 10.7 | 45.1 | - | 35.0 | 15.4 | 18.4 | 6.7 |
| Graduate studies | 1.1 | 0.9 | 0.5 | - | 1.9 | 0 | 1 | 1.7 |
| **Occupational status** |  |  |  |  |  |  |  |  |
| Regular employee | 49.2 | 41.5 | 37.8 | - | 60.0 | 44.8 | 53.6 | 39.4 |
| Part-time/temporary worker | 22.0 | 43.7 | 15.4 | - | 27.7 | 44.1 | 26.6 | 43.5 |
| Business owner/family business | 1.9 | 4.3 | 0.4 | - | 3.0 | 2.8 | 4 | 5.9 |
| Unemployed | 7.3 | 10.5 | 4.4 | - | 8.3 | 8.3 | 15.7 | 11.2 |
| Student | 19.6 | 0 | 42.0 | - | 1.1 | 0 | - | - |
| **Annual income (in JPY 10,000s)** |  |  |  |  |  |  |  |  |
| 0 to <100 | 40.2 | 28.1 | 64.9 | - | 18.0 | 27.0 | 25.1 | 27.8 |
| 100 to <200 | 16.3 | 27.5 | 13.0 | - | 20.0 | 26.4 | 15.3 | 28.9 |
| 200 to <300 | 22.6 | 24.9 | 16.2 | - | 30.6 | 25.0 | 16.7 | 25.6 |
| 300 to <400 | 13.2 | 14.1 | 5.4 | - | 20.4 | 17.6 | 17.1 | 11.1 |
| 400 to <500^b^ | 4.5 | 2.4 | 0.5 | - | 7.7 | 2.7 | 9.8 | 2.2 |
| 500 to <600^c^ | 3.2 | 3.0 | - | - | 3.3 | 1.4 | 16.0 | 4.4 |
| 600 to <700 | - | - | - | - | - | - | - | - |
| ≥700 | - | - | - | - | - | - | - | - |
| **Desired life course of wife** |  |  |  |  |  |  |  |  |
| Working | 42.8 | 43.7 | 41.6 | - | 41.8 | 43.8 | 53.3 | 45.0 |
| Homemaker | 57.2 | 56.3 | 58.4 | - | 58.2 | 56.3 | 46.7 | 55.0 |
| **Region of residence** |  |  |  |  |  |  |  |  |
| Hokkaido | 3.2 | 3.8 | 3.0 | - | 3.3 | 4.6 | 3.1 | 3.3 |
| Tohoku | 6.8 | 14.1 | 6.2 | - | 7.9 | 15.9 | 4.5 | 12.2 |
| Kanto | 36.3 | 24.1 | 38.3 | - | 34.3 | 19.2 | 35.8 | 28.2 |
| Chubu | 17.9 | 18.8 | 17.5 | 0 | 19.0 | 19.9 | 14.9 | 18.8 |
| Kinki | 17.8 | 15.6 | 17.8 | - | 16.3 | 15.9 | 24.7 | 15.5 |
| Chugoku/Shikoku | 7.2 | 7.4 | 6.3 | - | 8.1 | 7.9 | 7.3 | 6.1 |
| Kyushu/Okinawa | 10.8 | 16.2 | 10.8 | - | 11.0 | 16.6 | 9.7 | 16.0 |
| **Area of residence: Population size and density** |  |  |  |  |  |  |  |  |
| Non-densely inhabited area | 25.4 | 35.0 | 26.2 | - | 26.0 | 42.4 | 19.4 | 29.3 |
| <200,000 | 24.2 | 20.3 | 26.0 | - | 22.9 | 17.9 | 22.2 | 22.1 |
| 200,000 to <1,000,000 | 29.4 | 29.1 | 28.9 | - | 29.5 | 23.8 | 31.3 | 33.1 |
| ≥1,000,000 | 20.9 | 15.6 | 18.9 | - | 21.6 | 15.9 | 27.1 | 15.5 |

Numbers for previously married women and men in the age group 18-24 years are not shown as the sample size was too small to allow for meaningful analyses.

a. n = number of individuals in sample with marriage intention. Percentage shows what percent of that category had marriage intention (e.g., 85% of never-married women aged 18-49 desired marriage).

b. ≥400 for those aged 18-24 years.

c. ≥500 for women in all age categories other than 18-24

**STable 7.** Proportion of men who are unmarried with marriage intention by sociodemographic characteristics, stratified by presence or absence of previous marriage. Numbers are shown in percent.

|  | **18-49 years** | | **18-24 years** | | **25-39 years** | | **40-49 years** | |
| --- | --- | --- | --- | --- | --- | --- | --- | --- |
|  | **Never-married** | **Previously-married** | **Never-married** | **Previously-married** | **Never-married** | **Previously-married** | **Never-married** | **Previously-married** |
| n (%) with marriage intention^a^ | 3829 (81%) | 282 (73%) | 1304 (91%) | 1 (100%) | 1776 (83%) | 101 (80%) | 749 (62%) | 180 (68%) |
| **Education** |  |  |  |  |  |  |  |  |
| High school or less | 38.1 | 61.6 | 36.9 | - | 34.7 | 67.5 | 51.7 | 58.2 |
| Vocational school/short college | 18.1 | 17.7 | 16.7 | - | 18.7 | 17.5 | 20.0 | 18.0 |
| Undergraduate studies | 38.7 | 18.7 | 43.6 | - | 38.8 | 12.5 | 25.9 | 22.1 |
| Graduate studies | 5.1 | 2.0 | 2.9 | - | 7.7 | 2.5 | 2.4 | 1.6 |
| **Occupational status** |  |  |  |  |  |  |  |  |
| Regular employee | 54.1 | 62.2 | 36.5 | - | 66.3 | 59.2 | 60.9 | 64.7 |
| Part-time/temporary worker | 14.0 | 13 | 9.0 | - | 17.2 | 17.1 | 16.4 | 10.3 |
| Business owner/family business | 5.5 | 15.5 | 1.3 | - | 7.4 | 11.8 | 10.4 | 18.1 |
| Unemployed | 6.8 | 8.3 | 3.7 | - | 7.7 | 10.5 | 12.0 | 6.9 |
| Student | 19.6 | 1.0 | 49.4 | - | 1.5 | 1.3 | 0.2 | 0 |
| **Annual income (in JPY 10,000s)** |  |  |  |  |  |  |  |  |
| 0 to <100 | 38.5 | 17.2 | 68.3 | - | 19.2 | 21.3 | 23.4 | 13.9 |
| 100 to <200 | 8.4 | 4.4 | 7.3 | - | 9.6 | 2.5 | 7.6 | 5.7 |
| 200 to <300 | 17.4 | 17.7 | 13.9 | - | 20.7 | 18.8 | 15.6 | 17.2 |
| 300 to <400 | 16.9 | 22.2 | 8.5 | - | 23.3 | 26.3 | 18.0 | 19.7 |
| 400 to <500^b^ | 10.8 | 14.8 | 2.0 | - | 16.4 | 13.8 | 16.0 | 15.6 |
| 500 to <600 | 4.9 | 10.3 | - | - | 7.2 | 7.5 | 9.6 | 12.3 |
| 600 to <700 | 1.7 | 7.9 | - | - | 2.0 | 7.5 | 4.9 | 8.2 |
| ≥700 | 1.5 | 5.4 | - | - | 1.6 | 2.5 | 4.9 | 7.4 |
| **Desired life course of wife** |  |  |  |  |  |  |  |  |
| Working | 46.4 | 43.7 | 41.2 | - | 48.5 | 36.0 | 53.3 | 49.5 |
| Homemaker | 53.6 | 56.3 | 58.8 | - | 51.5 | 64.0 | 46.7 | 50.5 |
| **Region of residence** |  |  |  |  |  |  |  |  |
| Hokkaido | 2.8 | 4.4 | 2.6 | - | 3.0 | 7.4 | 2.4 | 2.4 |
| Tohoku | 5.9 | 8.8 | 4.8 | - | 6.4 | 12.3 | 7.1 | 6.5 |
| Kanto | 37.7 | 28.3 | 38.6 | - | 37.4 | 29.6 | 36.6 | 26.8 |
| Chubu | 19.1 | 18.0 | 18.8 | - | 19.4 | 16.0 | 18.8 | 19.5 |
| Kinki | 15.6 | 15.1 | 16 | - | 15 | 16.0 | 16.5 | 14.6 |
| Chugoku/Shikoku | 9.0 | 11.2 | 9.5 | - | 9.5 | 11.1 | 6.5 | 11.4 |
| Kyushu/Okinawa | 9.8 | 14.1 | 9.7 | - | 9.1 | 7.4 | 12.1 | 18.7 |
|  |  |  |  |  |  |  |  |  |
| Non-densely inhabited area | 29.5 | 34.6 | 28.9 | - | 30.1 | 30.9 | 29.2 | 36.6 |
| <200,000 | 22.8 | 25.9 | 23.6 | - | 22.4 | 28.4 | 22.1 | 24.4 |
| 200,000 to <1,000,000 | 27.7 | 24.4 | 29.2 | - | 26.9 | 29.6 | 26.6 | 21.1 |
| ≥1,000,000 | 20.0 | 15.1 | 18.3 | - | 20.7 | 11.1 | 22.1 | 17.9 |

Numbers for previously married women and men in the age group 18-24 years are not shown as the sample size was too small to allow for meaningful analyses.

a. n = number of individuals in sample with marriage intention. Percentage shows what percent of that category had marriage intention (e.g., 85% of never-married women aged 18-49 desired marriage).

b. ≥400 for those aged 18-24 years.

**STable 8.** Proportion of women aged 18-24 years who are married, never-married with marriage intention, never-married without marriage intention, previously married with marriage intention and previously married without marriage intention by sociodemographic characteristics. Numbers are shown in percent.

|  | Married | Never-married with marriage intention | Never-married without marriage intention | Previously-married with marriage intention | Previously-married without marriage intention |
| --- | --- | --- | --- | --- | --- |
| Total | 5.9 | 87.7 | 5.8 | 0.5 | 0.1 |
| ***Education*** |  |  |  |  |  |
| High school or less | 13.9 | 78.8 | 5.9 | 1.0 | 0.4 |
| Vocational school/short college | 4.2 | 89.6 | 5.8 | 0.5 | - |
| Undergraduate studies | 1.0 | 93.3 | 5.5 | 0.1 | - |
| Graduate studies | - | 87.1 | 12.9 | - | - |
| ***Occupational Status*** |  |  |  |  |  |
| Regular employee | 3.4 | 93.6 | 2.6 | 0.4 | - |
| Part-time/temporary worker | 11.5 | 79.3 | 7.6 | 1.2 | 0.4 |
| Business owner/family business | 44.7 | 38.8 | 16.6 | - | - |
| Unemployed | 34.5 | 50.6 | 11.4 | 2.7 | 0.8 |
| Student | 0.2 | 93.6 | 6.2 | 0 | 0 |
| ***Annual Income (in JPY 10,000s)*** |  |  |  |  |  |
| 0-99 | 6.1 | 86.8 | 6.6 | 0.4 | 0.2 |
| 100-199 | 5.3 | 88.3 | 5.9 | 0.5 | - |
| 200-299 | 4.4 | 93.4 | 2.2 | 0 | - |
| 300-399 | 5.7 | 91.5 | 1.4 | 1.4 | - |
| 400-499 | 36.6 | 63.4 | - | - | - |
| ≥500 | 10.0 | 70.8 | 13.6 | 5.6 | - |
| ***Region of Residence*** |  |  |  |  |  |
| Hokkaido | 10.5 | 86.9 | 2.6 | 0 | - |
| Tohoku | 10.9 | 79.8 | 7.5 | 1.7 | - |
| Kanto | 3.9 | 90.4 | 5.4 | 0.3 | - |
| Chubu | 5.0 | 88.6 | 6.4 | - | - |
| Kinki | 5.3 | 88.6 | 5.5 | 0.3 | 0.4 |
| Chugoku/Shikoku | 12.2 | 75.5 | 9.7 | 1.8 | 0.8 |
| Kyushu/Okinawa | 6.5 | 89.1 | 3.9 | 0.5 | - |
| ***Area of Residence:*** |  |  |  |  |  |
| ***Population Size and Density*** |  |  |  |  |  |
| Non-densely inhabited area | 8.2 | 84.6 | 6.6 | 0.5 | 0.2 |
| <200,000 | 5.8 | 89.1 | 4.6 | 0.5 | - |
| 200,000 to <1,000,000 | 5.0 | 87.4 | 7.0 | 0.6 | - |
| ≥1,000,000 | 4.1 | 90.7 | 4.5 | 0.4 | 0.4 |

**STable 9.** Proportion of women aged 25-39 years who are married, never-married with marriage intention, never-married without marriage intention, previously married with marriage intention and previously married without marriage intention by sociodemographic characteristics. Numbers are shown in percent.

|  | Married | Never-married with marriage intention | Never-married without marriage intention | Previously-married with marriage intention | Previously-married without marriage intention |
| --- | --- | --- | --- | --- | --- |
| Total | 57.3 | 31.8 | 5.0 | 4.1 | 1.8 |
| ***Education*** |  |  |  |  |  |
| High school or less | 58.6 | 25.8 | 6.3 | 6.8 | 2.6 |
| Vocational school/short college | 59.8 | 31.0 | 4.1 | 3.3 | 1.7 |
| Undergraduate studies | 52.3 | 40.4 | 4.3 | 2.4 | 0.6 |
| Graduate studies | 60.0 | 26.8 | 8.2 | - | 5.0 |
| ***Occupational Status*** |  |  |  |  |  |
| Regular employee | 39.1 | 49.1 | 5.0 | 4.7 | 2.0 |
| Part-time/temporary worker | 58.1 | 27.9 | 5.8 | 5.6 | 2.6 |
| Business owner/family business | 67.4 | 21.8 | 5.9 | 2.8 | 2.1 |
| Unemployed | 84.4 | 10.7 | 3.3 | 1.4 | 0.2 |
| Student | 21.4 | 71.8 | 6.8 | - | - |
| ***Annual Income (in JPY 10,000s)*** |  |  |  |  |  |
| 0-99 | 79.7 | 13.1 | 3.9 | 2.6 | 0.8 |
| 100-199 | 44.8 | 37.3 | 7.9 | 6.4 | 3.7 |
| 200-299 | 32.0 | 54.7 | 5.7 | 5.8 | 1.8 |
| 300-399 | 34.3 | 52.7 | 5.3 | 6.1 | 1.6 |
| 400-499 | 47.7 | 44.3 | 3.1 | 1.8 | 3.1 |
| ≥500 | 59.9 | 30.9 | 4.6 | 2.2 | 2.4 |
| ***Region of Residence*** |  |  |  |  |  |
| Hokkaido | 54.8 | 30.0 | 6.7 | 5.5 | 3.0 |
| Tohoku | 49.3 | 33.4 | 6.9 | 8.6 | 1.8 |
| Kanto | 57.2 | 34.0 | 5.0 | 2.5 | 1.3 |
| Chubu | 60.2 | 29.7 | 3.9 | 4.0 | 2.3 |
| Kinki | 55.5 | 33.8 | 5.8 | 4.2 | 0.7 |
| Chugoku/Shikoku | 63.5 | 26.5 | 4.5 | 3.2 | 2.2 |
| Kyushu/Okinawa | 55.8 | 30.4 | 5.0 | 6.0 | 2.8 |
| ***Area of Residence:*** |  |  |  |  |  |
| ***Population Size and Density*** |  |  |  |  |  |
| Non-densely inhabited area | 57.4 | 29.4 | 5.1 | 6.1 | 2.1 |
| <200,000 | 58.8 | 30.5 | 5.4 | 3.2 | 2.1 |
| 200,000 to <1,000,000 | 56.3 | 33.2 | 5.1 | 3.5 | 1.9 |
| ≥1,000,000 | 56.9 | 34.7 | 4.5 | 3.3 | 0.7 |

**Stable 10.** Proportion of women aged 40-49 years who are married, never-married with marriage intention, never-married without marriage intention, previously married with marriage intention and previously married without marriage intention by sociodemographic characteristics. Numbers are shown in percent.

|  | Married | Never-married with marriage intention | Never-married without marriage intention | Previously-married with marriage intention | Previously-married without marriage intention |
| --- | --- | --- | --- | --- | --- |
| Total | 72.3 | 9.3 | 6.9 | 5.9 | 5.7 |
| ***Education*** |  |  |  |  |  |
| High school or less | 69.5 | 7.6 | 7.7 | 7.7 | 7.6 |
| Vocational school/short college | 74.2 | 10.6 | 5.9 | 4.7 | 4.7 |
| Undergraduate studies | 76.9 | 11.5 | 6.6 | 2.6 | 2.4 |
| Graduate studies | 64.4 | 9.4 | 12.8 | 10.0 | 3.4 |
| ***Occupational Status*** |  |  |  |  |  |
| Regular employee | 56.9 | 17.2 | 10.7 | 7.9 | 7.4 |
| Part-time/temporary worker | 76.8 | 5.7 | 5.7 | 5.8 | 6.0 |
| Business owner/family business | 77.5 | 5.9 | 4.3 | 5.4 | 6.8 |
| Unemployed | 82.8 | 6.4 | 5.1 | 2.8 | 2.9 |
| Student | 100 | - | - | - | - |
| ***Annual Income (in JPY 10,000s)*** |  |  |  |  |  |
| 0-99 | 85.0 | 4.8 | 4.0 | 3.5 | 2.8 |
| 100-199 | 69.9 | 6.8 | 5.4 | 8.4 | 9.4 |
| 200-299 | 50.1 | 12.5 | 14.9 | 12.4 | 10.1 |
| 300-399 | 51.0 | 20.4 | 11.5 | 8.8 | 8.3 |
| 400-499 | 62.1 | 18.9 | 11.5 | 3.1 | 4.4 |
| ≥500 | 64.6 | 19.1 | 8.4 | 3.0 | 4.9 |
| ***Region of Residence*** |  |  |  |  |  |
| Hokkaido | 68.1 | 8.9 | 13.1 | 5.9 | 4.0 |
| Tohoku | 68.0 | 5.9 | 5.8 | 10.5 | 9.7 |
| Kanto | 74.3 | 9.9 | 7.0 | 5.0 | 3.8 |
| Chubu | 75.9 | 6.9 | 4.8 | 5.5 | 6.8 |
| Kinki | 68.9 | 12.9 | 8.0 | 5.0 | 5.3 |
| Chugoku/Shikoku | 70.8 | 8.6 | 7.4 | 4.7 | 8.5 |
| Kyushu/Okinawa | 69.9 | 8.4 | 6.9 | 8.7 | 6.1 |
| ***Area of Residence:*** |  |  |  |  |  |
| ***Population Size and Density*** |  |  |  |  |  |
| Non-densely inhabited area | 73.4 | 6.7 | 6.5 | 6.3 | 7.0 |
| <200,000 | 73.8 | 8.9 | 6.3 | 5.7 | 5.4 |
| 200,000 to <1,000,000 | 71.9 | 9.8 | 6.0 | 6.6 | 5.7 |
| ≥1,000,000 | 69.7 | 12.3 | 9.3 | 4.4 | 4.3 |

**STable 11.** Proportion of men aged 18-24 years who are married, never-married with marriage intention, never-married without marriage intention, previously married with marriage intention and previously married without marriage intention by sociodemographic characteristics. Numbers are shown in percent.

|  | Married | Never-married with marriage intention | Never-married without marriage intention | Previously-married with marriage intention | Previously-married without marriage intention |
| --- | --- | --- | --- | --- | --- |
| Total | 3.6 | 87.3 | 9.0 | 0.1 | - |
| ***Education*** |  |  |  |  | - |
| High school or less | 7.6 | 82.0 | 10.4 | - | - |
| Vocational school/short college | 1.7 | 88.6 | 9.7 | - | - |
| Undergraduate studies | 0.9 | 91.8 | 7.1 | 0.2 | - |
| Graduate studies | - | 91.7 | 8.3 | - | - |
| ***Occupational Status*** |  |  |  |  |  |
| Regular employee | 8.1 | 85.8 | 6.1 | - | - |
| Part-time/temporary worker | 3.8 | 81.9 | 14.2 | - | - |
| Business owner/family business | 22.1 | 77.9 | - | - | - |
| Unemployed | - | 71.0 | 29.0 | - | - |
| Student | - | 92.5 | 7.3 | 0.2 | - |
| ***Annual Income (in JPY 10,000s)*** |  |  |  |  |  |
| 0-99 | 0.3 | 89.1 | 10.5 | 0.1 | - |
| 100-199 | 2.3 | 86.5 | 11.2 | - | - |
| 200-299 | 11.7 | 84.1 | 4.1 | - | - |
| 300-399 | 7.3 | 87.5 | 5.3 | - | - |
| 400-499 | 30.6 | 66.0 | 3.4 | - | - |
| ≥500 | 11.9 | 79.6 | 8.5 | - | - |
| ***Region of Residence*** |  |  |  |  |  |
| Hokkaido | 4.0 | 90.4 | 5.6 | - | - |
| Tohoku | 7.9 | 82.2 | 10.0 | - | - |
| Kanto | 1.9 | 88.5 | 9.4 | 0.2 | - |
| Chubu | 3.9 | 88.1 | 8.0 | - | - |
| Kinki | 3.7 | 88.3 | 8.0 | - | - |
| Chugoku/Shikoku | 7.7 | 82.1 | 10.3 | - | - |
| Kyushu/Okinawa | 2.7 | 86.7 | 10.5 | - | - |
| ***Area of Residence:*** |  |  |  |  |  |
| ***Population Size and Density*** |  |  |  |  |  |
| Non-densely inhabited area | 4.0 | 87.1 | 8.7 | 0.3 | - |
| <200,000 | 5.8 | 87.1 | 7.1 | - | - |
| 200,000 to <1,000,000 | 2.4 | 88.0 | 9.7 | - | - |
| ≥1,000,000 | 2.0 | 86.8 | 11.2 | - | - |

**STable 12.** Proportion of men aged 25-39 years who are married, never-married with marriage intention, never-married without marriage intention, previously married with marriage intention and previously married without marriage intention by sociodemographic characteristics. Numbers are shown in percent.

|  | Married | Never-married with marriage intention | Never-married without marriage intention | Previously-married with marriage intention | Previously-married without marriage intention |
| --- | --- | --- | --- | --- | --- |
| Total | 48.1 | 40.7 | 8.3 | 2.3 | 0.6 |
| ***Education*** |  |  |  |  |  |
| High school or less | 48.9 | 35.6 | 10.8 | 3.9 | 0.7 |
| Vocational school/short college | 48.3 | 39.7 | 8.8 | 2.1 | 1.0 |
| Undergraduate studies | 47.8 | 45.7 | 5.5 | 0.8 | 0.2 |
| Graduate studies | 45.6 | 48.2 | 4.8 | 1.0 | 0.4 |
| ***Occupational Status*** |  |  |  |  |  |
| Regular employee | 56.4 | 36.6 | 4.6 | 1.8 | 0.6 |
| Part-time/temporary worker | 16.6 | 61.0 | 18.5 | 3.4 | 0.5 |
| Business owner/family business | 53.4 | 36.6 | 6.1 | 3.2 | 0.7 |
| Unemployed | 6.3 | 54.0 | 35.2 | 4.0 | 0.5 |
| Student | 11.4 | 77.1 | 7.2 | 4.3 | - |
| ***Annual Income (in JPY 10,000s)*** |  |  |  |  |  |
| 0-99 | 21.5 | 52.4 | 22.3 | 3.2 | 0.6 |
| 100-199 | 23.2 | 57.8 | 18.2 | 0.9 | - |
| 200-299 | 35.3 | 52.3 | 8.9 | 2.7 | 0.9 |
| 300-399 | 49.3 | 41.8 | 5.5 | 2.8 | 0.6 |
| 400-499 | 55.7 | 38.0 | 3.4 | 1.9 | 1.0 |
| ≥500 | 73.1 | 22.0 | 2.9 | 1.8 | 0.3 |
| ***Region of Residence*** |  |  |  |  |  |
| Hokkaido | 51.4 | 33.4 | 10.5 | 4.6 | - |
| Tohoku | 46.7 | 38.9 | 8.8 | 4.2 | 1.3 |
| Kanto | 43.5 | 44.6 | 9.4 | 2.0 | 0.5 |
| Chubu | 50.6 | 40.5 | 6.4 | 1.9 | 0.6 |
| Kinki | 46.8 | 40.1 | 9.5 | 2.5 | 1.1 |
| Chugoku/Shikoku | 52.3 | 37.4 | 7.6 | 2.5 | 0.3 |
| Kyushu/Okinawa | 56.4 | 35.8 | 6.1 | 1.6 | - |
| ***Area of Residence:*** |  |  |  |  |  |
| ***Population Size and Density*** |  |  |  |  |  |
| Non-densely inhabited area | 47.2 | 41.8 | 8.0 | 2.5 | 0.5 |
| <200,000 | 49.9 | 37.9 | 8.6 | 2.7 | 0.8 |
| 200,000 to <1,000,000 | 48.5 | 39.5 | 9.0 | 2.5 | 0.6 |
| ≥1,000,000 | 46.8 | 44.1 | 7.4 | 1.3 | 0.3 |

**STable 13.** Proportion of men aged 40-49 years who are married, never-married with marriage intention, never-married without marriage intention, previously married with marriage intention and previously married without marriage intention by sociodemographic characteristics. Numbers are shown in percent.

|  | Married | Never-married with marriage intention | Never-married without marriage intention | Previously-married with marriage intention | Previously-married without marriage intention |
| --- | --- | --- | --- | --- | --- |
| Total | 66.9 | 16.4 | 10.3 | 4.4 | 2.1 |
| ***Education*** |  |  |  |  |  |
| High school or less | 59.9 | 18.6 | 12.8 | 5.5 | 3.2 |
| Vocational school/short college | 66.9 | 17.8 | 9.6 | 4.2 | 1.5 |
| Undergraduate studies | 75.6 | 13.4 | 7.2 | 3.1 | 0.7 |
| Graduate studies | 79.1 | 9.9 | 7.2 | 2.0 | 1.8 |
| ***Occupational Status*** |  |  |  |  |  |
| Regular employee | 75.1 | 13.3 | 6.2 | 3.8 | 1.6 |
| Part-time/temporary worker | 30.4 | 35.1 | 25.2 | 5.9 | 3.4 |
| Business owner/family business | 72.9 | 13.8 | 6.0 | 6.3 | 1.0 |
| Unemployed | 13.1 | 31.2 | 45.2 | 5.0 | 5.5 |
| Student | 23.1 | 40.2 | 36.7 | - | - |
| ***Annual Income (in JPY 10,000s)*** |  |  |  |  |  |
| 0-99 | 34.4 | 27.9 | 28.9 | 4.6 | 4.3 |
| 100-199 | 38.3 | 34.9 | 18.5 | 7.1 | 1.1 |
| 200-299 | 46.7 | 27.3 | 14.2 | 7.9 | 3.9 |
| 300-399 | 62.4 | 19.3 | 9.5 | 5.8 | 3.1 |
| 400-499 | 67.5 | 17.9 | 8.0 | 4.8 | 1.8 |
| ≥500 | 84.3 | 7.9 | 4.2 | 2.8 | 0.8 |
| ***Region of Residence*** |  |  |  |  |  |
| Hokkaido | 65.4 | 13.5 | 14.8 | 3.8 | 2.5 |
| Tohoku | 62.6 | 18.9 | 11.2 | 4.5 | 2.8 |
| Kanto | 66.7 | 17.4 | 10.8 | 3.4 | 1.7 |
| Chubu | 70.2 | 14.6 | 9.5 | 4.0 | 1.7 |
| Kinki | 67.8 | 16.3 | 10.4 | 3.7 | 1.8 |
| Chugoku/Shikoku | 67.2 | 12.8 | 10.2 | 5.9 | 3.9 |
| Kyushu/Okinawa | 61.8 | 19.5 | 8.3 | 8.3 | 2.1 |
| ***Area of Residence:*** |  |  |  |  |  |
| ***Population Size and Density*** |  |  |  |  |  |
| Non-densely inhabited area | 64.4 | 17.7 | 10.2 | 5.9 | 1.9 |
| <200,000 | 66.0 | 15.1 | 11.4 | 4.5 | 3.0 |
| 200,000 to <1,000,000 | 69.9 | 15.5 | 9.2 | 3.3 | 2.1 |
| ≥1,000,000 | 67.1 | 17.6 | 10.4 | 3.7 | 1.2 |

**STable 14.** Number of unmarried women and men aged 18-49 years, with marriage intention by income cut-off and number of potential partners available for hypergamy or homogamy.

| Women’s perspective | Women | | | Men | | | |  | |  | |
| --- | --- | --- | --- | --- | --- | --- | --- | --- | --- | --- | --- |
|  | Income level | (A)  N total (in 1000s) | Income level | | (B) N total (in 1000s) | (C) N with university education (in 1000s) | (A)/(B) N women per man available for homogamy/hypergamy | | (A)/(C) N women per man available for homogamy/hypergamy, including education | |  |
|  | 0 to <100 | 3319 | ≥0 | | 9828 | 4180 | 0.34 | | 0.79 | |  |
|  | 100 to <200 or lower | 4798 | ≥100 | | 6150 | 2382 | 0.78 | | 2.01 | |  |
|  | 200 to <300 or lower | 6734 | ≥200 | | 5347 | 2165 | 1.26 | | 3.11 | |  |
|  | 300 to <400 or lower | 7860 | ≥300 | | 3646 | 1678 | 2.16 | | 4.68 | |  |
|  | 400 to <500 or lower | 8221 | ≥400 | | 1963 | 1010 | 4.19 | | 8.14 | |  |
|  | 500 to <600 or lower | 8392 | ≥500 | | 885 | 508 | 9.49 | | 16.52 | |  |
|  | 600 to <700 or lower | 8460 | ≥600 | | 369 | 233 | 23.02 | | 36.33 | |  |
|  | ≥700 or lower | 8485 | ≥700 | | 167 | 130 | 50.78 | | 65.41 | |  |
| Men’s perspective | Men | | | Women | | | |  | |  | |
|  | Income level | (A) N total (in 1000s) | Income level | | (B) N total (in 1000s) | (C) N with university education (in 1000s) | (A)/(B) N men per woman available for homogamy/hypergamy | | (A)/(C) N men per woman available for homogamy/hypergamy, including education | |  |
|  | 100 to <200 or lower | 3678 | ≥0 | | 8485 | 3089 | 0.43 | | 1.19 | |  |
|  | 200 to <300 or lower | 4481 | ≥100 | | 5166 | 1688 | 0.87 | | 2.65 | |  |
|  | 300 to <400 or lower | 6182 | ≥200 | | 3687 | 1455 | 1.68 | | 4.25 | |  |
|  | 400 to <500 or lower | 7865 | ≥300 | | 1751 | 836 | 4.49 | | 9.41 | |  |
|  | 500 to <600 or lower | 8943 | ≥400 | | 624 | 321 | 14.32 | | 27.88 | |  |
|  | 600 to <700 or lower | 9460 | ≥500 | | 264 | 134 | 35.85 | | 70.57 | |  |
|  | ≥700 or lower | 9661 | ≥600 | | 92 | 52 | 104.46 | | 186.66 | |  |
|  | 100 to <200 or lower | 9828 | ≥700 | | 25 | 16 | 399.41 | | 609.62 | |  |

**STable 15.** Factors listed as “important” or “would consider” when choosing a partner among unmarried women with marriage intention in the National Fertility Survey 2015 by sociodemographic variables. Numbers are shown in percent.

|  | **Education** | | | **Occupation** | | | **Finances** | | | **Personality** | | | **Appearance** | | | **Mutual hobbies** | | | **Cooperative regarding one’s work** | | | **Skills/attitude towards chores/childrearing** | | | **Mean (SD) important** |
| --- | --- | --- | --- | --- | --- | --- | --- | --- | --- | --- | --- | --- | --- | --- | --- | --- | --- | --- | --- | --- | --- | --- | --- | --- | --- |
|  | Important | Would consider | p | Important | Would consider | p | Important | Would consider | p | Important | Would consider | p | Important | Would consider | p | Important | Would consider | p | Important | Would consider | p | Important | Would consider | p |  |
| Total | 9.5 | 44.4 |  | 28.1 | 56.8 |  | 38.3 | 55.7 |  | 88.7 | 10.5 |  | 14.6 | 63.3 |  | 23.2 | 51.6 |  | 47.0 | 45.7 |  | 54.4 | 42.1 |  | 3.0 (1.9) |
| ***Age group*** |  |  |  |  |  |  |  |  |  |  |  |  |  |  |  |  |  |  |  |  |  |  |  |  |  |
| 18-24 | 11.7 | 44.4 | 0.003 | 32.0 | 54.4 | <0.001 | 42.7 | 51.4 | <0.001 | 89.1 | 9.9 | 0.124 | 17.7 | 61.8 | 0.002 | 26.1 | 51.1 | 0.002 | 54.8 | 41.1 | <0.001 | 62.9 | 35.0 | <0.001 | 3.4 (2.0) |
| 25-29 | 10.5 | 44.4 |  | 30.3 | 57.4 |  | 39.0 | 56.3 |  | 90.6 | 9.2 |  | 13.0 | 64.5 |  | 21.9 | 51.5 |  | 45.5 | 47.6 |  | 55.3 | 42.6 |  | 3.1 (1.9) |
| 30-34 | 7.7 | 41.2 |  | 23.9 | 60.2 |  | 34.8 | 60.0 |  | 86.8 | 12.7 |  | 15.7 | 63.8 |  | 20.8 | 53.8 |  | 43.3 | 48.3 |  | 52.4 | 43.6 |  | 2.8 (1.8) |
| 34-39 | 5.6 | 47.4 |  | 25.7 | 56.8 |  | 31.3 | 61.5 |  | 86.2 | 12.5 |  | 9.6 | 66.6 |  | 16.1 | 52.3 |  | 36.5 | 52.3 |  | 39.5 | 53.6 |  | 2.5 (1.7) |
| 40-44 | 5.3 | 44.5 |  | 21.3 | 55.9 |  | 34.5 | 57.6 |  | 86.4 | 11.7 |  | 9.1 | 66.2 |  | 23.9 | 46.2 |  | 35.0 | 49.8 |  | 40.0 | 54.2 |  | 2.5 (1.7) |
| 45-49 | 6.7 | 45.9 |  | 16.4 | 64.6 |  | 29.9 | 61.9 |  | 89.7 | 9.2 |  | 13.3 | 59.5 |  | 23.6 | 55.9 |  | 41.5 | 48.2 |  | 42.8 | 49.5 |  | 2.6 (1.8) |
| ***Education*** |  |  |  |  |  |  |  |  |  |  |  |  |  |  |  |  |  |  |  |  |  |  |  |  |  |
| High school or less | 4.8 | 31.8 | <0.001 | 21.9 | 55.8 | <0.001 | 34.7 | 57.3 | 0.001 | 83.7 | 14.6 | <0.001 | 13.9 | 61.1 | 0.129 | 22.4 | 49.8 | 0.401 | 39.1 | 50.6 | <0.001 | 50.6 | 45.1 | 0.020 | 2.7 (1.8) |
| Vocational school/ short college | 5.6 | 45.8 |  | 26.5 | 60.0 |  | 37.2 | 58.0 |  | 88.8 | 10.4 |  | 14.5 | 64.0 |  | 24.2 | 52.7 |  | 48.3 | 45.0 |  | 54.7 | 41.8 |  | 3.0 (1.9) |
| Undergraduate studies | 17.0 | 53.2 |  | 35.1 | 54.5 |  | 42.3 | 52.4 |  | 93.2 | 6.6 |  | 15.5 | 64.6 |  | 23.0 | 52.1 |  | 52.1 | 42.7 |  | 56.8 | 40.3 |  | 3.3 (2.0) |
| Graduate studies | 23.5 | 70.6 |  | 38.2 | 55.9 |  | 44.1 | 50.0 |  | 88.2 | 11.8 |  | 11.8 | 73.5 |  | 23.5 | 52.9 |  | 67.6 | 23.5 |  | 73.5 | 26.5 |  | 3.7 (2.0) |
| ***Occupational Status*** |  |  |  |  |  |  |  |  |  |  |  |  |  |  |  |  |  |  |  |  |  |  |  |  |  |
| Regular employee | 9.9 | 46.7 | <0.001 | 29.8 | 57.2 | <0.001 | 38.8 | 56.4 | <0.001 | 89.1 | 10.2 | <0.001 | 13.9 | 64.8 | 0.004 | 20.7 | 52.4 | 0.120 | 45.2 | 48.3 | <0.001 | 55.0 | 42.3 | <0.001 | 3.0 (1.9) |
| Part-time/temporary worker | 7.5 | 35.9 |  | 24.2 | 56.9 |  | 36.7 | 55.4 |  | 88.2 | 10.9 |  | 13.3 | 63.9 |  | 25.3 | 51.5 |  | 40.4 | 49.4 |  | 50.5 | 44.7 |  | 2.9 (1.8) |
| Business owner/family business | 3.1 | 41.5 |  | 13.8 | 67.7 |  | 30.8 | 61.5 |  | 92.3 | 6.2 |  | 27.7 | 50.8 |  | 23.1 | 55.4 |  | 66.2 | 26.2 |  | 46.2 | 46.2 |  | 3.0 (1.7) |
| Unemployed | 2.6 | 39.2 |  | 16.0 | 60.6 |  | 28.6 | 64.1 |  | 80.3 | 18.0 |  | 10.0 | 62.6 |  | 20.7 | 52.6 |  | 31.7 | 54.3 |  | 40.1 | 53.7 |  | 2.3 (1.8) |
| Student | 14.7 | 53.1 |  | 36.3 | 53.5 |  | 44.9 | 50.2 |  | 92.8 | 6.6 |  | 17.7 | 62.1 |  | 26.2 | 50.7 |  | 65.3 | 33.0 |  | 65.7 | 33.0 |  | 3.6 (2.0) |
| ***Annual Income (in JPY 10,000s)*** |  |  |  |  |  |  |  |  |  |  |  |  |  |  |  |  |  |  |  |  |  |  |  |  |  |
| 0-99 | 10.1 | 44.8 | <0.001 | 28.4 | 55.4 | 0.263 | 38.6 | 54.5 | 0.522 | 87.2 | 11.9 | 0.406 | 15.5 | 61.2 | 0.444 | 25.2 | 50.8 | 0.263 | 52.2 | 40.4 | <0.001 | 56.6 | 39.3 | 0.348 | 3.1 (2.0) |
| 100-199 | 6.7 | 36.0 |  | 25.3 | 58.6 |  | 36.2 | 57.3 |  | 88.9 | 9.8 |  | 14.7 | 63.6 |  | 24.9 | 48.6 |  | 40.6 | 50.8 |  | 53.7 | 43.9 |  | 2.9 (1.8) |
| 200-299 | 9.0 | 42.3 |  | 30.3 | 55.1 |  | 39.5 | 56.1 |  | 89.6 | 9.9 |  | 14.0 | 63.1 |  | 20.9 | 53.6 |  | 42.6 | 49.7 |  | 52.7 | 44.0 |  | 3.0 (1.8) |
| 300-399 | 11.4 | 53.2 |  | 29.4 | 59.0 |  | 40.3 | 55.1 |  | 90.5 | 8.8 |  | 12.4 | 68.8 |  | 22.3 | 52.7 |  | 47.8 | 45.9 |  | 54.9 | 41.7 |  | 3.1 (1.8) |
| 400-499^a^ | 12.2 | 52.7 |  | 29.8 | 58.0 |  | 36.6 | 57.3 |  | 88.5 | 11.5 |  | 16.9 | 64.6 |  | 15.3 | 55.7 |  | 45.8 | 50.4 |  | 52.7 | 44.3 |  | 3.0 (2.0) |
| ≥500 | 10.6 | 62.8 |  | 28.4 | 62.1 |  | 33.7 | 58.9 |  | 93.7 | 6.3 |  | 11.6 | 66.3 |  | 24.2 | 48.4 |  | 52.6 | 43.2 |  | 45.3 | 50.5 |  | 3.0 (1.8) |
| ***Previously married*** |  |  |  |  |  |  |  |  |  |  |  |  |  |  |  |  |  |  |  |  |  |  |  |  |  |
| No | 10.1 | 45.4 | <0.001 | 29.2 | 56.5 | <0.001 | 38.6 | 55.4 | 0.770 | 89.2 | 10.1 | 0.182 | 15.3 | 63.1 | 0.004 | 23.4 | 51.4 | 0.874 | 47.6 | 45.6 | 0.008 | 54.8 | 42.0 | 0.053 | 3.1 (1.9) |
| Yes | 3.6 | 36.5 |  | 20.1 | 59.3 |  | 37.1 | 57.4 |  | 86.7 | 11.8 |  | 9.1 | 64.2 |  | 22.4 | 51.2 |  | 42.6 | 46.2 |  | 51.8 | 42.4 |  | 2.7 (1.8) |
| **Preferred life course of wife** |  |  |  |  |  |  |  |  |  |  |  |  |  |  |  |  |  |  |  |  |  |  |  |  |  |
| Working | 9.9 | 47.2 | 0.015 | 27.9 | 57.9 | 0.652 | 36.4 | 57.3 | 0.043 | 88.3 | 11.1 | 0.316 | 14.4 | 64.0 | 0.978 | 23.6 | 52.9 | 0.142 | 56.7 | 39.9 | <0.001 | 57.2 | 39.6 | 0.112 | 3.1 (1.9) |
| Homemaker | 9.1 | 42.6 |  | 29.0 | 56.2 |  | 40.2 | 55.0 |  | 90.0 | 9.4 |  | 14.6 | 64.1 |  | 22.7 | 50.6 |  | 40.4 | 49.6 |  | 53.4 | 43.4 |  | 3.0 (1.9) |
| ***Region of Residence*** |  |  |  |  |  |  |  |  |  |  |  |  |  |  |  |  |  |  |  |  |  |  |  |  |  |
| Hokkaido | 6.8 | 35.9 | <0.001 | 26.2 | 55.3 | 0.276 | 33.0 | 61.2 | 0.086 | 84.5 | 11.7 | 0.046 | 18.4 | 52.4 | 0.281 | 25.2 | 46.6 | 0.183 | 43.7 | 48.5 | 0.664 | 46.6 | 46.6 | 0.161 | 2.8 (1.9) |
| Tohoku | 5.9 | 37.2 |  | 23.4 | 54.8 |  | 34.3 | 56.9 |  | 86.2 | 13.0 |  | 11.7 | 60.3 |  | 20.1 | 50.6 |  | 46.0 | 44.8 |  | 53.2 | 41.4 |  | 2.8 (1.8) |
| Kanto | 11.9 | 47.7 |  | 30.0 | 56.3 |  | 39.6 | 53.4 |  | 89.5 | 9.9 |  | 15.3 | 64.6 |  | 26.2 | 50.0 |  | 49.3 | 44.4 |  | 55.5 | 41.0 |  | 3.2 (1.9) |
| Chubu | 7.6 | 44.2 |  | 27.2 | 58.1 |  | 35.2 | 60.0 |  | 86.8 | 12.3 |  | 13.7 | 63.2 |  | 23.3 | 52.0 |  | 46.8 | 45.4 |  | 53.4 | 43.1 |  | 2.9 (1.9) |
| Kinki | 9.5 | 46.3 |  | 27.7 | 57.3 |  | 38.6 | 57.0 |  | 90.6 | 8.5 |  | 15.0 | 63.7 |  | 20.7 | 55.3 |  | 45.0 | 48.1 |  | 51.9 | 45.5 |  | 3.0 (1.9) |
| Chugoku/Shikoku | 9.2 | 41.5 |  | 28.4 | 55.0 |  | 41.5 | 51.5 |  | 91.3 | 7.9 |  | 14.4 | 63.8 |  | 19.2 | 52.8 |  | 42.4 | 48.0 |  | 54.1 | 42.4 |  | 3.0 (1.9) |
| Kyushu/Okinawa | 8.4 | 40.7 |  | 28.0 | 58.2 |  | 40.7 | 54.5 |  | 87.3 | 12.1 |  | 14.4 | 64.1 |  | 21.7 | 51.0 |  | 48.0 | 44.9 |  | 59.9 | 37.5 |  | 3.1 (1.9) |
| ***Area of Residence: Population*** *Size* and Density |  |  |  |  |  |  |  |  |  |  |  |  |  |  |  |  |  |  |  |  |  |  |  |  |  |
| Non-densely inhabited area | 6.7 | 39.5 | <0.001 | 25.0 | 56.7 | 0.025 | 37.4 | 55.7 | 0.238 | 84.8 | 13.9 | 0.001 | 14.5 | 63.6 | 0.771 | 20.9 | 52.3 | 0.410 | 47.2 | 45.8 | 0.965 | 53.9 | 43.2 | 0.209 | 2.9 (1.9) |
| <200,000 | 9.8 | 44.8 |  | 29.9 | 57.0 |  | 38.8 | 56.6 |  | 89.1 | 9.7 |  | 15.3 | 62.4 |  | 23.5 | 49.9 |  | 46.0 | 46.8 |  | 58.1 | 38.2 |  | 3.1 (1.9) |
| 200,000 to <1,000,000 | 11.6 | 46.2 |  | 30.2 | 55.9 |  | 40.5 | 53.3 |  | 90.8 | 8.6 |  | 15.6 | 62.3 |  | 24.1 | 51.5 |  | 48.0 | 44.3 |  | 53.9 | 42.7 |  | 3.1 (1.9) |
| ≥1,000,000 | 9.6 | 47.6 |  | 27.2 | 57.9 |  | 35.6 | 58.2 |  | 90.1 | 9.7 |  | 12.7 | 65.5 |  | 24.5 | 52.5 |  | 46.5 | 46.2 |  | 51.5 | 44.3 |  | 3.0 (1.9) |

Participants with missing data were dropped from the analysis by item. N (%) for missing values were 56 (1.8) for education, 58 (1.8) for occupation, 56 (1.8) for finances, 55 (1.7) for personality, 61 (1.9) for appearance, 56 (1.8) for mutual hobbies, 58 (1.8) for cooperation regarding one’s work, and 64 (2) for skills/attitude towards chores/childrearing. Mean (SD) items considered to be “important” were calculated excluding participants who had not provided an answer for any item (n=51[1.6%])

**STable 16.** Factors listed as “important” or “would consider” when choosing a partner among unmarried men with marriage intention in the National Fertility Survey 2015 by sociodemographic variables. Numbers are shown in percent.

|  | **Education** | | | **Occupation** | | | **Finances** | | | **Personality** | | | **Appearance** | | | **Mutual hobbies** | | | **Cooperative regarding one’s work** | | | **Skills/attitude towards chores/childrearing** | | | **Mean (SD) important** |
| --- | --- | --- | --- | --- | --- | --- | --- | --- | --- | --- | --- | --- | --- | --- | --- | --- | --- | --- | --- | --- | --- | --- | --- | --- | --- |
|  | Important | Would consider | p | Important | Would consider | p | Important | Would consider | p | Important | Would consider | p | Important | Would consider | p | Important | Would consider | p | Important | Would consider | p | Important | Would consider | p |  |
| Total | 3.0 | 25.6 |  | 5.4 | 38.5 |  | 4.3 | 36.2 |  | 75.1 | 21.3 |  | 22.6 | 61.5 |  | 19.1 | 53.1 |  | 39.3 | 50.1 |  | 43.9 | 50.0 |  | 2.1 (1.6) |
| ***Age group*** |  |  |  |  |  |  |  |  |  |  |  |  |  |  |  |  |  |  |  |  |  |  |  |  |  |
| 18-24 | 3.7 | 30.0 | <0.001 | 6.2 | 45.0 | <0.001 | 4.9 | 39.0 | <0.001 | 79.7 | 17.4 | <0.001 | 26.9 | 60.2 | <0.001 | 22.1 | 55.1 | <0.001 | 46.6 | 45.3 | <0.001 | 49.2 | 45.7 | <0.001 | 2.4 (1.7) |
| 25-29 | 5.3 | 24.8 |  | 8.2 | 41.5 |  | 5.9 | 40.2 |  | 75.6 | 21.5 |  | 24.4 | 62.0 |  | 22.9 | 51.5 |  | 40.7 | 48.9 |  | 49.3 | 46.6 |  | 2.3 (1.7) |
| 30-34 | 1.6 | 23.7 |  | 3.8 | 35.4 |  | 3.4 | 32.4 |  | 76.4 | 19.4 |  | 18.3 | 63.8 |  | 18.1 | 51.0 |  | 36.8 | 50.7 |  | 40.2 | 53.5 |  | 2.0 (1.5) |
| 34-39 | 0.8 | 21.6 |  | 2.3 | 32.3 |  | 2.3 | 35.0 |  | 69.2 | 27.5 |  | 19.0 | 61.5 |  | 15.6 | 51.3 |  | 34.1 | 53.9 |  | 37.3 | 54.6 |  | 1.8 (1.4) |
| 40-44 | 1.2 | 21.1 |  | 4.3 | 29.7 |  | 3.7 | 27.6 |  | 68.5 | 27.2 |  | 15.8 | 63.2 |  | 10.8 | 57.4 |  | 27.0 | 61.2 |  | 31.5 | 60.1 |  | 1.6 (1.5) |
| 45-49 | 2.8 | 23.5 |  | 3.2 | 28.3 |  | 2.8 | 33.1 |  | 67.3 | 25.9 |  | 20.7 | 59.4 |  | 13.6 | 50.0 |  | 30.4 | 54.4 |  | 38.8 | 52.8 |  | 1.8 (1.5) |
| ***Education*** |  |  |  |  |  |  |  |  |  |  |  |  |  |  |  |  |  |  |  |  |  |  |  |  |  |
| High school or less | 1.9 | 17.1 | <0.001 | 4.4 | 30.1 | <0.001 | 4.4 | 32.4 | 0.010 | 69.2 | 26.3 | <0.001 | 20.5 | 59.8 | <0.001 | 16.8 | 53.3 | 0.066 | 34.1 | 53.8 | <0.001 | 43.7 | 50.2 | 0.057 | 1.9 (1.6) |
| Vocational school/ short college | 2.1 | 24.1 |  | 5.1 | 37.0 |  | 4.8 | 36.4 |  | 73.4 | 22.1 |  | 19.4 | 64.7 |  | 22.4 | 51.1 |  | 37.9 | 50.3 |  | 40.1 | 52.1 |  | 2.0 (1.6) |
| Undergraduate studies | 3.9 | 33.5 |  | 6.2 | 46.0 |  | 4.1 | 39.4 |  | 81.1 | 16.6 |  | 26.0 | 61.1 |  | 19.3 | 54.3 |  | 43.7 | 47.4 |  | 44.8 | 49.6 |  | 2.3 (1.6) |
| Graduate studies | 10.0 | 43.1 |  | 8.1 | 55.0 |  | 3.8 | 42.5 |  | 83.1 | 15.6 |  | 25.0 | 67.5 |  | 21.3 | 49.4 |  | 51.3 | 42.5 |  | 50.6 | 47.5 |  | 2.5 (1.6) |
| ***Occupational Status*** |  |  |  |  |  |  |  |  |  |  |  |  |  |  |  |  |  |  |  |  |  |  |  |  |  |
| Regular employee | 3.1 | 24.8 | <0.001 | 5.2 | 39.4 | <0.001 | 3.8 | 37.1 | 0.010 | 73.3 | 22.7 | <0.001 | 22.0 | 61.9 | 0.016 | 17.4 | 54.7 | 0.001 | 36.9 | 53.3 | <0.001 | 42.8 | 51.5 | 0.014 | 2.0 (1.6) |
| Part-time/temporary worker | 2.6 | 22.6 |  | 4.9 | 32.1 |  | 4.4 | 36.5 |  | 75.3 | 20.9 |  | 20.7 | 63.5 |  | 20.5 | 52.4 |  | 33.0 | 51.2 |  | 43.0 | 51.2 |  | 2.0 (1.7) |
| Business owner/family business | 1.6 | 15.9 |  | 3.2 | 29.1 |  | 2.6 | 24.3 |  | 71.7 | 25.1 |  | 22.2 | 63.5 |  | 18.9 | 46.3 |  | 51.3 | 38.7 |  | 47.1 | 43.3 |  | 2.2 (1.6) |
| Unemployed | 1.8 | 21.8 |  | 4.5 | 34.1 |  | 6.8 | 36.8 |  | 74.4 | 22.4 |  | 19.5 | 58.6 |  | 17.7 | 50.0 |  | 33.2 | 49.3 |  | 39.7 | 51.6 |  | 2.0 (1.6) |
| Student | 4.1 | 36.8 |  | 6.7 | 48.0 |  | 4.8 | 37.5 |  | 84.9 | 13.4 |  | 26.6 | 61.8 |  | 23.3 | 55.2 |  | 49.5 | 45.1 |  | 49.2 | 46.3 |  | 2.5 (1.7) |
| ***Annual Income (in JPY 10,000s)*** |  |  |  |  |  |  |  |  |  |  |  |  |  |  |  |  |  |  |  |  |  |  |  |  |  |
| 0-99 | 3.4 | 30.2 | <0.001 | 6.4 | 42.5 | 0.001 | 5.5 | 38.2 | 0.009 | 78.5 | 18.6 | 0.041 | 24.9 | 59.5 | 0.001 | 21.4 | 52.5 | 0.014 | 43.7 | 46.0 | 0.003 | 46.1 | 47.9 | 0.060 | 2.3 (1.7) |
| 100-199 | 2.3 | 18.7 |  | 6.5 | 29.4 |  | 6.1 | 33.6 |  | 71.4 | 24.4 |  | 19.8 | 61.8 |  | 18.3 | 50.4 |  | 33.0 | 51.7 |  | 44.6 | 45.8 |  | 2.0 (1.7) |
| 200-299 | 2.7 | 19.1 |  | 4.5 | 33.2 |  | 2.7 | 35.9 |  | 72.1 | 22.3 |  | 22.6 | 57.2 |  | 17.1 | 54.7 |  | 38.3 | 50.4 |  | 45.2 | 48.4 |  | 2.0 (1.6) |
| 300-399 | 2.7 | 23.5 |  | 4.0 | 39.6 |  | 4.0 | 38.2 |  | 72.8 | 23.8 |  | 21.3 | 62.4 |  | 20.4 | 54.2 |  | 37.4 | 52.9 |  | 40.3 | 54.3 |  | 2.0 (1.6) |
| 400-499^a^ | 3.7 | 27.6 |  | 6.2 | 38.6 |  | 3.4 | 32.6 |  | 77.2 | 19.7 |  | 21.4 | 68.2 |  | 16.9 | 51.8 |  | 35.6 | 53.4 |  | 41.4 | 53.5 |  | 2.1 (1.6) |
| 500-699 | 1.7 | 28.0 |  | 3.8 | 39.0 |  | 2.1 | 33.5 |  | 72.9 | 24.6 |  | 17.4 | 68.6 |  | 12.3 | 58.3 |  | 33.9 | 58.1 |  | 39.1 | 56.6 |  | 1.8 (1.5) |
| ≥700 | 7.1 | 32.1 |  | 5.4 | 41.1 |  | 3.6 | 23.2 |  | 78.6 | 17.9 |  | 30.4 | 64.3 |  | 21.4 | 37.5 |  | 42.9 | 51.8 |  | 39.3 | 53.6 |  | 2.3 (1.3) |
| ***Previously married*** |  |  |  |  |  |  |  |  |  |  |  |  |  |  |  |  |  |  |  |  |  |  |  |  |  |
| No | 3.1 | 25.8 | 0.424 | 5.3 | 39.4 | 0.002 | 4.3 | 36.6 | 0.073 | 75.5 | 21.2 | 0.231 | 22.6 | 61.4 | 0.323 | 19.1 | 53.5 | 0.380 | 39.3 | 50.0 | 0.677 | 43.9 | 50.1 | 0.855 | 2.1 (1.6) |
| Yes | 1.5 | 25.4 |  | 4.6 | 27.4 |  | 3.1 | 29.6 |  | 72.7 | 21.7 |  | 22.3 | 65.5 |  | 17.8 | 50.3 |  | 38.1 | 52.8 |  | 44.2 | 50.8 |  | 2.0 (1.6) |
| **Preferred life course of wife** |  |  |  |  |  |  |  |  |  |  |  |  |  |  |  |  |  |  |  |  |  |  |  |  |  |
| Working | 3.8 | 26.4 | 0.033 | 6.6 | 38.5 | 0.011 | 5.7 | 39.7 | <0.001 | 71.0 | 24.5 | <0.001 | 20.7 | 61.3 | 0.001 | 18.4 | 53.9 | 0.709 | 37.0 | 50.8 | <0.001 | 37.7 | 54.7 | <0.001 | 2.0 (1.7) |
| Homemaker | 2.2 | 26.2 |  | 4.3 | 41.0 |  | 2.9 | 34.9 |  | 79.7 | 18.4 |  | 24.4 | 61.9 |  | 19.5 | 53.5 |  | 42.4 | 49.7 |  | 50.3 | 45.9 |  | 2.3 (1.5) |
| ***Region of Residence*** |  |  |  |  |  |  |  |  |  |  |  |  |  |  |  |  |  |  |  |  |  |  |  |  |  |
| Hokkaido | 2.0 | 26.3 | 0.013 | 5.1 | 33.3 | 0.143 | 5.1 | 33.3 | 0.560 | 83.8 | 12.1 | 0.004 | 30.3 | 51.5 | <0.001 | 17.2 | 54.5 | 0.858 | 41.4 | 45.5 | 0.501 | 52.5 | 41.4 | 0.066 | 2.4 (1.6) |
| Tohoku | 1.0 | 19.0 |  | 5.0 | 37.5 |  | 5.5 | 39.5 |  | 68.5 | 28.0 |  | 21.5 | 57.5 |  | 16.5 | 52.5 |  | 32.0 | 55.0 |  | 35.7 | 58.8 |  | 1.9 (1.6) |
| Kanto | 4.3 | 28.1 |  | 6.3 | 40.6 |  | 5.0 | 37.3 |  | 77.6 | 19.6 |  | 24.2 | 61.6 |  | 20.1 | 53.4 |  | 40.3 | 49.9 |  | 43.3 | 51.2 |  | 2.2 (1.7) |
| Chubu | 2.4 | 23.8 |  | 4.5 | 39.4 |  | 3.5 | 34.8 |  | 73.9 | 21.8 |  | 18.8 | 62.0 |  | 19.0 | 52.8 |  | 38.7 | 51.2 |  | 44.2 | 48.7 |  | 2.0 (1.6) |
| Kinki | 2.3 | 25.3 |  | 4.3 | 34.4 |  | 3.3 | 33.3 |  | 75.8 | 21.3 |  | 24.3 | 62.5 |  | 18.9 | 52.3 |  | 39.5 | 48.4 |  | 45.2 | 49.1 |  | 2.1 (1.6) |
| Chugoku/Shikoku | 2.0 | 25.9 |  | 6.3 | 41.2 |  | 4.0 | 36.9 |  | 75.4 | 21.9 |  | 22.6 | 67.1 |  | 17.3 | 57.5 |  | 43.0 | 47.0 |  | 48.0 | 48.0 |  | 2.2 (1.6) |
| Kyushu/Okinawa | 3.0 | 24.1 |  | 4.9 | 35.4 |  | 4.0 | 37.5 |  | 68.3 | 25.3 |  | 19.3 | 59.0 |  | 20.1 | 50.0 |  | 36.9 | 52.4 |  | 42.2 | 48.9 |  | 2.0 (1.7) |
| ***Area of Residence: Population*** *Size* and Density |  |  |  |  |  |  |  |  |  |  |  |  |  |  |  |  |  |  |  |  |  |  |  |  |  |
| Non-densely inhabited area | 2.4 | 22.8 | 0.057 | 4.8 | 38.3 | 0.034 | 4.8 | 37.4 | 0.027 | 68.6 | 26.7 | <0.001 | 19.5 | 61.5 | 0.001 | 18.6 | 53.5 | 0.590 | 37.0 | 51.3 | 0.241 | 42.4 | 50.5 | 0.036 | 2.0 (1.7) |
| <200,000 | 2.9 | 24.6 |  | 5.8 | 34.2 |  | 3.7 | 34.5 |  | 73.9 | 22.1 |  | 20.3 | 64.3 |  | 17.5 | 53.5 |  | 37.5 | 51.9 |  | 43.9 | 49.7 |  | 2.0 (1.6) |
| 200,000 to <1,000,000 | 3.4 | 28.1 |  | 5.5 | 42.6 |  | 4.8 | 39.3 |  | 79.5 | 17.6 |  | 24.7 | 61.1 |  | 19.8 | 54.0 |  | 41.9 | 48.6 |  | 48.0 | 47.3 |  | 2.3 (1.6) |
| ≥1,000,000 | 3.6 | 27.6 |  | 5.8 | 38.4 |  | 3.6 | 32.1 |  | 80.4 | 17.4 |  | 27.1 | 58.7 |  | 21.0 | 51.0 |  | 41.4 | 48.3 |  | 40.6 | 53.5 |  | 2.2 (1.7) |

Participants with missing data were dropped from the analysis by item. N (%) for missing values were 80 (2.4) for education, 81 (2.4) for occupation, 82 (2.4) for finances, 77 (2.3) for personality, 83 (2.5) for appearance, 81 (2.4) for mutual hobbies, 86 (2.6) for cooperation regarding one’s work, and 89 (2.6) for skills/attitude towards chores/childrearing. Mean (SD) items considered to be “important” were calculated excluding participants who had not provided an answer for any item (n=74[2.2%])

**STable 17.** Factors listed as “important” or “would consider” when choosing a partner among unmarried women with marriage intention, aged 18-24 years, in the National Fertility Survey 2015 by sociodemographic variables. Numbers are shown in percent.

|  | **Education** | | | **Occupation** | | | **Finances** | | | **Personality** | | | **Appearance** | | | **Mutual hobbies** | | | **Cooperative regarding one’s work** | | | **Skills/attitude towards chores/childrearing** | | | **Mean (SD) important** |
| --- | --- | --- | --- | --- | --- | --- | --- | --- | --- | --- | --- | --- | --- | --- | --- | --- | --- | --- | --- | --- | --- | --- | --- | --- | --- |
|  | Important | Would consider | p | Important | Would consider | p | Important | Would consider | p | Important | Would consider | p | Important | Would consider | p | Important | Would consider | p | Important | Would consider | p | Important | Would consider | p |  |
| Total | 11.7 | 44.4 |  | 32.0 | 54.4 |  | 42.7 | 51.4 |  | 89.1 | 9.9 |  | 17.7 | 61.8 |  | 26.1 | 51.1 |  | 54.8 | 41.1 |  | 62.9 | 35.0 |  | 3.4 (2) |
| ***Education*** |  |  |  |  |  |  |  |  |  |  |  |  |  |  |  |  |  |  |  |  |  |  |  |  |  |
| High school or less | 7.2 | 32.8 | < 0.001 | 23.2 | 54.7 | < 0.001 | 33.9 | 56.2 | < 0.001 | 84.5 | 13.0 | 0.001 | 18.5 | 59.9 | 0.711 | 25.7 | 47.5 | 0.274 | 46.3 | 47.6 | 0.002 | 61.2 | 36.0 | 0.694 | 3 (1.9) |
| Vocational school/ short college | 6.3 | 42.3 |  | 31.8 | 57.7 |  | 44.2 | 51.6 |  | 87.8 | 11.3 |  | 19.3 | 60.7 |  | 26.5 | 53.9 |  | 56.3 | 39.9 |  | 66.1 | 31.8 |  | 3.4 (1.9) |
| Undergraduate studies | 17.6 | 52.7 |  | 37.9 | 52.0 |  | 47.3 | 48.0 |  | 93.1 | 6.7 |  | 16.0 | 63.7 |  | 26.3 | 51.3 |  | 59.0 | 37.8 |  | 61.8 | 36.4 |  | 3.6 (2) |
| Graduate studies | 40.0 | 60.0 |  | 40.0 | 40.0 |  | 60.0 | 40.0 |  | 100.0 | 0 | 0 | 20.0 | 80.0 |  | 20.0 | 80.0 |  | 100.0 | 0 |  | 80.0 | 20.0 |  | 4.6 (2.3) |
| ***Occupational Status*** |  |  |  |  |  |  |  |  |  |  |  |  |  |  |  |  |  |  |  |  |  |  |  |  |  |
| Regular employee | 11.5 | 41.2 | < 0.001 | 32.1 | 55.8 | < 0.001 | 42.1 | 52.4 | 0.057 | 87.8 | 11.1 | 0.005 | 16.2 | 62.6 | 0.077 | 22.6 | 54.1 | 0.591 | 46.2 | 48.5 | < 0.001 | 61.8 | 35.9 | 0.279 | 3.2 (1.9) |
| Part-time/temporary worker | 7.3 | 32.1 |  | 23.3 | 56.5 |  | 40.2 | 49.0 |  | 86.5 | 10.9 |  | 19.7 | 63.2 |  | 29.5 | 49.2 |  | 50.5 | 43.2 |  | 62.2 | 34.2 |  | 3.2 (1.9) |
| Business owner/family business | 0 | 60.0 |  | 20.0 | 60.0 |  | 40.0 | 60.0 |  | 100.0 | 0 |  | 60.0 | 20.0 |  | 40.0 | 20.0 |  | 60.0 | 20.0 |  | 60.0 | 40.0 |  | 3.8 (1.9) |
| Unemployed | 0 | 33.9 |  | 22.0 | 50.8 |  | 32.2 | 62.7 |  | 79.7 | 20.3 |  | 13.6 | 54.2 |  | 22.0 | 52.5 |  | 42.4 | 52.5 |  | 53.4 | 46.6 |  | 2.6 (1.8) |
| Student | 14.7 | 53.3 |  | 36.7 | 53.3 |  | 45.5 | 50.0 |  | 92.9 | 6.6 |  | 17.6 | 62.9 |  | 26.4 | 50.6 |  | 65.4 | 32.8 |  | 65.8 | 32.9 |  | 3.6 (2) |
| ***Annual Income (in JPY 10,000s)*** |  |  |  |  |  |  |  |  |  |  |  |  |  |  |  |  |  |  |  |  |  |  |  |  |  |
| 0-99 | 12.7 | 47.0 | < 0.001 | 33.4 | 52.7 | 0.027 | 43.1 | 51.2 | 0.498 | 88.9 | 10.5 | 0.07 | 17.6 | 60.9 | 0.845 | 26.7 | 50.2 | 0.525 | 59.5 | 36.6 | 0.002 | 62.6 | 35.3 | 0.978 | 3.4 (2) |
| 100-199 | 7.8 | 31.9 |  | 21.7 | 65.1 |  | 38.0 | 54.2 |  | 86.7 | 10.2 |  | 17.5 | 63.9 |  | 27.1 | 47.6 |  | 46.4 | 48.2 |  | 63.9 | 33.7 |  | 3.1 (1.9) |
| 200-299 | 10.4 | 42.1 |  | 37.1 | 50.5 |  | 43.3 | 51.7 |  | 91.1 | 8.4 |  | 16.3 | 64.9 |  | 22.8 | 57.9 |  | 46.5 | 48.5 |  | 62.4 | 35.6 |  | 3.3 (1.9) |
| ≥300 | 13.3 | 57.3 |  | 33.3 | 58.7 |  | 50.7 | 46.7 |  | 92.0 | 8.0 |  | 21.3 | 61.3 |  | 28.0 | 50.7 |  | 49.3 | 49.3 |  | 68.0 | 30.7 |  | 3.6 (1.9) |
| **Preferred life course of wife** |  |  |  |  |  |  |  |  |  |  |  |  |  |  |  |  |  |  |  |  |  |  |  |  |  |
| Working | 12.0 | 48.4 | 0.026 | 31.0 | 57.4 | 0.182 | 41.6 | 52.4 | 0.803 | 88.0 | 11.0 | 0.471 | 16.3 | 61.8 | 0.418 | 26.2 | 51.7 | 0.841 | 65.4 | 33.0 | < 0.001 | 67.0 | 31.5 | 0.054 | 3.5 (2) |
| Homemaker | 11.1 | 41.7 |  | 32.7 | 52.7 |  | 43.3 | 51.3 |  | 89.9 | 9.0 |  | 18.5 | 62.1 |  | 26.3 | 50.3 |  | 48.1 | 46.1 |  | 60.5 | 37.0 |  | 3.3 (2) |
| ***Area of Residence: Population*** *Size* and Density |  |  |  |  |  |  |  |  |  |  |  |  |  |  |  |  |  |  |  |  |  |  |  |  |  |
| Non-densely inhabited area | 7.4 | 39.6 | 0.004 | 25.7 | 55.0 | 0.001 | 37.5 | 51.9 | < 0.001 | 83.4 | 13.9 | < 0.001 | 16.9 | 62.4 | 0.317 | 23.7 | 52.1 | 0.674 | 51.2 | 44.1 | 0.603 | 60.8 | 37.4 | 0.068 | 3.1 (1.9) |
| <200,000 | 14.2 | 44.2 |  | 37.3 | 53.6 |  | 45.9 | 51.7 |  | 91.5 | 7.9 |  | 16.1 | 64.2 |  | 28.5 | 48.5 |  | 57.3 | 39.1 |  | 68.1 | 29.5 |  | 3.6 (1.9) |
| 200,000 to <1,000,000 | 13.2 | 46.6 |  | 32.6 | 56.3 |  | 46.4 | 49.1 |  | 93.5 | 5.9 |  | 21.6 | 59.3 |  | 27.8 | 50.5 |  | 57.6 | 38.6 |  | 63.9 | 33.4 |  | 3.6 (2) |
| ≥1,000,000 | 11.7 | 48.0 |  | 32.8 | 51.4 |  | 40.1 | 53.8 |  | 87.0 | 13.0 |  | 15.0 | 61.5 |  | 23.9 | 54.3 |  | 52.2 | 43.3 |  | 57.1 | 41.7 |  | 3.2 (2) |

**STable 18.** Factors listed as “important” or “would consider” when choosing a partner among unmarried men with marriage intention, aged 18-24 years, in the National Fertility Survey 2015 by sociodemographic variables. Numbers are shown in percent.

|  | **Education** | | | **Occupation** | | | **Finances** | | | **Personality** | | | **Appearance** | | | **Mutual hobbies** | | | **Cooperative regarding one’s work** | | | **Skills/attitude towards chores/childrearing** | | | **Mean (SD) important** |
| --- | --- | --- | --- | --- | --- | --- | --- | --- | --- | --- | --- | --- | --- | --- | --- | --- | --- | --- | --- | --- | --- | --- | --- | --- | --- |
|  | Important | Would consider | p | Important | Would consider | p | Important | Would consider | p | Important | Would consider | p | Important | Would consider | p | Important | Would consider | p | Important | Would consider | p | Important | Would consider | p |  |
| Total | 3.7 | 30.0 |  | 6.2 | 45.0 |  | 4.9 | 39.0 |  | 79.7 | 17.4 |  | 26.9 | 60.2 |  | 22.1 | 55.1 |  | 46.6 | 45.3 |  | 49.2 | 45.7 |  | 2.4 (1.7) |
| ***Education*** |  |  |  |  |  |  |  |  |  |  |  |  |  |  |  |  |  |  |  |  |  |  |  |  |  |
| High school or less | 2.8 | 22.7 | < 0.001 | 5.6 | 38.2 | 0.005 | 5.2 | 37.0 | 0.463 | 76.2 | 20.2 | 0.005 | 26.1 | 60.6 | 0.867 | 21.1 | 55.5 | 0.107 | 41.5 | 49.6 | 0.01 | 50.7 | 44.4 | 0.679 | 2.3 (1.7) |
| Vocational school/ short college | 3.2 | 27.1 |  | 6.4 | 44.7 |  | 6.9 | 38.8 |  | 74.5 | 21.8 |  | 24.1 | 62.0 |  | 29.3 | 45.2 |  | 43.9 | 44.9 |  | 44.4 | 50.3 |  | 2.3 (1.8) |
| Undergraduate studies | 4.4 | 36.4 |  | 6.4 | 49.7 |  | 4.2 | 39.8 |  | 84.0 | 14.0 |  | 29.1 | 58.9 |  | 20.2 | 57.8 |  | 51.4 | 42.6 |  | 49.2 | 45.4 |  | 2.5 (1.6) |
| Graduate studies | 9.4 | 46.9 |  | 9.4 | 59.4 |  | 3.1 | 53.1 |  | 96.9 | 3.1 | 0 | 25.0 | 59.4 |  | 25.0 | 50.0 |  | 62.5 | 34.4 |  | 53.1 | 46.9 |  | 2.8 (1.6) |
| ***Occupational Status*** |  |  |  |  |  |  |  |  |  |  |  |  |  |  |  |  |  |  |  |  |  |  |  |  |  |
| Regular employee | 2.9 | 24.8 | < 0.001 | 5.4 | 44.6 | 0.392 | 3.4 | 41.9 | 0.275 | 75.2 | 20.4 | 0.002 | 27.1 | 59.4 | 0.517 | 19.4 | 56.6 | 0.358 | 44.9 | 45.8 | 0.001 | 47.3 | 46.6 | 0.652 | 2.3 (1.6) |
| Part-time/temporary worker | 6.0 | 20.0 |  | 7.0 | 35.0 |  | 6.0 | 40.0 |  | 72.0 | 24.0 |  | 27.0 | 59.0 |  | 22.0 | 54.0 |  | 38.0 | 43.0 |  | 49.0 | 46.0 |  | 2.3 (1.9) |
| Business owner/family business | 6.7 | 20.0 |  | 6.7 | 33.3 |  | 6.7 | 26.7 |  | 73.3 | 26.7 |  | 26.7 | 46.7 |  | 40.0 | 26.7 |  | 53.3 | 40.0 |  | 66.7 | 26.7 |  | 2.8 (2.4) |
| Unemployed | 0 | 21.4 |  | 4.8 | 45.2 |  | 11.9 | 40.5 |  | 82.9 | 14.6 |  | 16.7 | 64.3 |  | 21.4 | 52.4 |  | 43.9 | 51.2 |  | 55.0 | 37.5 |  | 2.3 (1.6) |
| Student | 3.9 | 37.0 |  | 6.6 | 48.2 |  | 5.0 | 37.2 |  | 85.3 | 13.3 |  | 27.0 | 61.7 |  | 23.4 | 55.9 |  | 49.8 | 44.9 |  | 49.5 | 46.5 |  | 2.5 (1.7) |
| ***Annual Income (in JPY 10,000s)*** |  |  |  |  |  |  |  |  |  |  |  |  |  |  |  |  |  |  |  |  |  |  |  |  |  |
| 0-99 | 3.8 | 34.3 | 0.001 | 6.7 | 47.6 | 0.008 | 5.5 | 38.6 | 0.336 | 82.2 | 15.8 | 0.027 | 27.0 | 60.8 | 0.17 | 22.1 | 55.9 | 0.569 | 47.5 | 45.9 | 0.006 | 50.2 | 45.0 | 0.205 | 2.4 (1.7) |
| 100-199 | 4.9 | 17.1 |  | 9.8 | 30.5 |  | 7.3 | 34.1 |  | 74.4 | 18.3 |  | 34.1 | 52.4 |  | 22.0 | 50.0 |  | 43.9 | 36.6 |  | 48.8 | 41.5 |  | 2.5 (2) |
| 200-299 | 3.2 | 21.5 |  | 2.5 | 40.5 |  | 1.3 | 41.8 |  | 73.2 | 22.3 |  | 23.7 | 57.7 |  | 17.1 | 57.0 |  | 42.4 | 48.7 |  | 46.2 | 48.1 |  | 2.1 (1.5) |
| ≥300 | 2.5 | 23.3 |  | 5.0 | 45.0 |  | 4.2 | 39.2 |  | 78.3 | 18.3 |  | 26.7 | 64.2 |  | 25.0 | 52.5 |  | 45.0 | 46.7 |  | 44.2 | 53.3 |  | 2.3 (1.7) |
| **Preferred life course of wife** |  |  |  |  |  |  |  |  |  |  |  |  |  |  |  |  |  |  |  |  |  |  |  |  |  |
| Working | 5.2 | 29.7 | 0.026 | 7.3 | 44.0 | 0.236 | 6.1 | 43.3 | 0.005 | 75.5 | 20.7 | 0.007 | 25.0 | 62.0 | 0.641 | 21.5 | 56.7 | 0.627 | 43.0 | 46.8 | 0.022 | 43.6 | 50.5 | 0.003 | 2.3 (1.9) |
| Homemaker | 2.2 | 31.4 |  | 5.0 | 47.3 |  | 3.4 | 37.0 |  | 82.8 | 15.3 |  | 27.4 | 59.4 |  | 21.9 | 54.1 |  | 49.9 | 43.5 |  | 54.1 | 41.5 |  | 2.5 (1.6) |
| ***Area of Residence: Population*** *Size* and Density |  |  |  |  |  |  |  |  |  |  |  |  |  |  |  |  |  |  |  |  |  |  |  |  |  |
| Non-densely inhabited area | 2.3 | 26.7 | 0.134 | 6.5 | 44.6 | 0.245 | 6.2 | 43.4 | 0.252 | 73.9 | 22.6 | 0.009 | 25.5 | 56.3 | 0.002 | 23.2 | 52.8 | 0.395 | 41.9 | 47.5 | 0.21 | 49.0 | 44.3 | 0.027 | 2.3 (1.8) |
| <200,000 | 3.3 | 29.4 |  | 7.2 | 39.1 |  | 4.7 | 36.2 |  | 78.0 | 18.1 |  | 22.7 | 62.9 |  | 18.3 | 59.9 |  | 48.2 | 45.3 |  | 52.7 | 43.3 |  | 2.3 (1.6) |
| 200,000 to <1,000,000 | 3.8 | 32.6 |  | 4.7 | 49.4 |  | 3.8 | 39.1 |  | 85.3 | 12.6 |  | 28.2 | 63.2 |  | 24.1 | 55.3 |  | 50.4 | 43.1 |  | 51.6 | 45.5 |  | 2.5 (1.6) |
| ≥1,000,000 | 6.2 | 31.8 |  | 7.1 | 46.2 |  | 4.7 | 35.5 |  | 82.5 | 16.1 |  | 32.5 | 57.9 |  | 22.3 | 52.1 |  | 45.7 | 45.2 |  | 41.0 | 51.4 |  | 2.4 (1.8) |

**STable 19.** Factors listed as “important” or “would consider” when choosing a partner among unmarried women with marriage intention, aged 25-49 years, in the National Fertility Survey 2015 by sociodemographic variables. Numbers are shown in percent.

|  | **Education** | | | **Occupation** | | | **Finances** | | | **Personality** | | | **Appearance** | | | **Mutual hobbies** | | | **Cooperative regarding one’s work** | | | **Skills/attitude towards chores/childrearing** | | | **Mean (SD) important** |
| --- | --- | --- | --- | --- | --- | --- | --- | --- | --- | --- | --- | --- | --- | --- | --- | --- | --- | --- | --- | --- | --- | --- | --- | --- | --- |
|  | Important | Would consider | p | Important | Would consider | p | Important | Would consider | p | Important | Would consider | p | Important | Would consider | p | Important | Would consider | p | Important | Would consider | p | Important | Would consider | p |  |
| Total | 8.0 | 44.4 |  | 25.4 | 58.5 |  | 35.3 | 58.7 |  | 88.4 | 10.9 |  | 12.5 | 64.4 |  | 21.2 | 51.9 |  | 41.6 | 48.9 |  | 48.6 | 47.0 |  | 2.8 (1.8) |
| ***Education*** |  |  |  |  |  |  |  |  |  |  |  |  |  |  |  |  |  |  |  |  |  |  |  |  |  |
| High school or less | 3.3 | 31.2 | < 0.001 | 21.1 | 56.5 | < 0.001 | 35.2 | 58.0 | 0.679 | 83.3 | 15.6 | < 0.001 | 11.1 | 61.7 | 0.046 | 20.4 | 51.2 | 0.653 | 34.9 | 52.3 | < 0.001 | 44.2 | 50.6 | 0.034 | 2.5 (1.7) |
| Vocational school/ short college | 5.3 | 47.6 |  | 24.0 | 61.0 |  | 33.9 | 61.1 |  | 89.3 | 10.0 |  | 12.1 | 65.6 |  | 23.1 | 52.2 |  | 44.4 | 47.4 |  | 49.2 | 46.6 |  | 2.8 (1.8) |
| Undergraduate studies | 16.3 | 53.7 |  | 32.0 | 57.3 |  | 36.8 | 57.1 |  | 93.3 | 6.5 |  | 15.0 | 65.6 |  | 19.5 | 52.9 |  | 44.6 | 48.1 |  | 51.4 | 44.5 |  | 3.1 (1.9) |
| Graduate studies | 20.7 | 72.4 |  | 37.9 | 58.6 |  | 41.4 | 51.7 |  | 86.2 | 13.8 |  | 10.3 | 72.4 |  | 24.1 | 48.3 |  | 62.1 | 27.6 |  | 72.4 | 27.6 |  | 3.6 (1.9) |
| ***Occupational Status*** |  |  |  |  |  |  |  |  |  |  |  |  |  |  |  |  |  |  |  |  |  |  |  |  |  |
| Regular employee | 9.1 | 49.2 | < 0.001 | 28.8 | 57.8 | < 0.001 | 37.3 | 58.3 | 0.011 | 89.8 | 9.7 | 0.013 | 12.9 | 65.8 | 0.008 | 19.7 | 51.7 | 0.481 | 44.7 | 48.2 | < 0.001 | 51.8 | 45.3 | 0.001 | 2.9 (1.8) |
| Part-time/temporary worker | 7.6 | 37.2 |  | 24.5 | 57.0 |  | 35.5 | 57.7 |  | 88.8 | 10.8 |  | 11.1 | 64.2 |  | 23.8 | 52.3 |  | 36.8 | 51.6 |  | 46.4 | 48.4 |  | 2.7 (1.8) |
| Business owner/family business | 3.3 | 40.0 |  | 13.3 | 68.3 |  | 30.0 | 61.7 |  | 91.7 | 6.7 |  | 25.0 | 53.3 |  | 21.7 | 58.3 |  | 66.7 | 26.7 |  | 45.0 | 46.7 |  | 3 (1.7) |
| Unemployed | 3.5 | 41.0 |  | 14.0 | 64.0 |  | 27.3 | 64.5 |  | 80.5 | 17.2 |  | 8.8 | 65.5 |  | 20.2 | 52.6 |  | 28.1 | 55.0 |  | 35.5 | 56.2 |  | 2.2 (1.8) |
| Student | 15.4 | 46.2 |  | 23.1 | 61.5 |  | 23.1 | 53.8 |  | 92.3 | 7.7 |  | 23.1 | 30.8 |  | 15.4 | 53.8 |  | 61.5 | 38.5 |  | 61.5 | 38.5 |  | 3.2 (2) |
| ***Annual Income (in JPY 10,000s)*** |  |  |  |  |  |  |  |  |  |  |  |  |  |  |  |  |  |  |  |  |  |  |  |  |  |
| 0-99 | 4.5 | 39.9 | < 0.001 | 17.3 | 61.3 | 0.001 | 28.8 | 61.9 | 0.053 | 83.6 | 14.9 | 0.09 | 11.0 | 62.0 | 0.23 | 21.8 | 52.1 | 0.853 | 36.4 | 48.7 | < 0.001 | 43.5 | 48.1 | 0.012 | 2.5 (1.8) |
| 100-199 | 6.2 | 37.8 |  | 26.9 | 55.6 |  | 35.4 | 58.7 |  | 89.8 | 9.6 |  | 13.4 | 63.5 |  | 23.9 | 49.1 |  | 38.1 | 52.0 |  | 49.2 | 48.4 |  | 2.8 (1.8) |
| 200-299 | 8.4 | 42.4 |  | 27.5 | 57.0 |  | 38.0 | 57.8 |  | 89.0 | 10.4 |  | 13.1 | 62.4 |  | 20.1 | 51.8 |  | 41.0 | 50.2 |  | 48.8 | 47.4 |  | 2.9 (1.8) |
| 300-399 | 11.4 | 52.2 |  | 28.9 | 58.9 |  | 38.2 | 56.9 |  | 90.4 | 8.8 |  | 10.9 | 70.1 |  | 21.3 | 53.4 |  | 47.5 | 45.2 |  | 52.2 | 44.0 |  | 3 (1.9) |
| 400-499 | 12.6 | 52.0 |  | 29.9 | 57.5 |  | 37.0 | 56.7 |  | 88.2 | 11.8 |  | 16.7 | 65.1 |  | 15.7 | 54.3 |  | 46.5 | 49.6 |  | 52.8 | 44.1 |  | 3 (2) |
| ≥500 | 8.7 | 64.1 |  | 26.9 | 63.4 |  | 32.3 | 60.2 |  | 93.5 | 6.5 |  | 10.8 | 66.7 |  | 22.6 | 49.5 |  | 51.6 | 44.1 |  | 44.1 | 51.6 |  | 2.9 (1.7) |
| **Preferred life course of wife** |  |  |  |  |  |  |  |  |  |  |  |  |  |  |  |  |  |  |  |  |  |  |  |  |  |
| Working | 8.5 | 46.3 | 0.26 | 25.7 | 58.2 | 0.841 | 33.0 | 60.5 | 0.027 | 88.5 | 11.1 | 0.606 | 13.2 | 65.4 | 0.565 | 21.8 | 53.6 | 0.089 | 50.9 | 44.6 | < 0.001 | 50.6 | 45.1 | 0.448 | 2.9 (1.9) |
| Homemaker | 7.6 | 43.4 |  | 26.2 | 58.8 |  | 37.9 | 57.7 |  | 90.0 | 9.7 |  | 11.7 | 65.6 |  | 20.0 | 50.8 |  | 34.6 | 52.2 |  | 48.0 | 48.1 |  | 2.8 (1.8) |
| ***Area of Residence: Population*** *Size* and Density |  |  |  |  |  |  |  |  |  |  |  |  |  |  |  |  |  |  |  |  |  |  |  |  |  |
| Non-densely inhabited area | 6.3 | 39.4 | 0.004 | 24.4 | 57.8 | 0.386 | 37.4 | 58.3 | 0.307 | 85.7 | 13.8 | 0.015 | 12.8 | 64.4 | 0.465 | 19.1 | 52.5 | 0.283 | 44.4 | 47.1 | 0.403 | 49.2 | 47.1 | 0.575 | 2.8 (1.8) |
| <200,000 | 6.3 | 45.3 |  | 24.1 | 59.8 |  | 33.3 | 60.5 |  | 87.2 | 11.1 |  | 14.7 | 61.0 |  | 19.5 | 51.1 |  | 37.1 | 53.0 |  | 50.2 | 45.2 |  | 2.7 (1.8) |
| 200,000 to <1,000,000 | 10.6 | 45.9 |  | 28.6 | 55.6 |  | 36.5 | 56.2 |  | 88.9 | 10.3 |  | 11.5 | 64.4 |  | 21.6 | 52.2 |  | 41.5 | 48.1 |  | 47.2 | 49.0 |  | 2.9 (1.9) |
| ≥1,000,000 | 8.2 | 47.4 |  | 23.7 | 61.8 |  | 32.9 | 60.8 |  | 92.0 | 7.7 |  | 11.3 | 68.0 |  | 24.9 | 51.4 |  | 43.0 | 48.0 |  | 48.1 | 45.9 |  | 2.8 (1.8) |

**STable 20.** Factors listed as “important” or “would consider” when choosing a partner among unmarried men with marriage intention, aged 25-49 years, in the National Fertility Survey 2015 by sociodemographic variables. Numbers are shown in percent.

|  | **Education** | | | **Occupation** | | | **Finances** | | | **Personality** | | | **Appearance** | | | **Mutual hobbies** | | | **Cooperative regarding one’s work** | | | **Skills/attitude towards chores/childrearing** | | | **Mean (SD) important** |
| --- | --- | --- | --- | --- | --- | --- | --- | --- | --- | --- | --- | --- | --- | --- | --- | --- | --- | --- | --- | --- | --- | --- | --- | --- | --- |
|  | Important | Would consider | p | Important | Would consider | p | Important | Would consider | p | Important | Would consider | p | Important | Would consider | p | Important | Would consider | p | Important | Would consider | p | Important | Would consider | p |  |
| Total | 2.7 | 23.2 |  | 4.9 | 35.0 |  | 4.0 | 34.7 |  | 72.6 | 23.5 |  | 20.2 | 62.2 |  | 17.5 | 52.1 |  | 35.3 | 52.7 |  | 41.0 | 52.5 |  | 2 (1.6) |
| ***Education*** |  |  |  |  |  |  |  |  |  |  |  |  |  |  |  |  |  |  |  |  |  |  |  |  |  |
| High school or less | 1.5 | 14.3 | < 0.001 | 3.8 | 26.1 | < 0.001 | 4.0 | 30.1 | 0.013 | 65.8 | 29.3 | < 0.001 | 17.8 | 59.5 | < 0.001 | 14.7 | 52.2 | 0.133 | 30.5 | 55.8 | 0.001 | 40.2 | 53.1 | 0.074 | 1.8 (1.5) |
| Vocational school/ short college | 1.5 | 22.7 |  | 4.5 | 33.3 |  | 3.8 | 35.2 |  | 72.9 | 22.3 |  | 17.2 | 65.9 |  | 19.1 | 53.9 |  | 35.1 | 52.8 |  | 38.1 | 53.0 |  | 1.9 (1.5) |
| Undergraduate studies | 3.5 | 31.5 |  | 6.0 | 43.5 |  | 4.1 | 39.2 |  | 79.1 | 18.4 |  | 23.8 | 62.6 |  | 18.7 | 51.8 |  | 38.3 | 50.8 |  | 41.6 | 52.6 |  | 2.1 (1.6) |
| Graduate studies | 10.2 | 42.2 |  | 7.8 | 53.9 |  | 3.9 | 39.8 |  | 79.7 | 18.8 |  | 25.0 | 69.5 |  | 20.3 | 49.2 |  | 48.4 | 44.5 |  | 50.0 | 47.7 |  | 2.5 (1.6) |
| ***Occupational Status*** |  |  |  |  |  |  |  |  |  |  |  |  |  |  |  |  |  |  |  |  |  |  |  |  |  |
| Regular employee | 3.2 | 24.8 | 0.022 | 5.2 | 37.8 | 0.048 | 4.0 | 35.6 | 0.059 | 72.8 | 23.4 | 0.826 | 20.4 | 62.7 | 0.587 | 16.8 | 54.1 | 0.379 | 34.5 | 55.6 | < 0.001 | 41.4 | 53.0 | 0.146 | 2 (1.6) |
| Part-time/temporary worker | 1.5 | 23.3 |  | 4.2 | 31.2 |  | 3.9 | 35.4 |  | 7.4 | 20.0 |  | 18.8 | 64.8 |  | 20.1 | 52.0 |  | 31.5 | 53.6 |  | 41.2 | 52.7 |  | 2 (1.6) |
| Business owner/family business | 1.1 | 15.5 |  | 2.9 | 28.7 |  | 2.3 | 24.1 |  | 71.6 | 25.0 |  | 21.8 | 64.9 |  | 17.1 | 48.0 |  | 51.1 | 38.6 |  | 45.3 | 44.8 |  | 2.1 (1.5) |
| Unemployed | 2.2 | 21.9 |  | 4.5 | 31.5 |  | 5.6 | 36.0 |  | 72.5 | 24.2 |  | 20.2 | 57.3 |  | 16.9 | 49.4 |  | 30.7 | 48.9 |  | 36.3 | 54.7 |  | 1.9 (1.5) |
| Student | 9.1 | 31.8 |  | 9.1 | 40.9 |  | 0 | 45.5 |  | 73.9 | 17.4 |  | 18.2 | 63.6 |  | 22.7 | 36.4 |  | 40.9 | 50.0 |  | 43.5 | 43.5 |  | 2.1 (1.8) |
| ***Annual Income (in JPY 10,000s)*** |  |  |  |  |  |  |  |  |  |  |  |  |  |  |  |  |  |  |  |  |  |  |  |  |  |
| 0-99 | 2.7 | 22.3 | 0.015 | 5.6 | 32.6 | 0.158 | 5.6 | 37.3 | 0.191 | 71.4 | 24.0 | 0.427 | 20.8 | 57.1 | < 0.001 | 19.9 | 45.9 | 0.043 | 36.2 | 46.2 | 0.005 | 38.2 | 53.7 | 0.354 | 2 (1.7) |
| 100-199 | 1.1 | 19.4 |  | 5.0 | 28.9 |  | 5.6 | 33.3 |  | 70.0 | 27.2 |  | 13.3 | 66.1 |  | 16.7 | 50.6 |  | 27.9 | 58.7 |  | 42.7 | 47.8 |  | 1.8 (1.6) |
| 200-299 | 2.5 | 18.2 |  | 5.2 | 30.3 |  | 3.2 | 33.6 |  | 71.7 | 22.3 |  | 22.1 | 57.0 |  | 17.1 | 53.8 |  | 36.7 | 51.1 |  | 44.8 | 48.5 |  | 2 (1.6) |
| 300-399 | 3.1 | 23.9 |  | 4.2 | 38.4 |  | 3.9 | 37.7 |  | 72.3 | 24.2 |  | 20.4 | 62.1 |  | 19.3 | 54.4 |  | 35.4 | 54.3 |  | 40.3 | 53.6 |  | 2 (1.6) |
| 400-499 | 3.3 | 27.8 |  | 6.0 | 38.2 |  | 3.3 | 32.7 |  | 76.4 | 20.6 |  | 21.2 | 68.1 |  | 16.7 | 52.2 |  | 35.9 | 53.3 |  | 40.5 | 54.4 |  | 2 (1.6) |
| 500-599 | 1.7 | 27.5 |  | 3.4 | 39.1 |  | 2.1 | 33.0 |  | 72.5 | 24.9 |  | 16.7 | 69.1 |  | 12.1 | 58.2 |  | 33.0 | 58.8 |  | 38.4 | 57.3 |  | 1.8 (1.4) |
| 600-699 | 7.1 | 32.1 |  | 5.4 | 41.1 |  | 3.6 | 23.2 |  | 78.6 | 17.9 |  | 30.4 | 64.3 |  | 21.4 | 37.5 |  | 42.9 | 51.8 |  | 39.3 | 53.6 |  |  |
| ≥700 |  |  |  |  |  |  |  |  |  |  |  |  |  |  |  |  |  |  |  |  |  |  |  |  |  |
| **Preferred life course of wife** |  |  |  |  |  |  |  |  |  |  |  |  |  |  |  |  |  |  |  |  |  |  |  |  |  |
| Working | 3.2 | 24.8 | 0.242 | 6.4 | 35.9 | 0.035 | 5.5 | 38.0 | < 0.001 | 68.9 | 26.2 | < 0.001 | 18.8 | 60.9 | 0.001 | 16.9 | 52.6 | 0.689 | 34.2 | 52.7 | 0.007 | 34.9 | 56.8 | < 0.001 | 1.9 (1.7) |
| Homemaker | 2.2 | 22.9 |  | 3.8 | 36.8 |  | 2.6 | 33.6 |  | 77.6 | 20.4 |  | 22.4 | 63.5 |  | 18.0 | 53.1 |  | 37.4 | 53.8 |  | 47.9 | 48.8 |  | 2.1 (1.5) |
| ***Area of Residence: Population*** *Size* and Density |  |  |  |  |  |  |  |  |  |  |  |  |  |  |  |  |  |  |  |  |  |  |  |  |  |
| Non-densely inhabited area | 2.5 | 20.8 | 0.352 | 3.9 | 34.9 | 0.161 | 4.0 | 34.2 | 0.012 | 65.8 | 28.9 | < 0.001 | 16.3 | 64.3 | 0.019 | 16.1 | 53.9 | 0.462 | 34.4 | 53.3 | 0.287 | 38.8 | 53.8 | 0.105 | 1.8 (1.6) |
| <200,000 | 2.7 | 21.8 |  | 5.0 | 31.4 |  | 3.1 | 33.5 |  | 71.5 | 24.4 |  | 18.9 | 65.1 |  | 17.0 | 49.8 |  | 31.3 | 55.7 |  | 38.8 | 53.5 |  | 1.9 (1.5) |
| 200,000 to <1,000,000 | 3.2 | 25.4 |  | 5.9 | 38.5 |  | 5.4 | 39.4 |  | 75.9 | 20.7 |  | 22.5 | 59.7 |  | 17.2 | 53.2 |  | 36.6 | 51.9 |  | 45.8 | 48.5 |  | 2.1 (1.6) |
| ≥1,000,000 | 2.3 | 25.6 |  | 5.1 | 34.6 |  | 3.0 | 30.5 |  | 79.4 | 18.0 |  | 24.5 | 59.1 |  | 20.4 | 50.5 |  | 39.4 | 49.8 |  | 40.4 | 54.5 |  | 2.1 (1.6) |

**STable 21.** Factors listed as “important” or “would consider” when choosing a partner among never-married women with marriage intention, aged 18-49 years, in the National Fertility Survey 2015 by sociodemographic variables. Numbers are shown in percent.

|  | **Education** | | | **Occupation** | | | **Finances** | | | **Personality** | | | **Appearance** | | | **Mutual hobbies** | | | **Cooperative regarding one’s work** | | | **Skills/attitude towards chores/childrearing** | | | **Mean (SD) important** |
| --- | --- | --- | --- | --- | --- | --- | --- | --- | --- | --- | --- | --- | --- | --- | --- | --- | --- | --- | --- | --- | --- | --- | --- | --- | --- |
|  | Important | Would consider | p | Important | Would consider | p | Important | Would consider | p | Important | Would consider | p | Important | Would consider | p | Important | Would consider | p | Important | Would consider | p | Important | Would consider | p |  |
| Total | 10.1 | 45.4 |  | 29.2 | 56.5 |  | 38.6 | 55.4 |  | 89.2 | 10.1 |  | 15.3 | 63.1 |  | 23.4 | 51.4 |  | 47.6 | 45.6 |  | 54.8 | 42.0 |  | 3.3 (2.1) |
| **Age group** |  |  | 0.255 |  |  | 0.003 |  |  | 0.001 |  |  | 0.027 |  |  | 0.073 |  |  | < 0.001 |  |  | < 0.001 |  |  | < 0.001 |  |
| 18-24 | 11.5 | 44.8 |  | 32.2 | 54.5 |  | 42.9 | 51.2 |  | 89.7 | 9.3 |  | 17.6 | 61.6 |  | 26.3 | 50.9 |  | 55.1 | 40.7 |  | 63.1 | 34.8 |  | 3.6 (2.2) |
| 25-29 | 10.7 | 45.2 |  | 30.2 | 57.4 |  | 38.7 | 56.7 |  | 90.7 | 9.2 |  | 13.1 | 64.5 |  | 21.9 | 51.7 |  | 45.3 | 47.9 |  | 54.5 | 43.2 |  | 3.2 (2.1) |
| 30-34 | 8.2 | 43.0 |  | 24.0 | 60.1 |  | 34.0 | 60.1 |  | 86.0 | 13.5 |  | 16.2 | 63.2 |  | 21.1 | 54.7 |  | 41.9 | 49.9 |  | 49.4 | 45.9 |  | 3.0 (2.0) |
| 34-39 | 6.9 | 47.4 |  | 26.7 | 55.2 |  | 30.2 | 62.1 |  | 87.1 | 12.1 |  | 11.3 | 65.2 |  | 14.7 | 48.3 |  | 32.3 | 54.7 |  | 35.8 | 57.3 |  | 2.6 (1.9) |
| 40-44 | 6.6 | 49.1 |  | 24.0 | 56.3 |  | 33.9 | 58.3 |  | 85.1 | 14.3 |  | 9.6 | 68.9 |  | 22.0 | 47.6 |  | 32.7 | 53.6 |  | 38.6 | 57.2 |  | 2.7 (2.0) |
| 45-49 | 8.0 | 50.0 |  | 17.9 | 65.2 |  | 28.6 | 62.5 |  | 95.5 | 3.6 |  | 16.1 | 58.0 |  | 26.8 | 57.1 |  | 49.1 | 42.9 |  | 43.2 | 48.6 |  | 2.9 (1.9) |
| ***Education*** |  |  | < 0.001 |  |  | < 0.001 |  |  | 0.001 |  |  | < 0.001 |  |  | 0.357 |  |  | 0.648 |  |  | < 0.001 |  |  | 0.005 |  |
| High school or less | 5.1 | 32.5 |  | 22.5 | 56 |  | 33.8 | 57.7 |  | 83.9 | 14.5 |  | 15.2 | 60.3 |  | 22.8 | 49.6 |  | 39.8 | 50.9 |  | 50.0 | 45.9 |  | 2.9 (2.1) |
| Vocational school/ short college | 5.9 | 46.0 |  | 27.5 | 59.1 |  | 38.3 | 56.9 |  | 89.1 | 10.4 |  | 15.5 | 63.7 |  | 24.3 | 52.2 |  | 48.1 | 45.0 |  | 55.3 | 41.4 |  | 3.3 (2.1) |
| Undergraduate studies | 17.0 | 53.4 |  | 35.5 | 54.3 |  | 42.2 | 52.5 |  | 93.4 | 6.4 |  | 15.2 | 64.6 |  | 22.9 | 51.9 |  | 52.3 | 42.7 |  | 57.0 | 40.2 |  | 3.5 (2.1) |
| Graduate studies | 25.8 | 67.7 |  | 38.7 | 54.8 |  | 45.2 | 48.4 |  | 93.5 | 6.5 |  | 12.9 | 71.0 |  | 25.8 | 51.6 |  | 71.0 | 22.6 |  | 80.6 | 19.4 |  | 4.1 (2.0) |
| ***Occupational Status*** |  |  | < 0.001 |  |  | < 0.001 |  |  | 0.002 |  |  | 0.001 |  |  | 0.013 |  |  | 0.012 |  |  | < 0.001 |  |  | < 0.001 |  |
| Regular employee | 10.4 | 47.2 |  | 30.5 | 56.9 |  | 38.6 | 56.5 |  | 89.5 | 10.1 |  | 14.1 | 65.0 |  | 19.9 | 52.3 |  | 44.1 | 49.4 |  | 54.4 | 42.9 |  | 3.2 (2.1) |
| Part-time/temporary worker | 8.4 | 35.4 |  | 24.9 | 56.7 |  | 36.4 | 55.2 |  | 88.6 | 10.4 |  | 15.2 | 63.2 |  | 26.9 | 50.8 |  | 41.7 | 48.7 |  | 51.0 | 44.4 |  | 3.1 (2) |
| Business owner/family business | 2.0 | 43.1 |  | 15.7 | 66.7 |  | 31.4 | 60.8 |  | 90.2 | 7.8 |  | 29.4 | 49.0 |  | 21.6 | 58.8 |  | 66.7 | 25.5 |  | 49.0 | 45.1 |  | 3.2 (1.9) |
| Unemployed | 3.1 | 42.1 |  | 18.6 | 59.8 |  | 31.4 | 61.3 |  | 81.1 | 17.3 |  | 10.4 | 62.2 |  | 23.1 | 51.3 |  | 35.1 | 52.6 |  | 41.7 | 52.1 |  | 2.6 (2) |
| Student | 14.2 | 53.6 |  | 36.2 | 54.0 |  | 44.9 | 50.5 |  | 93.5 | 5.9 |  | 17.8 | 61.9 |  | 26.2 | 50.4 |  | 65.9 | 32.4 |  | 66.0 | 32.6 |  | 3.9 (2.1) |
| ***Annual Income (in JPY 10,000s)*** |  |  | < 0.001 |  |  | 0.567 |  |  | 0.585 |  |  | 0.203 |  |  | 0.254 |  |  | 0.061 |  |  | < 0.001 |  |  | 0.304 |  |
| 0-99 | 10.6 | 46.5 |  | 29.7 | 55.3 |  | 39.5 | 54.0 |  | 87.9 | 11.3 |  | 16.2 | 61.0 |  | 26.0 | 50.3 |  | 54.4 | 39.3 |  | 57.7 | 38.8 |  | 3.4 (2.2) |
| 100-199 | 7.4 | 36.9 |  | 26.1 | 58.9 |  | 36.1 | 57.2 |  | 89.0 | 9.6 |  | 16.9 | 61.6 |  | 25.8 | 48.1 |  | 40.7 | 51.0 |  | 54.0 | 43.5 |  | 3.1 (2) |
| 200-299 | 9.8 | 42.3 |  | 31.6 | 54.2 |  | 39.8 | 55.4 |  | 90.1 | 9.4 |  | 14.0 | 63.8 |  | 20.5 | 53.4 |  | 42.2 | 50.0 |  | 52.6 | 44.0 |  | 3.2 (2) |
| 300-399 | 11.9 | 53.3 |  | 29.7 | 58.9 |  | 39.7 | 55.8 |  | 91.6 | 8.4 |  | 12.8 | 69.0 |  | 21.1 | 53.6 |  | 46.9 | 47.2 |  | 54.2 | 42.2 |  | 3.3 (2) |
| 400-499 | 13.0 | 53.7 |  | 30.1 | 57.7 |  | 35.0 | 58.5 |  | 87.8 | 12.2 |  | 17.2 | 64.8 |  | 14.6 | 55.3 |  | 44.7 | 51.2 |  | 52.8 | 45.5 |  | 3.1 (2.2) |
| ≥500 | 9.4 | 62.4 |  | 28.2 | 61.2 |  | 31.8 | 61.2 |  | 94.1 | 5.9 |  | 9.4 | 67.1 |  | 23.5 | 45.9 |  | 50.6 | 44.7 |  | 45.9 | 51.8 |  | 3.1 (1.9) |
| **Preferred life course of wife** |  |  | 0.03 |  |  | 0.507 |  |  | 0.036 |  |  | 0.308 |  |  | 0.897 |  |  | 0.19 |  |  | < 0.001 |  |  | 0.059 |  |
| Working | 10.2 | 48.2 |  | 28.4 | 57.8 |  | 36.6 | 56.8 |  | 88.6 | 10.8 |  | 14.8 | 63.8 |  | 23.4 | 52.9 |  | 57.9 | 39.3 |  | 58.1 | 39.0 |  | 3.4 (2.1) |
| Homemaker | 9.9 | 43.5 |  | 30.3 | 55.6 |  | 40.4 | 54.7 |  | 90.2 | 9.0 |  | 15.4 | 63.6 |  | 23.2 | 50.1 |  | 40.6 | 49.9 |  | 53.5 | 43.4 |  | 3.2 (2.1) |
| **Region of residence** |  |  | 0.005 |  |  | 0.442 |  |  | 0.07 |  |  | 0.048 |  |  | 0.752 |  |  | 0.045 |  |  | 0.346 |  |  | 0.342 |  |
| Hokkaido | 7.9 | 39.3 |  | 25.8 | 53.9 |  | 33.7 | 60.7 |  | 84.3 | 12.4 |  | 19.1 | 55.1 |  | 28.1 | 46.1 |  | 41.6 | 53.9 |  | 46.1 | 48.3 |  | 3.0 (2.1) |
| Tohoku | 6.3 | 41.1 |  | 26.8 | 53.2 |  | 35.3 | 55.3 |  | 86.8 | 12.6 |  | 13.7 | 61.6 |  | 20.0 | 48.4 |  | 48.4 | 43.2 |  | 53.4 | 42.3 |  | 3.1 (2.1) |
| Kanto | 12.2 | 48.3 |  | 31.0 | 56.1 |  | 40.2 | 53.3 |  | 90.5 | 9.0 |  | 15.8 | 64.2 |  | 26.7 | 49.8 |  | 50.6 | 43.4 |  | 56.4 | 40.3 |  | 3.4 (2.1) |
| Chubu | 8.2 | 44.3 |  | 27.4 | 58.6 |  | 34.4 | 60.6 |  | 86.7 | 12.5 |  | 13.9 | 62.4 |  | 23.7 | 52.7 |  | 47.8 | 45.4 |  | 54.0 | 42.5 |  | 3.1 (2.1) |
| Kinki | 9.7 | 47.5 |  | 28.5 | 57.7 |  | 38.1 | 57.6 |  | 91.1 | 8.3 |  | 14.8 | 64.6 |  | 19.6 | 55.3 |  | 43.8 | 49.3 |  | 51.1 | 46.2 |  | 3.1 (2.0) |
| Chugoku | 10.0 | 40.0 |  | 30.0 | 54 |  | 41.5 | 51.0 |  | 91.5 | 7.5 |  | 16.0 | 64.0 |  | 19.0 | 51.5 |  | 43.5 | 47.5 |  | 54.5 | 42.5 |  | 3.3 (2.1) |
| Kyushu | 9.7 | 42.1 |  | 29.5 | 56.7 |  | 42.8 | 51.8 |  | 87.2 | 12.1 |  | 16.2 | 60.9 |  | 21.5 | 51.3 |  | 47.7 | 45.0 |  | 60.5 | 36.8 |  | 3.3 (2.2) |
| ***Area of Residence: Population*** *Size* and Density |  |  | < 0.001 |  |  | 0.151 |  |  | 0.129 |  |  | < 0.001 |  |  | 0.736 |  |  | 0.34 |  |  | 0.997 |  |  | 0.253 |  |
| Non-densely inhabited area | 7.2 | 40.7 |  | 26.4 | 56.3 |  | 37.1 | 55.6 |  | 84.7 | 13.9 |  | 16.1 | 62.9 |  | 21.3 | 51.6 |  | 47.6 | 45.8 |  | 54.6 | 42.7 |  | 3.1 (2.1) |
| <200,000 | 10.1 | 45.9 |  | 30.4 | 56.8 |  | 39.7 | 55.8 |  | 89.9 | 9.2 |  | 15.8 | 62.7 |  | 24.4 | 48.9 |  | 47.4 | 46.1 |  | 58.6 | 38.3 |  | 3.4 (2.1) |
| 200,000 to <1,000,000 | 12.5 | 46.3 |  | 31.1 | 55.7 |  | 41.0 | 52.8 |  | 91.3 | 8.2 |  | 15.4 | 61.7 |  | 23.5 | 52.1 |  | 48.5 | 44.6 |  | 54.3 | 42.1 |  | 3.4 (2.1) |
| ≥1,000,000 | 10.1 | 49.1 |  | 28.5 | 57.4 |  | 35.9 | 58.5 |  | 90.9 | 9.1 |  | 13.5 | 65.8 |  | 24.6 | 53.0 |  | 46.8 | 46.2 |  | 51.4 | 45.0 |  | 3.2 (2.1) |

**STable 22.** Factors listed as “important” or “would consider” when choosing a partner among never-married men with marriage intention, aged 18-49 years, in the National Fertility Survey 2015 by sociodemographic variables. Numbers are shown in percent.

|  | **Education** | | | **Occupation** | | | **Finances** | | | **Personality** | | | **Appearance** | | | **Mutual hobbies** | | | **Cooperative regarding one’s work** | | | **Skills/attitude towards chores/childrearing** | | | **Mean (SD) important** |
| --- | --- | --- | --- | --- | --- | --- | --- | --- | --- | --- | --- | --- | --- | --- | --- | --- | --- | --- | --- | --- | --- | --- | --- | --- | --- |
|  | Important | Would consider | p | Important | Would consider | p | Important | Would consider | p | Important | Would consider | p | Important | Would consider | p | Important | Would consider | p | Important | Would consider | p | Important | Would consider | p |  |
| Total | 3.1 | 25.8 |  | 5.3 | 39.4 |  | 4.3 | 36.6 |  | 75.5 | 21.2 |  | 22.6 | 61.4 |  | 19.1 | 53.5 |  | 39.3 | 50.0 |  | 43.9 | 50.1 |  | 2.1 (1.6) |
| **Age group** |  |  | < 0.001 |  |  | < 0.001 |  |  | < 0.001 |  |  | < 0.001 |  |  | < 0.001 |  |  | < 0.001 |  |  | < 0.001 |  |  | < 0.001 |  |
| 18-24 | 3.6 | 30.0 |  | 5.9 | 45.4 |  | 4.7 | 38.8 |  | 79.8 | 17.5 |  | 27.0 | 60.3 |  | 21.9 | 55.2 |  | 46.6 | 45.3 |  | 49.1 | 45.7 |  | 2.4 (1.7) |
| 25-29 | 5.4 | 24.8 |  | 8.4 | 41.0 |  | 6.1 | 40.2 |  | 75.9 | 21.3 |  | 24.8 | 61.6 |  | 22.7 | 51.9 |  | 40.7 | 49.0 |  | 49.5 | 46.5 |  | 2.3 (1.7) |
| 30-34 | 1.7 | 24.6 |  | 3.8 | 36.6 |  | 3.2 | 33.3 |  | 77.1 | 19.1 |  | 18.9 | 63.6 |  | 17.9 | 51.5 |  | 36.4 | 50.5 |  | 40.0 | 53.7 |  | 2.0 (1.5) |
| 34-39 | 0.9 | 21.7 |  | 2.4 | 33.1 |  | 2.7 | 34.6 |  | 67.0 | 30.0 |  | 17.5 | 62.3 |  | 15.4 | 50.9 |  | 32.8 | 54.8 |  | 36.3 | 55.0 |  | 1.7 (1.4) |
| 40-44 | 1.1 | 19.0 |  | 3.0 | 30.0 |  | 2.7 | 28.2 |  | 68.9 | 27.3 |  | 13.3 | 63.9 |  | 9.5 | 59.5 |  | 25.6 | 61.1 |  | 30.3 | 60.9 |  | 1.5 (1.4) |
| 45-49 | 2.7 | 22.2 |  | 2.7 | 29.2 |  | 3.2 | 34.6 |  | 67.0 | 25.4 |  | 19.5 | 55.7 |  | 13.0 | 49.5 |  | 28.3 | 57.1 |  | 34.8 | 56.0 |  | 1.7 (1.5) |
| ***Education*** |  |  | < 0.001 |  |  | < 0.001 |  |  | 0.009 |  |  | < 0.001 |  |  | < 0.001 |  |  | 0.078 |  |  | < 0.001 |  |  | 0.069 |  |
| High school or less | 1.8 | 16.7 |  | 4.2 | 30.8 |  | 4.4 | 32.6 |  | 69.2 | 26.7 |  | 20.0 | 60.0 |  | 16.7 | 53.6 |  | 33.5 | 54.0 |  | 43.3 | 50.4 |  | 1.9 (1.6) |
| Vocational school/ short college | 2.2 | 24.2 |  | 5.2 | 38.0 |  | 5.0 | 36.7 |  | 73.9 | 21.9 |  | 19.8 | 63.4 |  | 22.5 | 52.1 |  | 38.1 | 50.1 |  | 41.1 | 50.9 |  | 2.1 (1.6) |
| Undergraduate studies | 4.1 | 33.4 |  | 6.0 | 46.5 |  | 4.0 | 39.7 |  | 81.3 | 16.5 |  | 26.1 | 61.2 |  | 19.2 | 54.3 |  | 43.8 | 47.5 |  | 44.5 | 50.1 |  | 2.3 (1.6) |
| Graduate studies | 10.3 | 43.2 |  | 8.4 | 55.5 |  | 3.9 | 43.9 |  | 83.9 | 14.8 |  | 25.8 | 66.5 |  | 21.3 | 50.3 |  | 51.6 | 41.9 |  | 51.0 | 47.1 |  | 2.6 (1.6) |
| ***Occupational Status*** |  |  | < 0.001 |  |  | < 0.001 |  |  | 0.049 |  |  | < 0.001 |  |  | 0.004 |  |  | 0.004 |  |  | < 0.001 |  |  | 0.02 |  |
| Regular employee | 3.3 | 24.7 |  | 5.2 | 40.4 |  | 3.8 | 37.8 |  | 73.7 | 22.6 |  | 22.1 | 61.6 |  | 17.4 | 54.9 |  | 37.1 | 53.2 |  | 42.8 | 51.4 |  | 2.1 (1.6) |
| Part-time/temporary worker | 2.5 | 22.9 |  | 4.9 | 32.7 |  | 4.4 | 36.9 |  | 74.9 | 21.1 |  | 20.6 | 62.9 |  | 20.7 | 52.5 |  | 33.2 | 50.9 |  | 42.5 | 51.4 |  | 2 (1.7) |
| Business owner/family business | 1.3 | 16.5 |  | 3.2 | 29.7 |  | 2.5 | 25.3 |  | 73.1 | 25.0 |  | 20.3 | 65.8 |  | 18.9 | 47.2 |  | 52.5 | 36.9 |  | 48.1 | 42.9 |  | 2.2 (1.5) |
| Unemployed | 1.5 | 21.9 |  | 4.0 | 34.8 |  | 6.5 | 37.3 |  | 73.9 | 23.1 |  | 19.4 | 58.2 |  | 17.4 | 50.7 |  | 31.3 | 50.5 |  | 39.7 | 51.3 |  | 1.9 (1.5) |
| Student | 4.2 | 36.7 |  | 6.3 | 48.3 |  | 4.7 | 36.8 |  | 85.1 | 13.3 |  | 27.1 | 61.9 |  | 23.3 | 55.3 |  | 49.9 | 44.6 |  | 49.5 | 46.1 |  | 2.5 (1.7) |
| ***Annual Income (in JPY 10,000s)*** |  |  | < 0.001 |  |  | 0.003 |  |  | 0.078 |  |  | 0.123 |  |  | 0.001 |  |  | 0.021 |  |  | 0.003 |  |  | 0.032 |  |
| 0-99 | 3.4 | 30.4 |  | 5.9 | 43.2 |  | 5.3 | 37.9 |  | 78.7 | 18.5 |  | 25.0 | 59.6 |  | 21.0 | 53.0 |  | 43.3 | 46.1 |  | 45.8 | 48.1 |  | 2.3 (1.7) |
| 100-199 | 2.4 | 19.1 |  | 6.8 | 29.9 |  | 6.4 | 33.5 |  | 72.5 | 23.5 |  | 20.3 | 61.8 |  | 18.3 | 51.8 |  | 33.6 | 51.2 |  | 45.4 | 45.0 |  | 2.1 (1.7) |
| 200-299 | 2.9 | 19.0 |  | 4.6 | 34.4 |  | 2.7 | 36.7 |  | 72.6 | 22.5 |  | 23.1 | 56.5 |  | 17.8 | 54.6 |  | 39.1 | 50.2 |  | 45.5 | 48.4 |  | 2.1 (1.6) |
| 300-399 | 3.0 | 23.6 |  | 4.4 | 40.8 |  | 4.4 | 38.6 |  | 74.0 | 22.7 |  | 21.0 | 62.4 |  | 20.8 | 54.1 |  | 37.5 | 53.0 |  | 40.7 | 54.0 |  | 2.1 (1.6) |
| 400-499 | 4.0 | 28.8 |  | 6.5 | 39.3 |  | 3.4 | 33.9 |  | 77.7 | 19.8 |  | 22.0 | 67.8 |  | 17.0 | 52.3 |  | 35.7 | 52.5 |  | 42.7 | 52.0 |  | 2.1 (1.6) |
| 500-699 | 1.5 | 27.3 |  | 3.0 | 39.9 |  | 2.0 | 33.3 |  | 71.7 | 25.8 |  | 15.2 | 69.2 |  | 11.7 | 58.9 |  | 31.3 | 60.6 |  | 35.5 | 59.9 |  | 1.7 (1.4) |
| ≥700 | 8.9 | 26.7 |  | 4.4 | 42.2 |  | 2.2 | 28.9 |  | 73.3 | 22.2 |  | 28.9 | 66.7 |  | 17.8 | 35.6 |  | 46.7 | 48.9 |  | 33.3 | 60.0 |  | 2.2 (1.3) |
| **Preferred life course of wife** |  |  | 0.03 |  |  | 0.507 |  |  | 0.036 |  |  | 0.308 |  |  | 0.897 |  |  | 0.19 |  |  | < 0.001 |  |  | 0.059 |  |
| Working | 4.0 | 26.8 |  | 6.3 | 39.7 |  | 5.7 | 40.1 |  | 71.2 | 24.4 |  | 20.5 | 61.2 |  | 18.3 | 54.4 |  | 36.9 | 50.9 |  | 37.3 | 55.3 |  | 2.0 (1.7) |
| Homemaker | 2.3 | 26.0 |  | 4.3 | 41.4 |  | 2.8 | 35.1 |  | 79.8 | 18.3 |  | 24.7 | 61.5 |  | 19.5 | 53.4 |  | 42.5 | 49.4 |  | 50.6 | 45.4 |  | 2.3 (1.5) |
| **Region of residence** |  |  | 0.011 |  |  | 0.19 |  |  | 0.629 |  |  | 0.009 |  |  | < 0.001 |  |  | 0.829 |  |  | 0.656 |  |  | 0.13 |  |
| Hokkaido | 1.2 | 27.9 |  | 3.5 | 38.4 |  | 3.5 | 36.0 |  | 83.7 | 14.0 |  | 29.1 | 52.3 |  | 16.3 | 55.8 |  | 40.7 | 47.7 |  | 50.0 | 45.3 |  | 2.3 (1.5) |
| Tohoku | 1.1 | 18.1 |  | 4.9 | 39.6 |  | 6.0 | 39.6 |  | 68.7 | 28.0 |  | 20.3 | 58.8 |  | 16.5 | 52.7 |  | 32.4 | 53.8 |  | 37.0 | 57.5 |  | 1.9 (1.6) |
| Kanto | 4.6 | 27.7 |  | 6.2 | 41.3 |  | 4.9 | 37.3 |  | 78.0 | 19.2 |  | 24.3 | 61.3 |  | 20.1 | 53.8 |  | 40.4 | 49.7 |  | 43.0 | 51.3 |  | 2.2 (1.7) |
| Chubu | 2.1 | 25.0 |  | 4.3 | 40.4 |  | 3.3 | 35.8 |  | 73.8 | 22.2 |  | 18.7 | 61.9 |  | 18.6 | 53.4 |  | 38.1 | 51.6 |  | 43.8 | 49.2 |  | 2.0 (1.6) |
| Kinki | 2.5 | 25.7 |  | 4.2 | 34.7 |  | 3.3 | 33.5 |  | 75.8 | 21.3 |  | 23.8 | 63.0 |  | 19.0 | 51.5 |  | 39.9 | 48.3 |  | 44.8 | 49.5 |  | 2.1 (1.6) |
| Chugoku | 2.2 | 25.7 |  | 6.5 | 41.7 |  | 4.3 | 38.0 |  | 75.7 | 22.5 |  | 24.3 | 66.3 |  | 17.4 | 58.0 |  | 43.3 | 46.9 |  | 49.8 | 46.5 |  | 2.2 (1.6) |
| Kyushu | 3.4 | 24.0 |  | 5.1 | 36.1 |  | 4.4 | 37.8 |  | 69.9 | 23.6 |  | 19.7 | 57.3 |  | 20.6 | 51.0 |  | 36.8 | 52.0 |  | 42.8 | 48.1 |  | 2.0 (1.7) |
| ***Area of Residence: Population*** *Size* and Density |  |  | 0.044 |  |  | 0.024 |  |  | 0.025 |  |  | < 0.001 |  |  | 0.002 |  |  | 0.605 |  |  | 0.12 |  |  | 0.012 |  |
| Non-densely inhabited area | 2.4 | 22.9 |  | 4.9 | 38.9 |  | 5.0 | 38.1 |  | 68.6 | 26.9 |  | 20.0 | 60.9 |  | 18.3 | 53.7 |  | 36.6 | 50.8 |  | 42.7 | 50.0 |  | 2.0 (1.7) |
| <200,000 | 3.2 | 24.4 |  | 6.0 | 35.0 |  | 4.0 | 34.1 |  | 74.8 | 21.3 |  | 20.0 | 64.2 |  | 17.9 | 53.6 |  | 38.0 | 51.4 |  | 44.0 | 49.6 |  | 2.1 (1.6) |
| 200,000 to <1,000,000 | 3.6 | 29.0 |  | 5.3 | 44.1 |  | 4.4 | 39.8 |  | 79.6 | 17.9 |  | 24.9 | 60.9 |  | 19.7 | 54.8 |  | 42.2 | 48.6 |  | 48.2 | 47.4 |  | 2.3 (1.6) |
| ≥1,000,000 | 3.6 | 27.0 |  | 4.9 | 38.9 |  | 3.3 | 33.0 |  | 80.7 | 17.4 |  | 26.3 | 59.4 |  | 20.9 | 51.2 |  | 41.0 | 49.0 |  | 39.5 | 54.4 |  | 2.2 (1.7) |

**STable 23.** Factors listed as “important” or “would consider” when choosing a partner among previously-married women with marriage intention, aged 18-49 years, in the National Fertility Survey 2015 by sociodemographic variables. Numbers are shown in percent.

|  | **Education** | | | **Occupation** | | | **Finances** | | | **Personality** | | | **Appearance** | | | **Mutual hobbies** | | | **Cooperative regarding one’s work** | | | **Skills/attitude towards chores/childrearing** | | | **Mean (SD) important** |
| --- | --- | --- | --- | --- | --- | --- | --- | --- | --- | --- | --- | --- | --- | --- | --- | --- | --- | --- | --- | --- | --- | --- | --- | --- | --- |
|  | Important | Would consider | p | Important | Would consider | p | Important | Would consider | p | Important | Would consider | p | Important | Would consider | p | Important | Would consider | p | Important | Would consider | p | Important | Would consider | p |  |
| Total | 3.6 | 36.5 |  | 20.1 | 59.3 |  | 37.1 | 57.4 |  | 86.7 | 11.8 |  | 9.1 | 64.2 |  | 22.4 | 51.2 |  | 42.6 | 46.2 |  | 51.8 | 42.4 |  | 2.9 (2) |
| **Age group** |  |  | 0.457 |  |  | 0.209 |  |  | 0.659 |  |  | 0.111 |  |  | 0.636 |  |  | 0.34 |  |  | 0.19 |  |  | 0.023 |  |
| 18-24 | 0 | 12.5 |  | 12.5 | 50.0 |  | 25.0 | 75.0 |  | 62.5 | 37.5 |  | 25.0 | 62.5 |  | 25.0 | 50.0 |  | 50.0 | 50.0 |  | 75.0 | 25.0 |  | 2.9 (1.9) |
| 25-29 | 4.3 | 21.7 |  | 30.4 | 60.9 |  | 47.8 | 47.8 |  | 87.0 | 13.0 |  | 8.7 | 60.9 |  | 21.7 | 43.5 |  | 47.8 | 43.5 |  | 69.6 | 30.4 |  | 3.4 (2.3) |
| 30-34 | 5.1 | 32.2 |  | 23.7 | 59.3 |  | 40.7 | 57.6 |  | 91.5 | 8.5 |  | 11.9 | 67.8 |  | 20.3 | 49.2 |  | 50.8 | 40.7 |  | 69.5 | 30.5 |  | 3.3 (2.1) |
| 34-39 | 1.6 | 46.9 |  | 25.4 | 61.9 |  | 39.1 | 57.8 |  | 87.5 | 12.5 |  | 4.7 | 71.9 |  | 20.3 | 65.6 |  | 50.0 | 45.3 |  | 50.0 | 42.2 |  | 3 (2.1) |
| 40-44 | 3.2 | 35.1 |  | 17.0 | 54.3 |  | 36.2 | 55.3 |  | 88.3 | 7.4 |  | 8.5 | 60.6 |  | 27.7 | 42.6 |  | 39.8 | 43.0 |  | 43.5 | 47.8 |  | 2.8 (1.8) |
| 45-49 | 4.9 | 39.5 |  | 14.6 | 63.4 |  | 32.1 | 60.5 |  | 82.9 | 15.9 |  | 9.8 | 61.0 |  | 19.5 | 53.7 |  | 31.7 | 54.9 |  | 42.7 | 50.0 |  | 2.5 (1.9) |
| ***Education*** |  |  | 0.01 |  |  | 0.147 |  |  | 0.637 |  |  | 0.399 |  |  | 0.103 |  |  | 0.63 |  |  | 0.083 |  |  | 0.796 |  |
| High school or less | 3.8 | 28.6 |  | 20.5 | 54.1 |  | 39.1 | 54.9 |  | 84.3 | 14.1 |  | 8.6 | 64.3 |  | 21.6 | 49.2 |  | 36.4 | 50.5 |  | 53.6 | 41.0 |  | 2.8 (2) |
| Vocational school/ short college | 2.9 | 44.1 |  | 19.8 | 67.3 |  | 31.4 | 65.7 |  | 89.2 | 9.8 |  | 5.9 | 65.7 |  | 22.5 | 54.9 |  | 51.0 | 42.2 |  | 50.0 | 44.1 |  | 2.9 (2) |
| Undergraduate studies | 5.7 | 54.3 |  | 19.4 | 66.7 |  | 41.7 | 52.8 |  | 94.4 | 5.6 |  | 22.2 | 61.1 |  | 27.8 | 55.6 |  | 50.0 | 38.9 |  | 50.0 | 44.4 |  | 3.3 (2) |
| Graduate studies | 0 | 100 |  | 50.0 | 50.0 |  | 50.0 | 50.0 |  | 50.0 | 50.0 |  | 0 | 100 |  | 0 | 50.0 |  | 0 | 50.0 |  | 0 | 100 |  | 2 (2.8) |
| ***Occupational Status*** |  |  | 0.309 |  |  | 0.031 |  |  | 0.074 |  |  | 0.511 |  |  | 0.277 |  |  | 0.202 |  |  | < 0.001 |  |  | 0.092 |  |
| Regular employee | 3.8 | 41.2 |  | 24.2 | 60.6 |  | 42.7 | 55.0 |  | 88.6 | 9.8 |  | 12.1 | 62.9 |  | 27.3 | 53.0 |  | 54.5 | 40.2 |  | 59.1 | 37.1 |  | 3.3 (2) |
| Part-time/temporary worker | 3.7 | 36.8 |  | 22.2 | 56.3 |  | 39.0 | 55.1 |  | 86.8 | 12.5 |  | 5.9 | 65.4 |  | 19.1 | 52.2 |  | 35.3 | 51.5 |  | 50.0 | 44.1 |  | 2.8 (2) |
| Business owner/family business | 7.1 | 35.7 |  | 7.1 | 71.4 |  | 28.6 | 64.3 |  | 100 | 0 |  | 21.4 | 57.1 |  | 28.6 | 42.9 |  | 64.3 | 28.6 |  | 35.7 | 50.0 |  | 2.9 (1.8) |
| Unemployed | 0 | 21.2 |  | 3.0 | 60.6 |  | 15.2 | 75.8 |  | 78.8 | 18.2 |  | 3.0 | 69.7 |  | 9.1 | 57.6 |  | 12.5 | 62.5 |  | 32.3 | 61.3 |  | 1.5 (1.3) |
| Student | - | - |  | - | - |  | - | - |  | - | - |  | - | - |  | - | - |  | - | - |  | - | - |  | - |
| ***Annual Income (in JPY 10,000s)*** |  |  | 0.002 |  |  | 0.169 |  |  | 0.214 |  |  | 0.983 |  |  | 0.095 |  |  | 0.735 |  |  | 0.02 |  |  | 0.022 |  |
| 0-99 | 2.2 | 27.0 |  | 12.4 | 57.3 |  | 29.2 | 60.7 |  | 83.1 | 14.6 |  | 6.7 | 61.8 |  | 18.0 | 52.8 |  | 30.7 | 48.9 |  | 47.1 | 41.4 |  | 2.4 (1.9) |
| 100-199 | 3.3 | 32.2 |  | 22.5 | 56.2 |  | 37.1 | 57.3 |  | 87.8 | 11.1 |  | 4.4 | 72.2 |  | 21.1 | 48.9 |  | 40.0 | 50.0 |  | 51.1 | 46.7 |  | 2.9 (1.9) |
| 200-299 | 3.7 | 39.5 |  | 22.2 | 60.5 |  | 39.5 | 58 |  | 88.9 | 9.9 |  | 14.8 | 56.8 |  | 24.7 | 53.1 |  | 46.9 | 48.1 |  | 55.6 | 42.0 |  | 3.1 (2.1) |
| 300-399 | 4.3 | 55.3 |  | 25.5 | 63.8 |  | 44.7 | 53.2 |  | 85.1 | 12.8 |  | 6.4 | 72.3 |  | 25.5 | 48.9 |  | 51.1 | 38.3 |  | 57.4 | 40.4 |  | 3.2 (1.9) |
| 400-499 | 0 | 28.6 |  | 28.6 | 57.1 |  | 71.4 | 28.6 |  | 100 | 0 |  | 14.3 | 57.1 |  | 28.6 | 57.1 |  | 71.4 | 28.6 |  | 42.9 | 28.6 |  | 3.9 (2.1) |
| ≥500 | 22.2 | 66.7 |  | 30.0 | 70.0 |  | 50.0 | 40.0 |  | 90 | 10 |  | 30.0 | 60.0 |  | 30.0 | 70.0 |  | 70.0 | 30.0 |  | 40.0 | 40.0 |  | 3.7 (2.5) |
| **Preferred life course of wife** |  |  | 0.355 |  |  | 0.636 |  |  | 0.671 |  |  | 0.444 |  |  | 0.571 |  |  | 0.431 |  |  | 0.182 |  |  | 0.889 |  |
| Working | 5.1 | 38.2 |  | 22.8 | 59.6 |  | 35.3 | 61.0 |  | 89.7 | 9.6 |  | 11.0 | 64.0 |  | 25.7 | 49.3 |  | 48.1 | 43.7 |  | 51.5 | 42.5 |  | 3.1 (2.1) |
| Homemaker | 2.4 | 35.7 |  | 19.0 | 60.1 |  | 39.9 | 56.0 |  | 88.2 | 11.8 |  | 7.7 | 68.0 |  | 19.5 | 53.8 |  | 39.6 | 46.7 |  | 52.7 | 42.6 |  | 2.9 (1.9) |
| **Region of residence** |  |  | 0.101 |  |  | 0.86 |  |  | 0.42 |  |  | 0.78 |  |  | 0.01 |  |  | 0.536 |  |  | 0.105 |  |  | 0.7 |  |
| Hokkaido | 0 | 15.4 |  | 30.8 | 61.5 |  | 30.8 | 61.5 |  | 84.6 | 7.7 |  | 7.7 | 38.5 |  | 7.7 | 46.2 |  | 53.8 | 15.4 |  | 46.2 | 38.5 |  | 2.9 (2.5) |
| Tohoku | 4.3 | 21.7 |  | 10.9 | 63.0 |  | 32.6 | 60.9 |  | 84.8 | 13.0 |  | 2.2 | 54.3 |  | 21.7 | 56.5 |  | 34.8 | 52.2 |  | 51.1 | 37.8 |  | 2.5 (1.6) |
| Kanto | 6.3 | 40.0 |  | 19.8 | 59.3 |  | 34.6 | 54.3 |  | 82.7 | 14.8 |  | 11.1 | 66.7 |  | 22.2 | 49.4 |  | 38.3 | 49.4 |  | 48.1 | 45.7 |  | 2.8 (1.9) |
| Chubu | 0 | 45.9 |  | 24.6 | 55.7 |  | 41.7 | 55.0 |  | 88.5 | 9.8 |  | 11.5 | 68.9 |  | 19.7 | 47.5 |  | 36.1 | 49.2 |  | 49.2 | 45.9 |  | 3.0 (2.0) |
| Kinki | 5.9 | 35.3 |  | 21.6 | 54.9 |  | 45.1 | 52.9 |  | 92.2 | 7.8 |  | 15.7 | 58.8 |  | 27.5 | 58.8 |  | 54.9 | 39.2 |  | 58.8 | 39.2 |  | 3.4 (2.2) |
| Chugoku | 4.0 | 52.0 |  | 16.0 | 60.0 |  | 44.0 | 52.0 |  | 88.0 | 12.0 |  | 4.0 | 56.0 |  | 24.0 | 56.0 |  | 36.0 | 52.0 |  | 52.0 | 40.0 |  | 2.8 (1.6) |
| Kyushu | 1.9 | 32.1 |  | 21.2 | 63.5 |  | 30.2 | 67.9 |  | 86.8 | 13.2 |  | 5.7 | 79.2 |  | 24.5 | 45.3 |  | 51.9 | 44.2 |  | 55.8 | 42.3 |  | 2.9 (2.1) |
| ***Area of Residence: Population*** *Size* and Density |  |  | 0.341 |  |  | 0.577 |  |  | 0.655 |  |  | 0.286 |  |  | 0.002 |  |  | 0.455 |  |  | 0.77 |  |  | 0.104 |  |
| Non-densely inhabited area | 2.6 | 32.8 |  | 15.7 | 59.1 |  | 39.1 | 56.5 |  | 85.3 | 13.8 |  | 3.4 | 67.2 |  | 19.0 | 56.0 |  | 44.0 | 47.4 |  | 49.6 | 46.1 |  | 2.7 (1.8) |
| <200,000 | 4.6 | 35.4 |  | 24.6 | 58.5 |  | 30.8 | 63.1 |  | 83.1 | 12.3 |  | 10.8 | 55.4 |  | 16.9 | 53.8 |  | 35.4 | 52.3 |  | 53.8 | 36.9 |  | 2.8 (2.0) |
| 200,000 to <1,000,000 | 3.1 | 45.8 |  | 23.7 | 58.8 |  | 37.1 | 58.8 |  | 88.7 | 11.3 |  | 16.5 | 69.1 |  | 27.8 | 48.5 |  | 43.3 | 43.3 |  | 50.5 | 47.4 |  | 3.1 (2.1) |
| ≥1,000,000 | 5.8 | 28.8 |  | 17.3 | 61.5 |  | 40.4 | 50.0 |  | 90.4 | 7.7 |  | 5.8 | 59.6 |  | 26.9 | 42.3 |  | 47.1 | 41.2 |  | 56.9 | 31.4 |  | 3.0 (1.9) |

**STable 24.** Factors listed as “important” or “would consider” when choosing a partner among previously-married men with marriage intention, aged 18-49 years, in the National Fertility Survey 2015 by sociodemographic variables. Numbers are shown in percent.

|  | **Education** | | | **Occupation** | | | **Finances** | | | **Personality** | | | **Appearance** | | | **Mutual hobbies** | | | **Cooperative regarding one’s work** | | | **Skills/attitude towards chores/childrearing** | | | **Mean (SD) important** |
| --- | --- | --- | --- | --- | --- | --- | --- | --- | --- | --- | --- | --- | --- | --- | --- | --- | --- | --- | --- | --- | --- | --- | --- | --- | --- |
|  | Important | Would consider | p | Important | Would consider | p | Important | Would consider | p | Important | Would consider | p | Important | Would consider | p | Important | Would consider | p | Important | Would consider | p | Important | Would consider | p |  |
| Total | 1.5 | 25.4 |  | 4.6 | 27.4 |  | 3.1 | 29.6 |  | 72.7 | 21.7 |  | 22.3 | 65.5 |  | 17.8 | 50.3 |  | 38.1 | 52.8 |  | 44.2 | 50.8 |  | 2.1 (1.6) |
| **Age group** |  |  | 0.597 |  |  | 0.276 |  |  | 0.371 |  |  | 0.671 |  |  | 0.038 |  |  | 0.902 |  |  | 0.213 |  |  | 0.793 |  |
| 18-24 | 0 | 0 |  | 0 | 0 |  | 0 | 0 |  | 100.0 | 0 |  | 0 | 0 |  | 0 | 0 |  | 0 | 100 |  | 0 | 100 |  | 1.0 (0) |
| 25-29 | 0 | 40.0 |  | 0 | 80.0 |  | 0 | 60.0 |  | 80.0 | 20.0 |  | 0 | 100 |  | 20.0 | 40.0 |  | 40.0 | 40.0 |  | 60.0 | 40.0 |  | 2.0 (1.6) |
| 30-34 | 0 | 8.7 |  | 4.3 | 13.0 |  | 8.7 | 17.4 |  | 69.6 | 21.7 |  | 4.3 | 73.9 |  | 26.1 | 39.1 |  | 47.8 | 52.2 |  | 47.8 | 47.8 |  | 2.1 (1.6) |
| 34-39 | 0 | 22.4 |  | 2.0 | 26.5 |  | 0 | 37.5 |  | 86.0 | 10.0 |  | 28.6 | 59.2 |  | 18.4 | 55.1 |  | 42.9 | 51.0 |  | 44.9 | 53.1 |  | 2.2 (1.4) |
| 40-44 | 1.8 | 30.9 |  | 7.3 | 30.9 |  | 5.5 | 27.3 |  | 65.5 | 27.3 |  | 25.5 | 60.0 |  | 16.4 | 50.9 |  | 30.9 | 63.6 |  | 34.5 | 58.2 |  | 1.9 (1.8) |
| 45-49 | 3.1 | 28.1 |  | 4.7 | 26.6 |  | 1.6 | 28.1 |  | 68.8 | 26.6 |  | 23.4 | 70.3 |  | 15.6 | 51.6 |  | 37.5 | 45.3 |  | 50.0 | 43.8 |  | 2.1 (1.7) |
| ***Education*** |  |  | 0.223 |  |  | 0.16 |  |  | 0.404 |  |  | 0.909 |  |  | 0.085 |  |  | 0.385 |  |  | 0.818 |  |  | 0.063 |  |
| High school or less | 2.5 | 21.0 |  | 4.2 | 25.2 |  | 2.5 | 30.3 |  | 73.1 | 20.2 |  | 26.1 | 58.8 |  | 18.5 | 52.1 |  | 40.3 | 52.9 |  | 48.3 | 47.5 |  | 2.2 (1.7) |
| Vocational school/ short college | 0 | 22.9 |  | 0 | 22.9 |  | 0 | 32.4 |  | 66.7 | 27.8 |  | 11.4 | 85.7 |  | 20.0 | 40.0 |  | 37.1 | 51.4 |  | 25.0 | 72.2 |  | 1.6 (1.1) |
| Undergraduate studies | 0 | 39.5 |  | 10.5 | 36.8 |  | 7.9 | 28.9 |  | 76.3 | 21.1 |  | 23.7 | 63.2 |  | 15.8 | 57.9 |  | 34.2 | 52.6 |  | 50.0 | 39.5 |  | 2.3 (1.9) |
| Graduate studies | 0 | 50.0 |  | 0 | 50.0 |  | 0 | 0 |  | 75.0 | 25.0 |  | 0 | 100 |  | 0 | 25 |  | 25.0 | 75.0 |  | 25.0 | 75.0 |  | 1.3 (1.3) |
| ***Occupational Status*** |  |  | 0.241 |  |  | 0.722 |  |  | 0.861 |  |  | 0.528 |  |  | 0.494 |  |  | 0.59 |  |  | 0.624 |  |  | 0.489 |  |
| Regular employee | 0 | 29.7 |  | 4.2 | 28.8 |  | 3.4 | 29.1 |  | 72.0 | 22.0 |  | 20.3 | 68.6 |  | 16.9 | 55.1 |  | 34.7 | 55.9 |  | 43.2 | 52.5 |  | 2.0 (1.5) |
| Part-time/temporary worker | 4.5 | 18.2 |  | 4.5 | 22.7 |  | 4.5 | 31.8 |  | 86.4 | 13.6 |  | 22.7 | 72.7 |  | 13.6 | 54.5 |  | 27.3 | 59.1 |  | 50.0 | 50.0 |  | 2.1 (1.7) |
| Business owner/family business | 3.3 | 13.3 |  | 0 | 26.7 |  | 0 | 20.0 |  | 63.3 | 26.7 |  | 30.0 | 53.3 |  | 16.7 | 43.3 |  | 43.3 | 50.0 |  | 40.0 | 46.7 |  | 1.9 (1.7) |
| Unemployed | 6.7 | 26.7 |  | 13.3 | 26.7 |  | 6.7 | 33.3 |  | 87.5 | 12.5 |  | 20.0 | 60.0 |  | 20.0 | 46.7 |  | 53.3 | 33.3 |  | 43.8 | 50.0 |  | 2.5 (1.9) |
| Student | 0 | 50.0 |  | 0 | 50 |  | 0 | 50.0 |  | 100 | 0 |  | 0 | 50 |  | 0 | 0 |  | 0 | 100 |  | 0 | 100 |  | 1.0 (0) |
| ***Annual Income (in JPY 10,000s)*** |  |  | 0.265 |  |  | 0.503 |  |  | 0.42 |  |  | 0.334 |  |  | 0.758 |  |  | 0.153 |  |  | 0.607 |  |  | 0.379 |  |
| 0-99 | 6.3 | 25.0 |  | 12.5 | 25.0 |  | 6.3 | 40.6 |  | 75.8 | 21.2 |  | 21.9 | 59.4 |  | 28.1 | 37.5 |  | 53.1 | 40.6 |  | 53.1 | 43.8 |  | 2.6 (2) |
| 100-199 | 0 | 12.5 |  | 0 | 12.5 |  | 0 | 37.5 |  | 62.5 | 37.5 |  | 12.5 | 62.5 |  | 12.5 | 12.5 |  | 12.5 | 75.0 |  | 25.0 | 75.0 |  | 1.3 (1.3) |
| 200-299 | 0 | 23.5 |  | 2.9 | 20.6 |  | 2.9 | 26.5 |  | 76.5 | 14.7 |  | 17.6 | 70.6 |  | 8.8 | 58.8 |  | 32.4 | 55.9 |  | 44.1 | 50.0 |  | 1.9 (1.5) |
| 300-399 | 0 | 25.0 |  | 0 | 29.5 |  | 0 | 34.1 |  | 61.4 | 34.1 |  | 25.0 | 63.6 |  | 18.2 | 56.8 |  | 36.4 | 50.0 |  | 38.6 | 54.5 |  | 1.8 (1.3) |
| 400-499 | 0 | 16.7 |  | 3.3 | 30.0 |  | 3.4 | 20.7 |  | 70.0 | 20.0 |  | 13.3 | 76.7 |  | 13.3 | 50.0 |  | 33.3 | 63.3 |  | 26.7 | 70.0 |  | 1.7 (1.6) |
| 500-699 | 2.8 | 30.6 |  | 5.6 | 33.3 |  | 2.8 | 33.3 |  | 77.8 | 19.4 |  | 27.8 | 66.7 |  | 16.7 | 55.6 |  | 44.4 | 47.2 |  | 55.6 | 41.7 |  | 2.4 (1.6) |
| ≥700 | 0 | 54.5 |  | 9.1 | 36.4 |  | 9.1 | 0 |  | 100 | 0 |  | 36.4 | 54.5 |  | 36.4 | 45.5 |  | 27.3 | 63.6 |  | 63.6 | 27.3 |  | 2.9 (1.3) |
| **Preferred life course of wife** |  |  | 0.355 |  |  | 0.636 |  |  | 0.671 |  |  | 0.444 |  |  | 0.571 |  |  | 0.431 |  |  | 0.182 |  |  | 0.889 |  |
| Working | 1.3 | 20.5 |  | 7.7 | 21.8 |  | 3.9 | 29.9 |  | 70.5 | 24.4 |  | 23.1 | 65.4 |  | 16.7 | 46.2 |  | 35.9 | 52.6 |  | 42.3 | 48.7 |  | 2.1 (1.8) |
| Homemaker | 2.0 | 30.0 |  | 3.0 | 34.0 |  | 3.0 | 31.0 |  | 77.2 | 18.8 |  | 21.0 | 68.0 |  | 18.0 | 56.0 |  | 40.0 | 54.0 |  | 46.5 | 53.5 |  | 2.1 (1.4) |
| **Region of residence** |  |  | 0.05 |  |  | 0.812 |  |  | 0.322 |  |  | 0.293 |  |  | 0.21 |  |  | 0.598 |  |  | 0.967 |  |  | 0.216 |  |
| Hokkaido | 11.1 | 11.1 |  | 11.1 | 0 |  | 11.1 | 11.1 |  | 100 | 0 |  | 44.4 | 55.6 |  | 33.3 | 55.6 |  | 44.4 | 44.4 |  | 77.8 | 22.2 |  | 3.3 (1.9) |
| Tohoku | 0 | 29.4 |  | 5.9 | 17.6 |  | 0 | 35.3 |  | 70.6 | 23.5 |  | 29.4 | 47.1 |  | 17.6 | 52.9 |  | 29.4 | 64.7 |  | 23.5 | 70.6 |  | 1.8 (1.6) |
| Kanto | 0 | 36.4 |  | 5.5 | 30.9 |  | 5.5 | 38.2 |  | 70.9 | 25.5 |  | 23.6 | 67.3 |  | 18.2 | 49.1 |  | 38.2 | 54.5 |  | 50.9 | 47.3 |  | 2.2 (1.7) |
| Chubu | 5.6 | 8.3 |  | 2.8 | 25.0 |  | 5.6 | 16.7 |  | 77.8 | 11.1 |  | 19.4 | 63.9 |  | 19.4 | 44.4 |  | 44.4 | 47.2 |  | 44.4 | 44.4 |  | 2.2 (1.6) |
| Kinki | 0 | 24.1 |  | 3.4 | 31.0 |  | 0 | 35.7 |  | 76.7 | 23.3 |  | 34.5 | 55.2 |  | 10.3 | 69.0 |  | 31.0 | 51.7 |  | 51.7 | 44.8 |  | 2.1 (1.5) |
| Chugoku | 0 | 30.4 |  | 4.3 | 39.1 |  | 0 | 26.1 |  | 73.9 | 17.4 |  | 4.3 | 82.6 |  | 17.4 | 52.2 |  | 39.1 | 52.2 |  | 30.4 | 65.2 |  | 1.7 (1.4) |
| Kyushu | 0 | 25.0 |  | 3.6 | 25.0 |  | 0 | 28.6 |  | 57.1 | 35.7 |  | 14.3 | 75.0 |  | 17.9 | 35.7 |  | 39.3 | 53.6 |  | 35.7 | 57.1 |  | 1.7 (1.6) |
| ***Area of Residence: Population*** *Size* and Density |  |  | 0.122 |  |  | 0.125 |  |  | 0.133 |  |  | 0.191 |  |  | 0.079 |  |  | 0.428 |  |  | 0.008 |  |  | 0.148 |  |
| Non-densely inhabited area | 2.9 | 22.9 |  | 1.4 | 31.4 |  | 1.4 | 25.7 |  | 68.6 | 27.1 |  | 12.9 | 74.3 |  | 18.6 | 54.3 |  | 38.6 | 61.4 |  | 36.2 | 60.9 |  | 1.8 (1.5) |
| <200,000 | 0 | 30.8 |  | 3.8 | 25.0 |  | 0 | 38.5 |  | 71.2 | 26.9 |  | 25.0 | 65.4 |  | 9.6 | 55.8 |  | 32.7 | 57.7 |  | 44.2 | 51.9 |  | 1.9 (1.5) |
| 200,000 to <1,000,000 | 0 | 14.9 |  | 4.3 | 21.3 |  | 6.5 | 32.6 |  | 79.2 | 10.4 |  | 21.3 | 63.8 |  | 21.3 | 40.4 |  | 36.2 | 44.7 |  | 45.8 | 43.8 |  | 2.2 (1.5) |
| ≥1,000,000 | 3.6 | 39.3 |  | 14.3 | 32.1 |  | 7.1 | 17.9 |  | 75.0 | 17.9 |  | 42.9 | 46.4 |  | 25.0 | 46.4 |  | 50.0 | 35.7 |  | 60.7 | 35.7 |  | 2.9 (2.0) |

**SFigure 1.** Ideal age of marriage and ideal age of partner among unmarried Japanese women and men, aged 18-49 years, with marriage intention and stratified by presence and absence of previous marriage. Red boxes indicate average ideal age of partner for any given ideal age of marriage (only calculated if n ≥10).


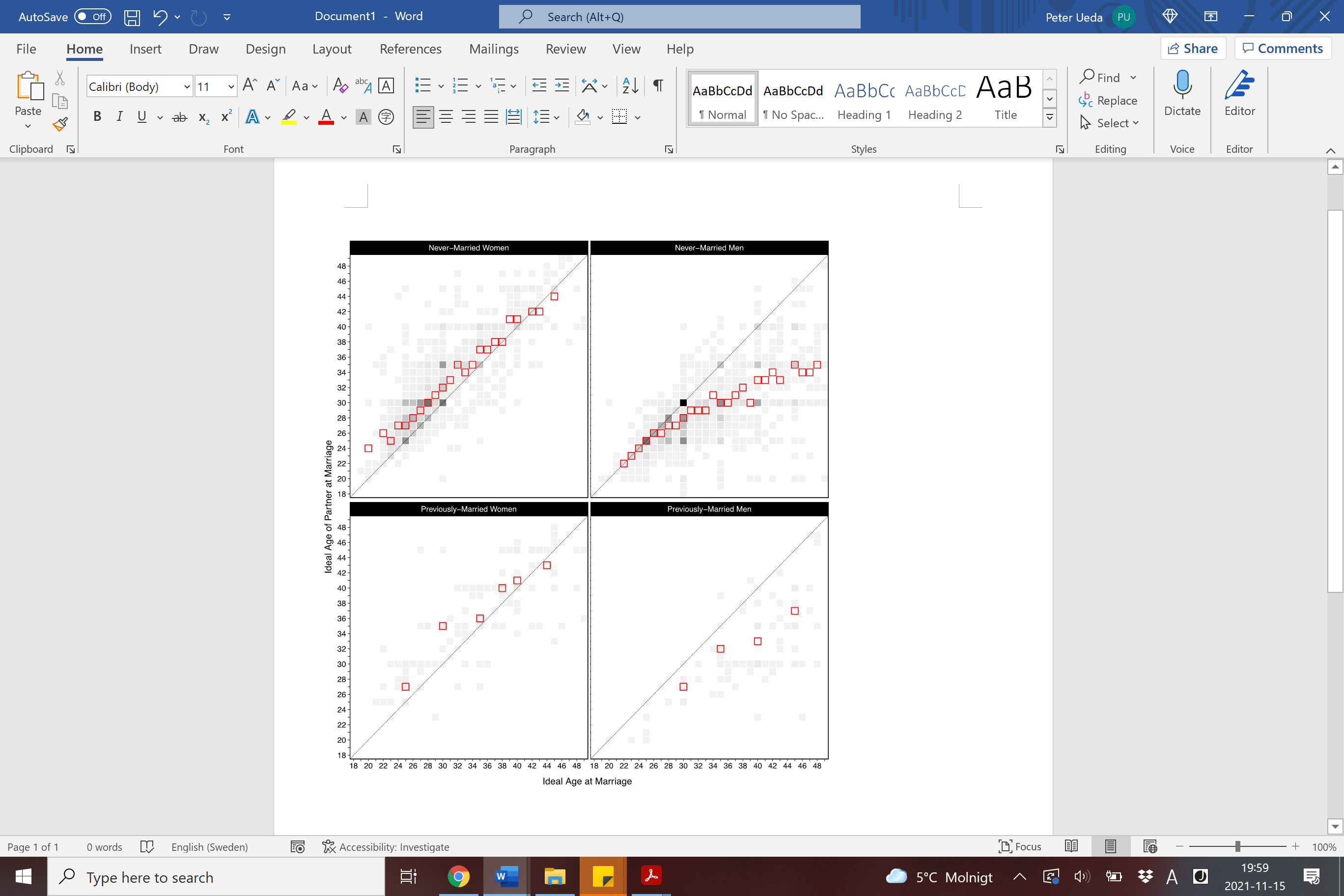


**SFigure 2.** Actual age of marriage and actual age of partner at marriage among married Japanese women and men, aged 18-49 years at time of marriage. The color of each box indicates the number of couples in the National Fertility Survey.


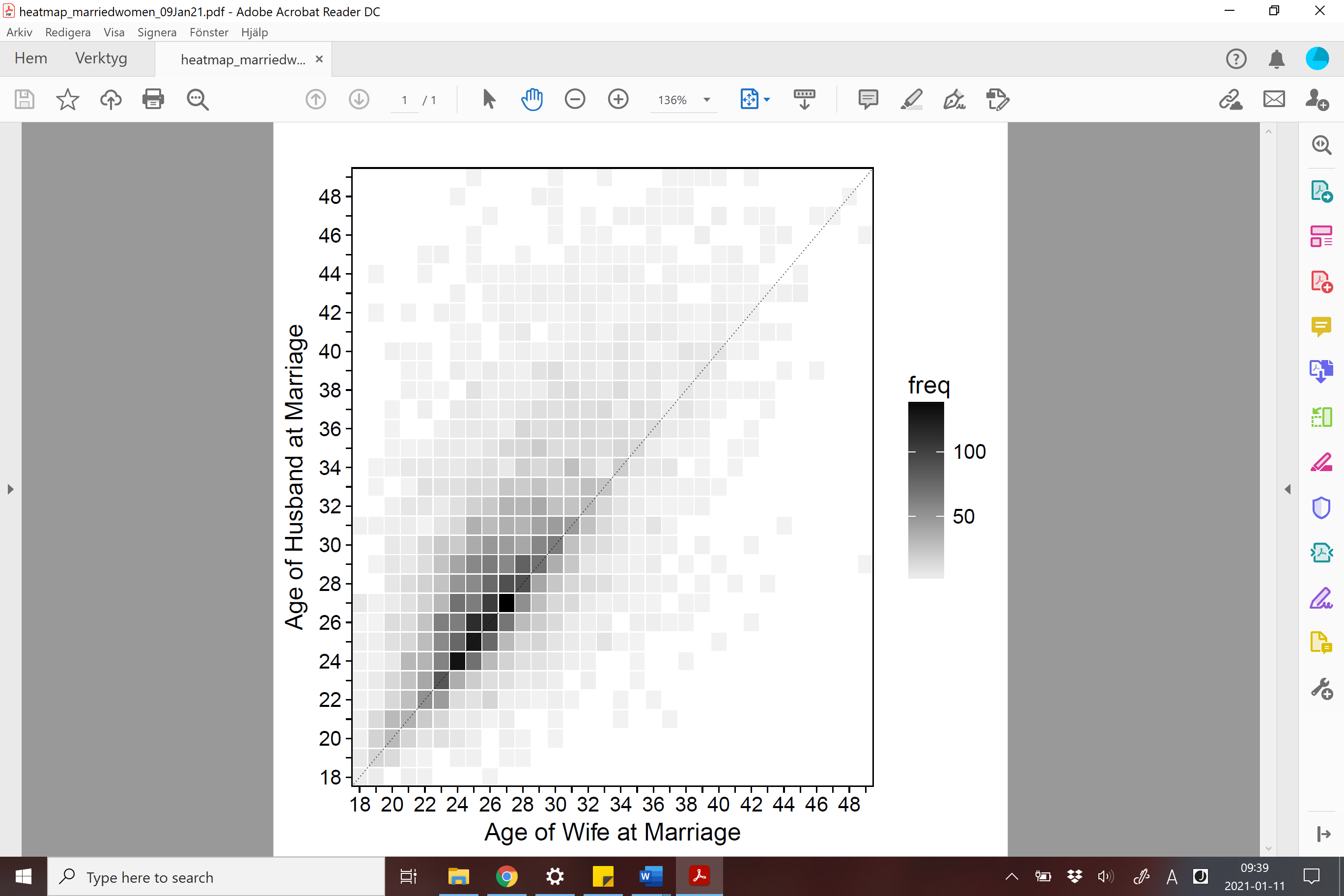


**References**

1 National Institute of Population and Social Security Research. The Fifteenth Japanese National Fertility Survey in 2015. Marriage Process and Fertility of Married Couples Attitudes toward Marriage and Family among Japanese Singles. Summary of the Survey Results on Married Couples/Singles. 2017 http://www.ipss.go.jp/ps-doukou/j/doukou15/doukou15_gaiyo.asp (accessed June 22, 2018).

2 Statistics Japan. Statistics Bureau, Ministry of Internal Affairs and Communications. What is a Densely Inhabited District? http://www.stat.go.jp/english/data/chiri/did/1-1.html (accessed June 25, 2018).
